# ACPC-MAP: a protocol for manually aligning structural T1-weighted magnetic resonance images to the anterior commissure – posterior commissure plane according to clinical standards

**DOI:** 10.1007/s00429-026-03171-z

**Published:** 2026-08-29

**Authors:** Ashok K. Selvaraj, Ronald H. M. A. Bartels, Christian F. Beckmann, R. Saman Vinke, Koen V. Haak

**Affiliations:** 1 Department of Neurosurgery, Radboud University Medical Center, Nijmegen, The Netherlands; 2 Donders Institute for Brain, Cognition, and Behavior, Radboud University, Nijmegen, The Netherlands; 3 Department of Cognitive Neuroscience, Radboud University Medical Center, Nijmegen, The Netherlands; 4 Centre for Functional MRI of the Brain, Nuffield Department of Clinical Neurosciences, Wellcome Centre for Integrative Neuroimaging, University of Oxford, Oxford, UK; 5 Department of Intelligent Systems, Center for Cognitive Science and Artificial Intelligence, Tilburg School of Humanities and Digital Sciences, Tilburg University, Tilburg, The Netherlands

**Keywords:** AC-PC Alignment, AC-PC Realignment, Manual MRI Realignment, AC-PC Guidelines, AC-PC Standardization protocol, Neuroimaging alignment, Stereotactic spaces

## Abstract

Realigning structural MR images to stereotactic AC-PC space is a standard procedure that enhances anatomical consistency both within and across neuroimaging studies, allowing for precise spatial localization and reliable cross-subject comparisons in both clinical and research contexts. However, different versions of AC-PC spaces, stemming from varying definitions of the AC-PC axis as outlined in common stereotactic atlases like Talairach and Schaltenbrand, often lead to discrepancies between the neurosurgical and neuroimaging communities. Manual realignment of structural MR images to the clinically used version of the AC-PC plane is often necessary to validate the results of automated methods or to realign the image when these methods fail. Furthermore, manual realignment provides a critical ‘ground truth’ for the development of new automated tools. However, such manual interventions are typically performed in a non-standardised manner by domain experts who possess the specialized knowledge of neuroanatomy and neuroimaging required to ensure accurate alignment. To address these challenges, we have developed and validated a standardized protocol for manually realigning structural MR images to the clinically used Schaltenbrand AC-PC plane, using a set of visual criteria to ensure accurate realignment. This protocol can be used to manually align images, verify results, validate existing automated methods, or generate ground truth data for developing new automated techniques.

**Supplementary Information:**

The online version contains supplementary material available at https://doi.org/10.1007/s00429-026-03171-z.

## Introduction

Realignment of magnetic resonance imaging (MRI) data to the anterior commissure–posterior commissure (AC–PC) plane provides a standardized anatomical orientation that facilitates systematic navigation through the brain and enables a clearer, anatomically consistent representation of brain structure and function (Coenen et al. 2008; Fox et al. 1984; Minoshima et al. 1993). AC–PC alignment has therefore become a fundamental preparatory step in stereotactic neurosurgery, where precise spatial orientation is essential for accurate surgical targeting (Bejjani et al. 2000; Mottonen et al. 2015; Starr et al. 1999). Beyond its clinical use, AC–PC alignment is also central to stereotactic neurosurgery research because it provides a common spatial framework for anatomical localization, methodological consistency, and the communication of findings, including the reporting of electrode locations after deep brain stimulation (DBS) surgery (Caire et al. 2013; Hebb and Miller 2010; Horn et al. 2017; Hyam et al. 2015).

Beyond stereotactic neurosurgery, AC–PC alignment plays an important role across diverse areas of neuroimaging research. Its incorporation into standard preprocessing workflows of major neuroimaging software packages reflects its broad methodological importance (Davis et al. 2021; Hamilton et al. 2017; Mandal et al. 2023). More fundamentally, AC–PC realignment standardizes image representation by placing individual brain anatomy within a consistent spatial framework, thereby improving the reliability and reproducibility of neuroimaging analyses (Choi et al. 2013; Park et al. 2010; Weiss et al. 2003). This standardized orientation supports several key neuroimaging applications, including image segmentation, registration, longitudinal imaging analysis, and the construction of standardized reference spaces (Ardekani & Alzheimer’s Disease Neuroimaging, 2022; Pai et al. 2020; Shah et al. 2022; Thompson et al. 2011). For example, Pai et al. developed a population-specific brain template using 113 AC–PC-aligned images, illustrating the value of this alignment procedure for generating consistent anatomical templates (Pai et al. 2020). The importance of standardized alignment is further supported by recent diffusion imaging studies showing that AC–PC realignment can reduce orientation-related variability and improve the robustness of subsequent analyses (Dautkulova et al. 2025; Kok et al. 2022; Ouachikh et al. 2024).

The need for spatial standardization has become even more important with the rapid growth of machine learning and deep learning methods in neuroimaging. Applications such as diagnostic classification, predictive modeling, automated image quality assessment, and segmentation depend on consistent anatomical positioning to generate accurate, reproducible, and interpretable results. In this context, AC–PC alignment remains a key initial preprocessing step that enhances the reliability and comparability of downstream neuroimaging analyses (Alkan and Cocjin 2017; Amorosino et al. 2022; Martensson et al. 2019; Qasim Abbas et al. 2023; Ryan et al. 2025; Thung et al. 2017; Zhou et al. 2019).

The AC-PC space, frequently referenced in the literature as ‘stereotactic space’, denotes the coordinate system in which anatomical data are embedded (Lau 2022). This space is commonly described using spatial Cartesian (x, y, z) coordinates relative to a fixed origin, defined by either the AC, the PC, or the midpoint between them, the midcommissural point (MCP) (Alaminos Bouza 2014; Bejjani et al. 2000; Fox and Kall 1987; Horn et al. 2017; Kandelʹ and Walker 1989). Atlases created according to this principle are known as stereotactic atlases. Two widely used stereotactic atlases in clinical neuroscience are the Talairach and Schaltenbrand atlases.

The widely recognized Talairach atlas, developed by Jean Talairach, served as a crucial resource for targeting deep brain structures through an AC-PC based coordinate system (Talairach and Tournoux 1988). Although revolutionary at the time, this atlas was prepared from a single cadaveric brain and had important limitations, such as limited orientation (sagittal and coronal) and an assumption of symmetrical hemispheres (Evans et al. 2012; Mazziotta et al. 1995). Another significant contribution came from Georges Schaltenbrand, who developed a groundbreaking stereotactic atlas using 111 brains (Schaltenbrand et al. 1977). This atlas provides comprehensive representation in all three anatomical planes, effectively addressing the limitations of the Talairach atlas (Lau 2022).

With the advent of MRI, the neuroimaging community sought to develop a common brain template based on structural MR images that represents the entire population rather than a single brain, as in the Talairach atlas (Evans et al. 2012). The Montreal Neurological Institute (MNI) template or MNI305,was developed as an attempt to create a common stereotactic space using 305 high-resolution MR images (Evans et al. 2012). The original T1-weighted images were realigned to approximate the Talairach-defined AC–PC stereotaxic space, following the manual identification of the AC, PC and other neuroanatomical landmarks (Evans et al. 1993). These realigned images were then averaged to create an initial template, which served as the basis for registering the original T1 images to develop the MNI 305 template (Collins et al. 1994; Evans et al. 1992, 1993). Since then, there has been continuous development in MNI templates (Evans et al. 2012), and the International Consortium for Brain Mapping (ICBM) has currently adopted the MNI152 image as a standard reference template for human brain mapping (Brett et al. 2002; Chau and McIntosh 2005).

**Fig. 1:**
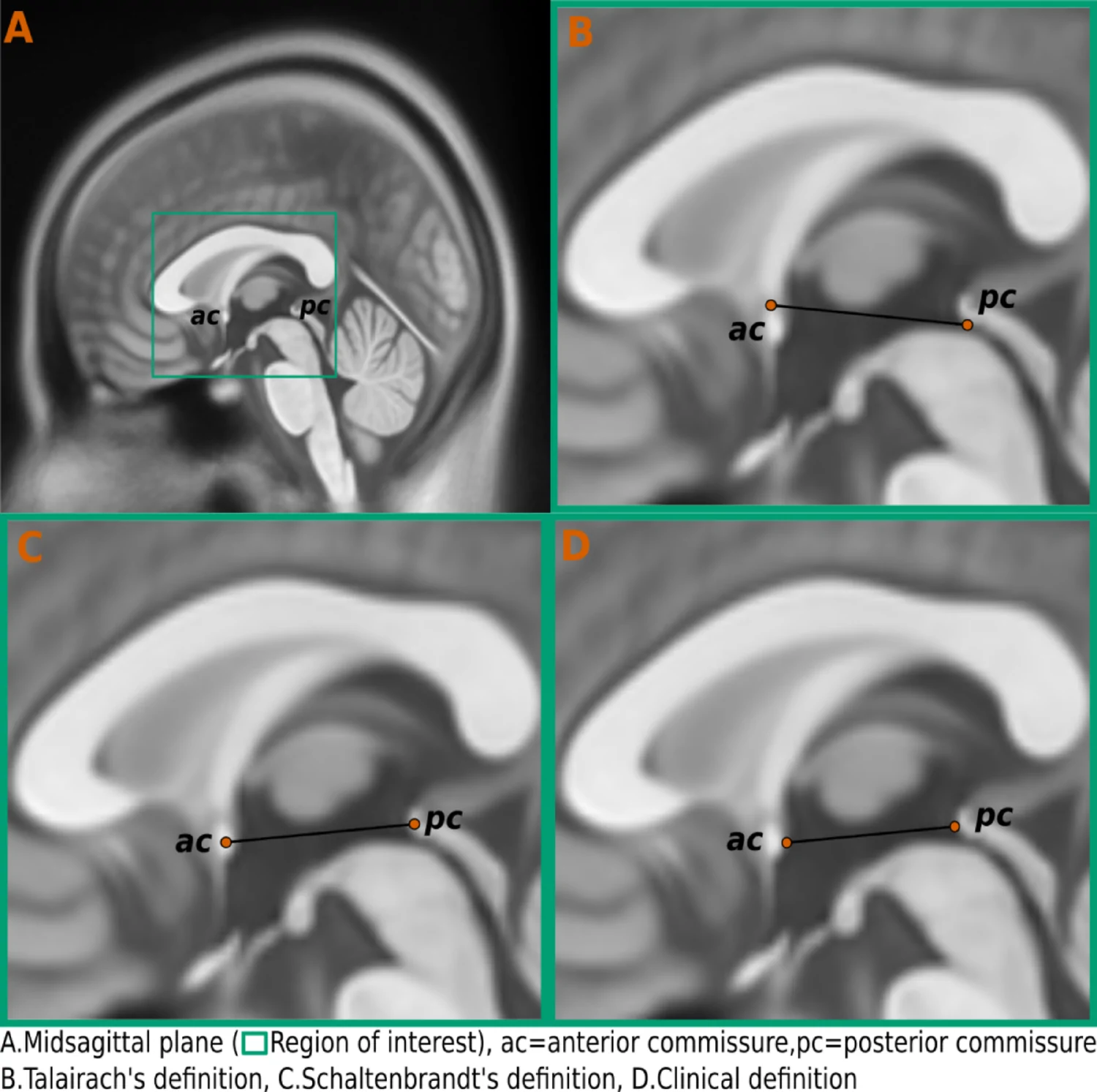
Definitions of the AC-PC line. This illustration presents the various interpretations of the AC-PC line that are commonly used within the neuroscience community. **A** Midsagittal plane. The box indicates the region of interest shown in (**B**-**D)**. AC: Anterior Commissure. PC: Posterior Commissure. **B** Talairach’s definition. **C** Schaltenbrand’s definition. **D** Clinical definition

The concurrent use of three distinct stereotactic spaces Talairach, Schaltenbrand, and MNI152 has led to inconsistencies within the clinical neuroscience community (Horn et al. 2017). This issue is further complicated by the fact that the neurosurgical community, which predominantly uses the Schaltenbrand-defined AC–PC space, has introduced slight modifications to the definition of the AC–PC line. A comprehensive comparison of these AC–PC spaces is presented in Table 1; Fig. 1.

**Table 1:** Comparison of the three commonly employed stereotactic spaces

Stereotactic Space	AC-PC line definition	Origin	Coordinate system	Scaling
Talairach	From the superior edge of the AC to the inferior edge of the PC (Fig. 1B)	The point where the tangential AC-PC line intersects with the vertical line drawn to the posterior border of the AC.	+ x is right, +y is anterior, +z is superior	Piecewise linear scaling
Schaltenbrand	From the centre of the AC to the centre of the PC (Fig. 1 C)	The point located at the midpoint of the AC-PC line, known as the midcommissural point (MCP).	+ x is right, +y is anterior, +z is superior	No scaling
MNI	The MNI templates adopted an approximation of Talairach’s AC-PC line definition; however, no explicit AC-PC line definition is required to realign the image to MNI space.	The point is located 3.5 mm above the original Talairach origin.	+ x is right, +y is anterior, +z is superior	Linear Scaling

The term “AC-PC realignment” fundamentally refers to the process of aligning the structural MR image to a standard orientation. In this framework, the Y-axis is defined by the AC-PC line, running from the AC to the PC. The Z-axis is oriented perpendicular to the AC-PC line, extending from superior to inferior. The X-axis, which runs from left to right, is defined as being perpendicular to the Y and Z axes. Both Y and Z axes are aligned within the longitudinal fissure, or the midline of the brain (Cox 1996; Lancaster et al. 1995). Although these orientations are broadly similar across stereotactic spaces, the definition of the AC–PC line and the approach used to achieve alignment vary across systems (Cox 1996; Glasser et al. 2013; Horn et al. 2017; Saint-Cyr et al. 2002). As a result, AC–PC realignment may be interpreted differently depending on the field of application, particularly in neuroimaging and neurosurgery.

In the context of the Talairach space, AC–PC realignment is often implemented as a two-stage process known as the Talairach transformation. This process begins with rotating the image to align it with the AC-PC plane. Following this initial alignment, the image is then rescaled to correspond with the anatomical landmarks of the Talairach atlas brain (Brett et al. 2002; Cox 1996). In stereotactic neurosurgery, AC–PC realignment typically involves rotating the image in the three cardinal planes so that the AC–PC appear at the same level in the horizontal plane, as defined by the Schaltenbrand atlas (Caire et al. 2011; Saint-Cyr et al. 2002). In contrast, within the neuroimaging community, “AC–PC realignment” often refers to rigid-body registration of a MRI image to a standardized stereotactic template, most commonly MNI152.This process utilizes 6 degrees of freedom (DOF) for rotation and translation, which are derived from an initial 12 DOF registration process (Glasser et al. 2013).

Although most researchers in the neuroimaging community have transitioned from Talairach space to MNI stereotactic space for anatomical standardization, the neurosurgical community continues to rely largely on Schaltenbrand space (Horn et al. 2017). This framework remains widely used to communicate findings and develop new methods and tools tailored to clinical applications (Bot et al. 2018; Caire et al. 2013; Chakravarty et al. 2006; Duchin et al. 2018; Hyam et al. 2015).

Successful AC–PC realignment depends on two essential steps. The first is midsagittal realignment, which adjusts the brain along the midsagittal plane to ensure symmetry and proper anatomical orientation. The second is AC–PC realignment, which aligns the desired AC–PC line with the brain’s anteroposterior axis (Lancaster et al. 1995). Both steps require precise definition of two key components: the desired AC–PC line and the midplane (Minoshima et al. 1994). Advances in image transformation techniques now allow the AC-PC line and midplane to be defined by placing points on relevant anatomical structures, such as the AC, the PC, and various midline structures. However, accurate identification of these landmarks requires considerable expertise in brain anatomy and MRI interpretation, and the process can be challenging in the absence of high-resolution images (Bhanu Prakash et al. 2006; Evans et al. 1992). The potential for discrepancies in landmark identification remains high, even among experts, underscoring the complexity of accurate AC-PC realignment (Pallavaram et al. 2008).

There have been few attempts to develop tools that can automatically realign the MR image to the Schaltenbrand AC-PC plane (Ardekani and Bachman 2009; Horn and Kuhn 2015; Horn et al. 2017; Oxenford et al. 2022). However, despite these developments, there is currently no clear guideline to validate the results of these automated methods or to realign the image manually in case automated methods fail. Hence a standard guideline on how to identify these anatomical structures on structural MR images for manual AC-PC realignment and validate the realignment is an important gap in the literature. In this study, therefore, we developed a protocol for the identification of the anatomical landmarks in T1-weighted MR data that are necessary for realigning structural MR images to the AC-PC plane, and for verifying the results of the ensuing AC-PC re-alignment. This proposed guideline has been developed to satisfy key features necessary for accurate data alignment:

1. The guideline adheres to the clinical version of the Schaltenbrand atlas (Fig. 1D), which is widely used in stereotactic and functional neurosurgery to localize subcortical structures (Horn et al. 2017). However, the guideline is still compatible with other AC-PC definitions, including the original AC-PC definitions by Talairach and Schaltenbrand (Fig. 1B and C).
2. The guideline does not assume prior anatomical knowledge nor experience with T1-weighted MR imaging and/or AC-PC realignment. These assumptions hold under extreme image rotations, across age groups, and for patients with Parkinson’s Disease.
3. The guideline was developed and validated on commonly used high-resolution structural contrast T1-weighted MR images but can also be used for other structural imaging contrasts such as T2-weighted MR image.
4. The guideline incorporates the results of a validation experiment involving both experts and non-experts.

## The guideline

The proposed protocol is structured into two sections. The first section provides a step-by-step guide for selecting the AC–PC points that define the AC–PC line, as well as the four midline landmarks required for defining the midplane. The second section outlines the visual criteria used to verify successful realignment to the AC–PC space. A comprehensive overview of the anatomical landmarks used in the guideline is provided in Supplementary Material 1: Anatomical Landmarks.

The protocol is intended for structural MR images in which the AC, PC, and midline structures can be identified with sufficient confidence. In cases where these landmarks are absent, severely distorted, displaced, or not clearly visible, such as in some pathological or congenital conditions, the applicability of the protocol may be limited, and expert anatomical judgment may be required.

### Protocol

Here, we present our protocol in a structured, pseudo-algorithmic format with step-by-step instructions.

#### Anterior commissure (AC)

**Fig. 2:**
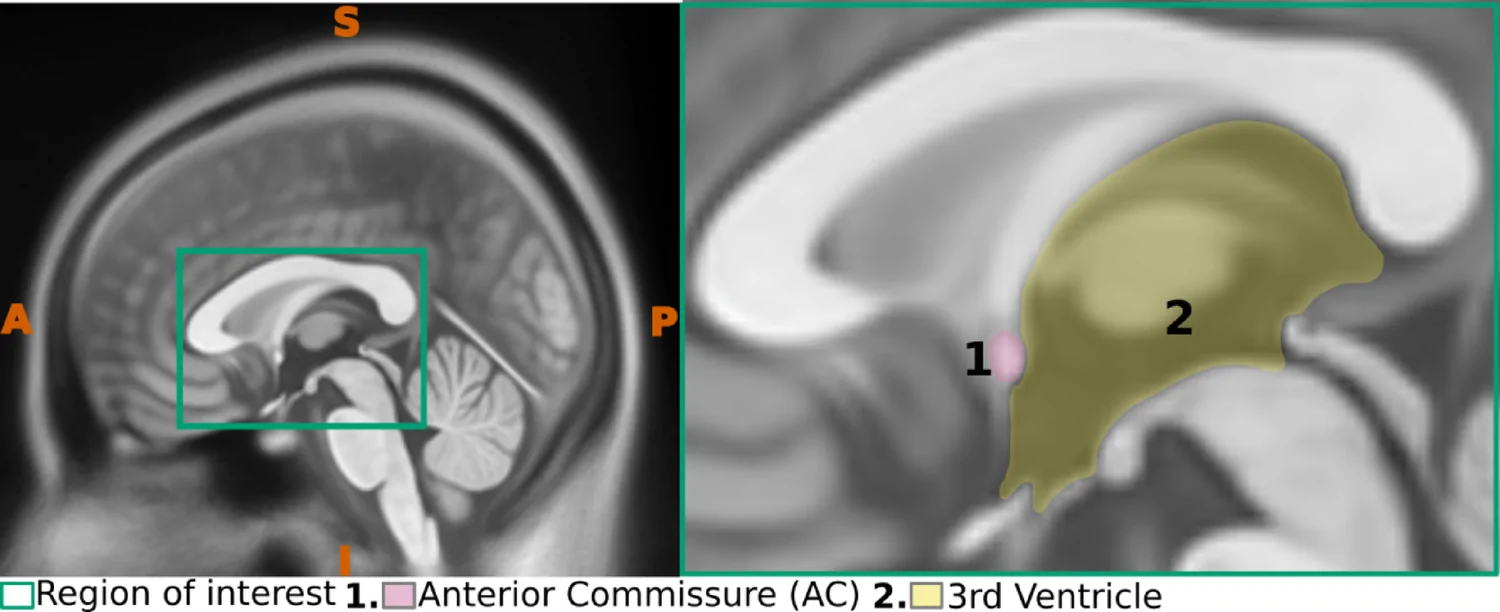
Sagittal view of the anterior commissure. The left panel presents a midsagittal view with the region of interest (ROI) highlighted, whereas the right panel offers an enlarged view of the ROI, emphasizing critical anatomical structures

**Fig. 3:**
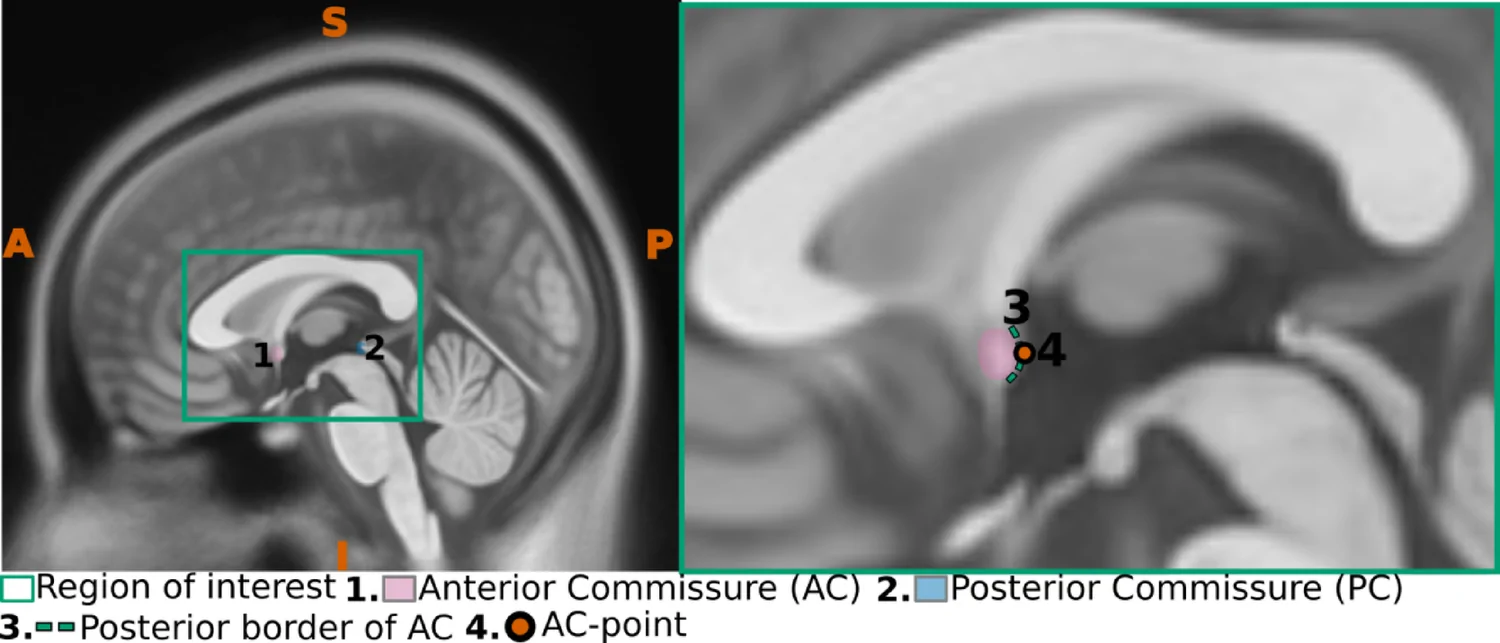
Sagittal view of the anterior commissure. The left panel presents a midsagittal view with the region of interest (ROI) highlighted, whereas the right panel offers an enlarged view of the ROI, emphasizing critical anatomical structures

**Fig. 4:**
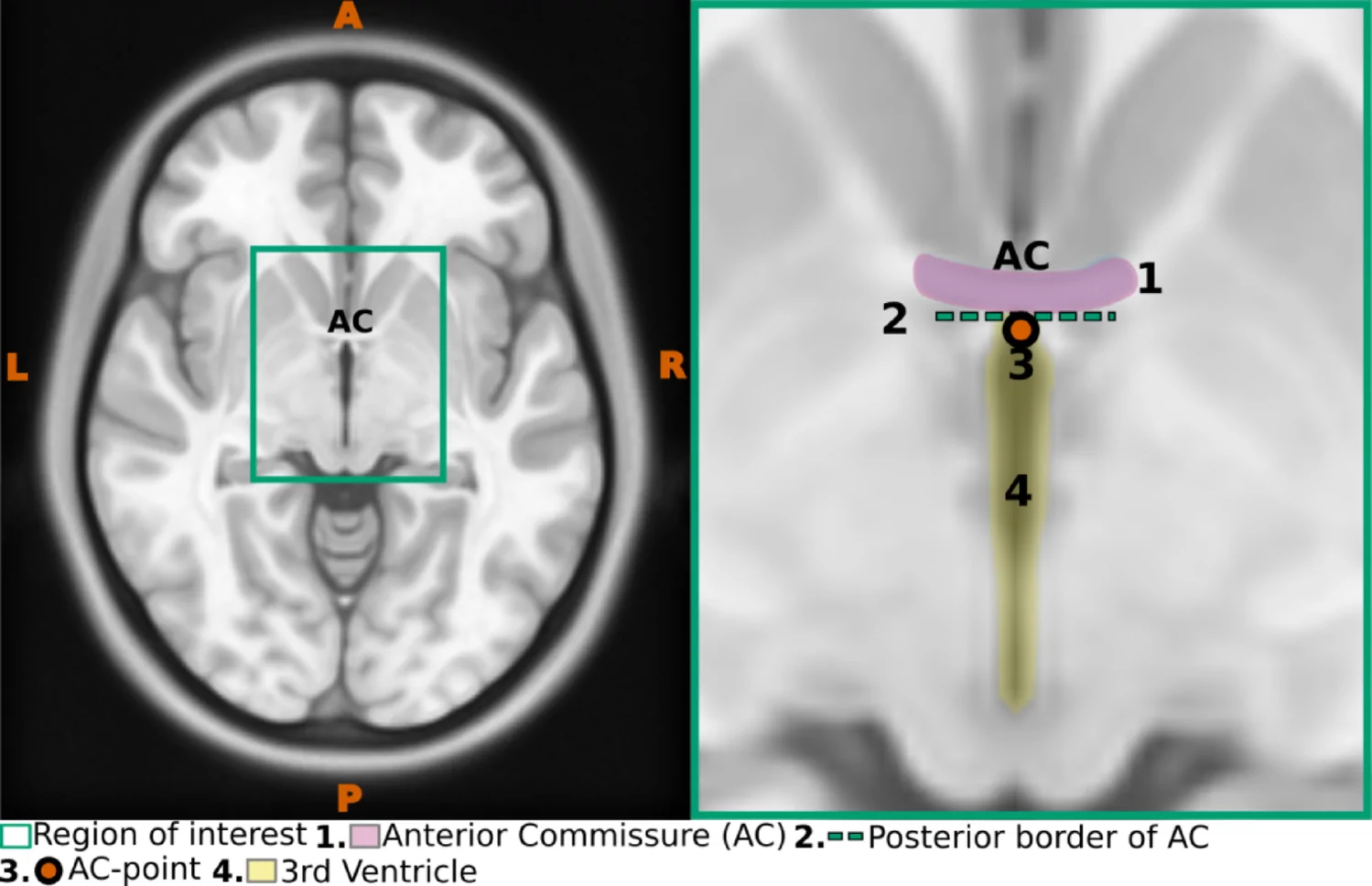
Axial view of the anterior commissure. the left panel presents an axial view with the region of interest (ROI) highlighted, whereas the right panel offers an enlarged view of the ROI, emphasizing critical anatomical structures

1. Identify the anterior commissure (AC; Fig. 2)

2. Select a point at the posterior border of the AC (Fig. 3)

3. Use the corresponding axial plane to ensure the annotated point is accurately positioned along the midline (Fig. 4).

#### Posterior commissure (PC)

**Fig. 5:**
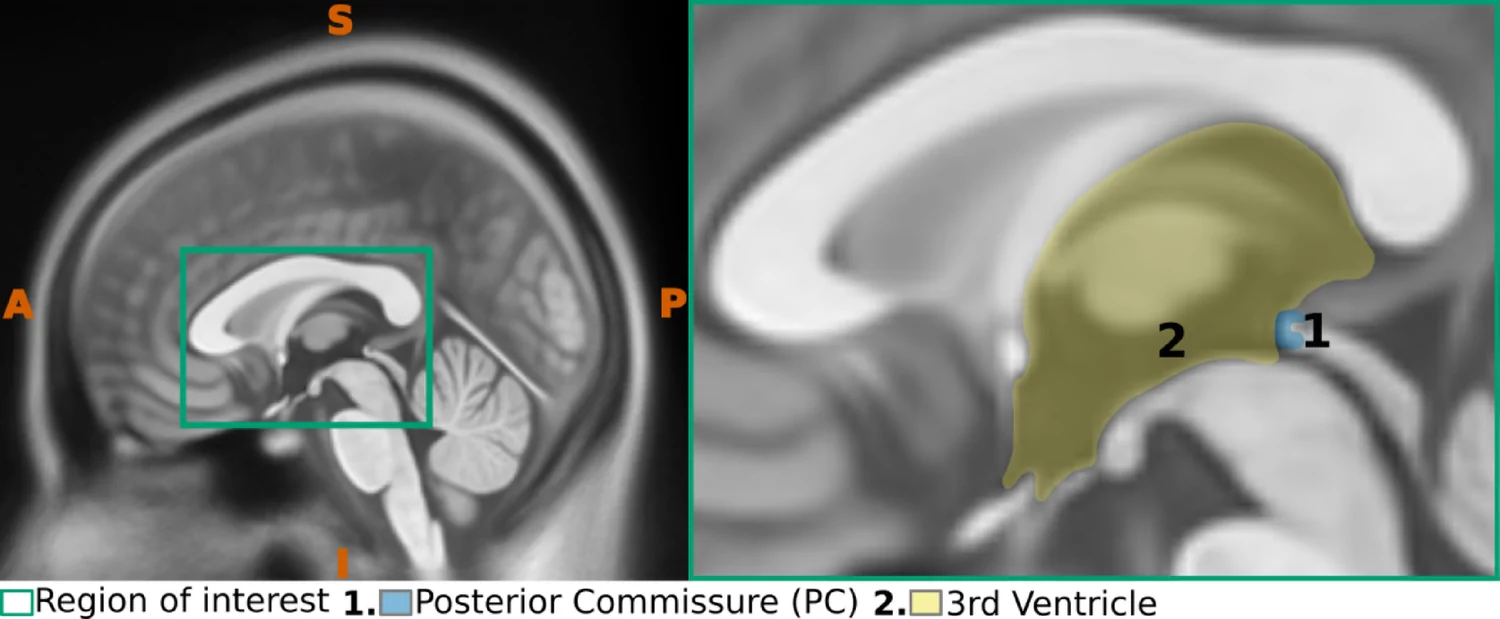
Sagittal view of the posterior commissure. the left panel presents a midsagittal view with the region of interest (ROI) highlighted, whereas the right panel offers an enlarged view of the ROI, emphasizing critical anatomical structures

**Fig. 6:**
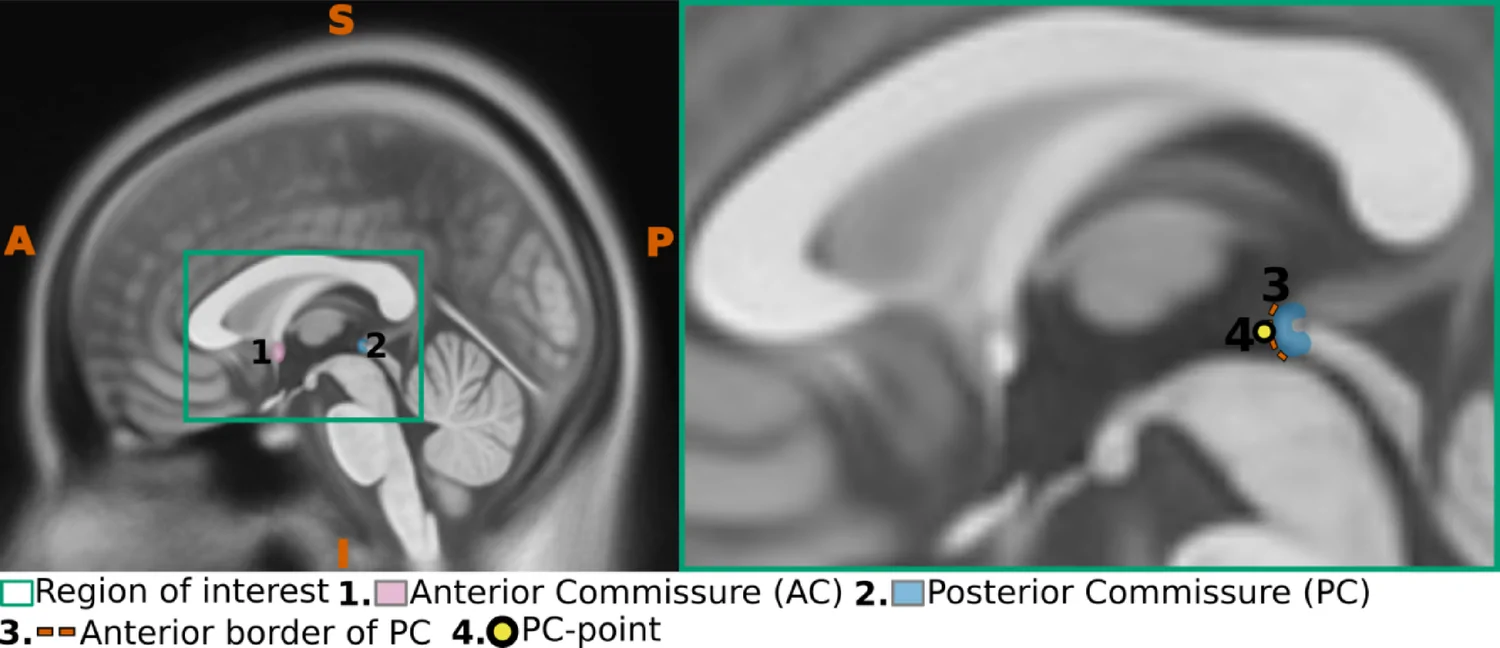
Sagittal view of the Posterior commissure. the left panel presents a midsagittal view with the region of interest (ROI) highlighted, whereas the right panel offers an enlarged view of the ROI, emphasizing critical anatomical structures

**Fig. 7:**
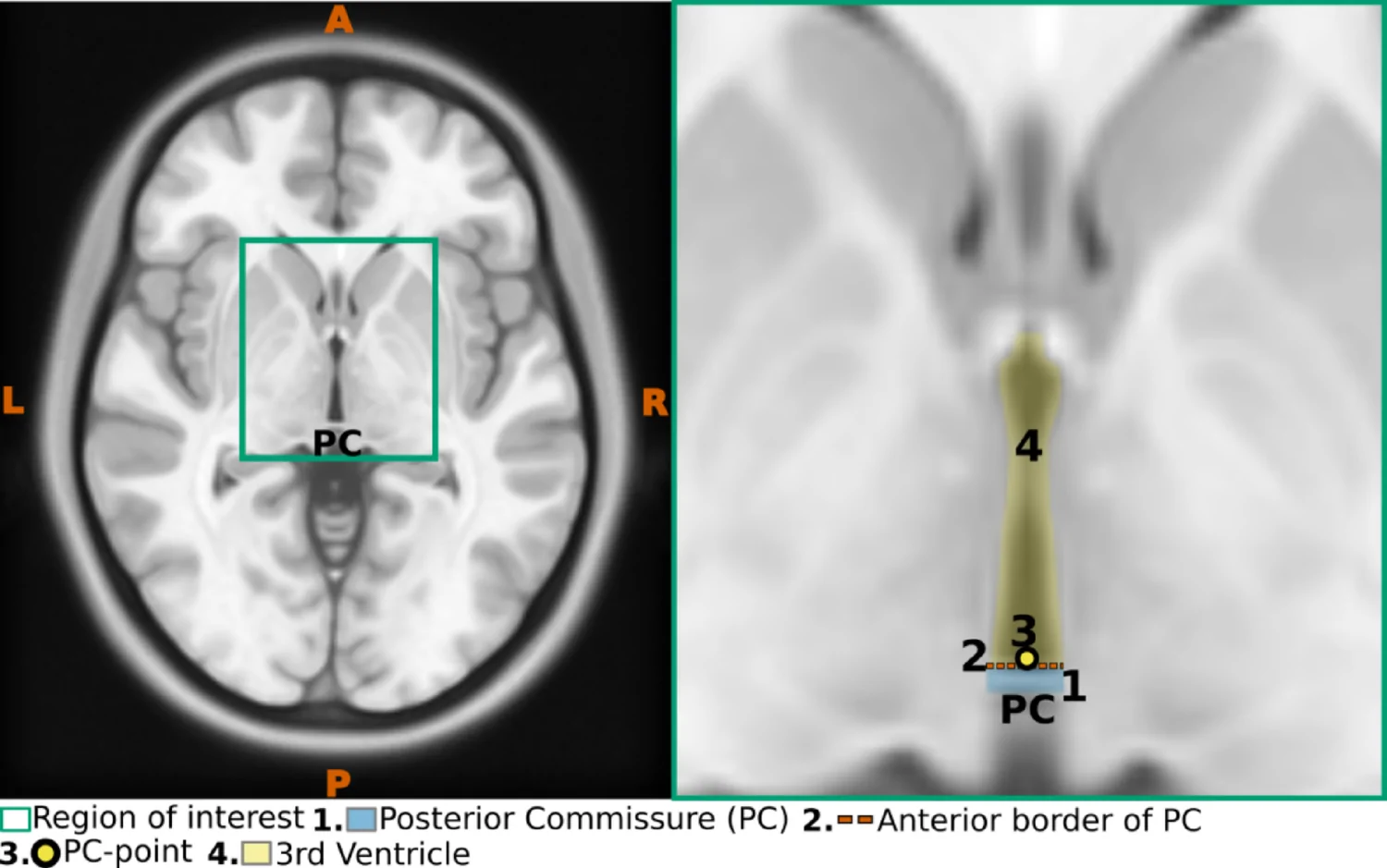
Axial view of the Posterior commissure. the left panel presents an axial view with the region of interest (ROI) highlighted, whereas the right panel offers an enlarged view of the ROI, emphasizing critical anatomical structures

1. Identify the Posterior commissure (PC; Fig. 5)

2. Select a point at the anterior border of the PC (Fig. 6)

3. Use the corresponding axial plane to ensure the annotated point is accurately positioned along the midline (Fig. 7).

#### AC-PC line

**Fig. 8:**
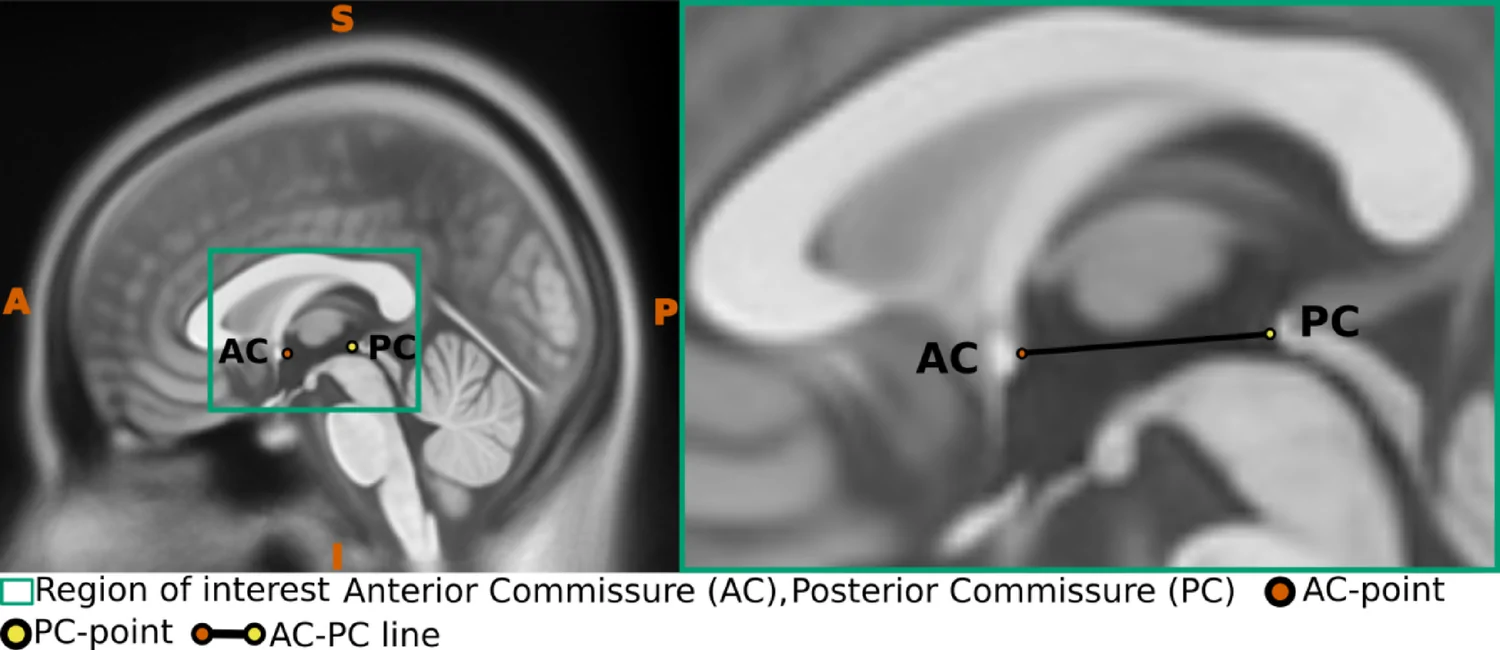
Sagittal view illustrating the AC-PC line. the left panel shows a midsagittal perspective with the region of interest (ROI) highlighted, while the right panel provides an enlarged view of the ROI, clearly emphasizing the AC-PC line

1. Ensure alignment along the AC-PC line, a straight line connecting the posterior border of the AC to the anterior border of the PC. This line should pass through the midpoints of both the AC–PC in sagittal orientation. Accurate definition of this line is essential for precise AC-PC realignment (Fig. 8).

#### Superior pontine notch

**Fig. 9:**
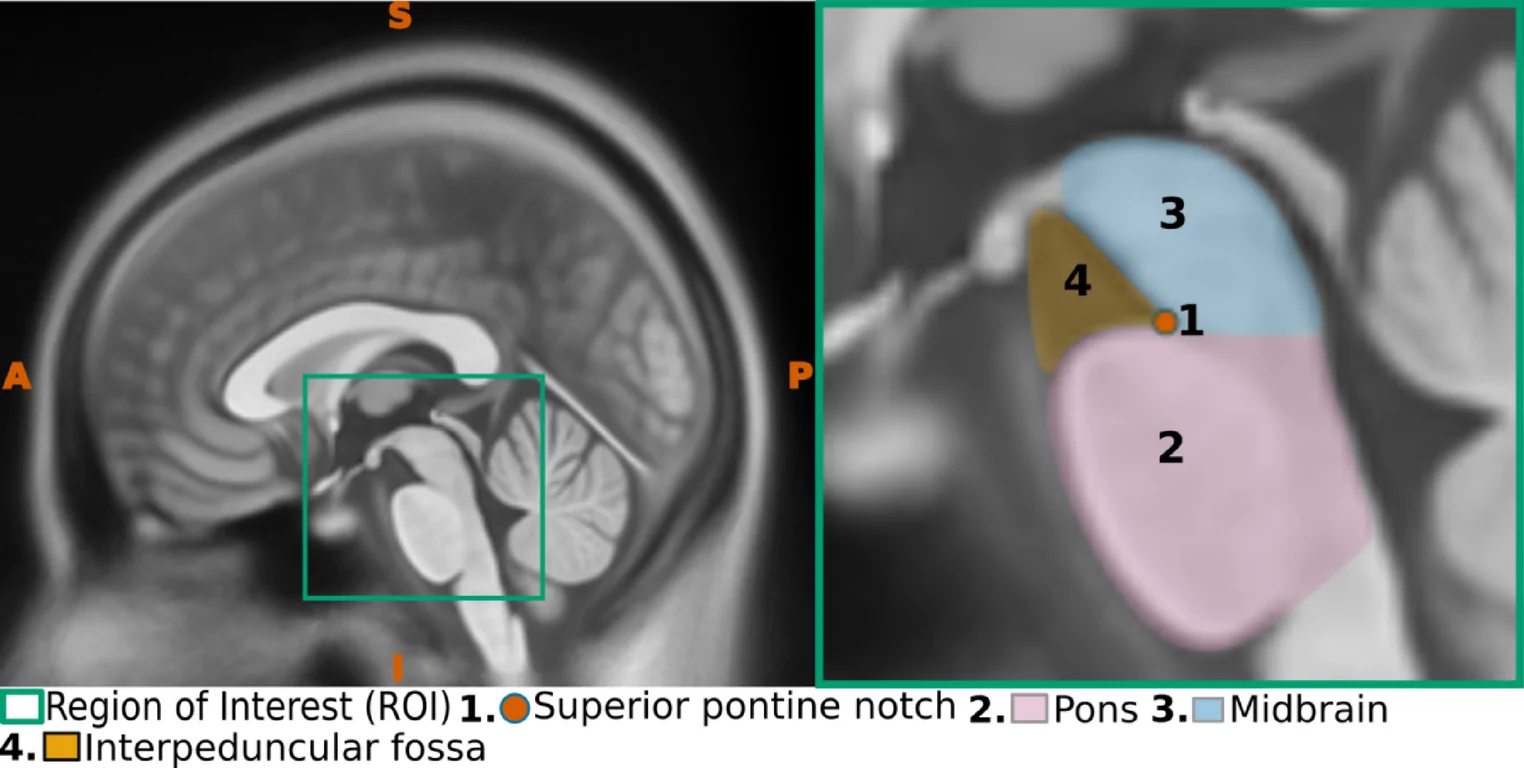
Sagittal view demonstrating the superior pontine notch. the left panel depicts a midsagittal perspective highlighting the region of interest (ROI), while the right panel provides a magnified view of the ROI, clearly emphasizing critical anatomical structures and specifically highlighting the superior pontine notch

**Fig. 10:**
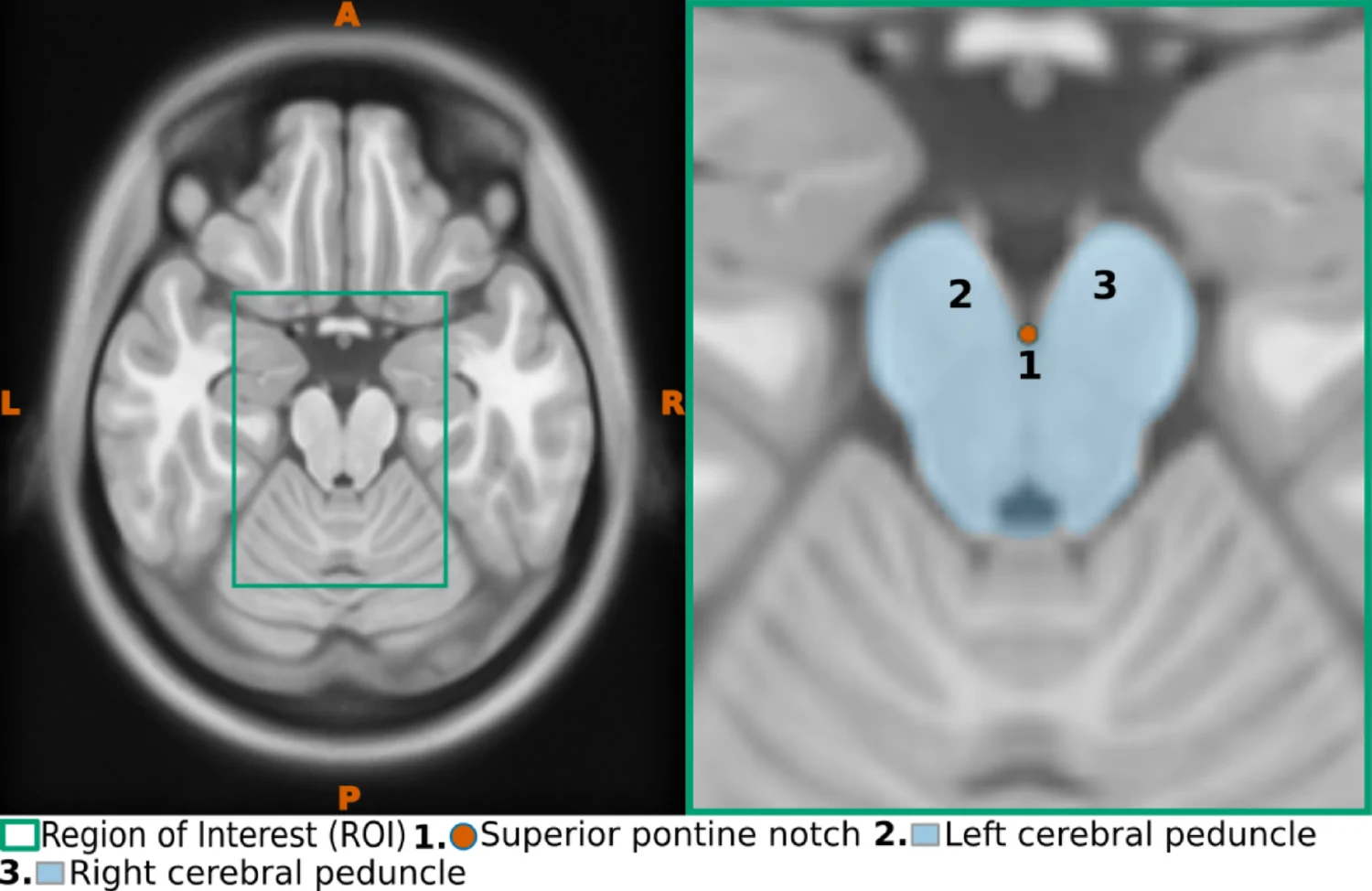
Axial view demonstrating the superior pontine notch. the left panel depicts an axial perspective highlighting the region of interest (ROI), while the right panel provides a magnified view of the ROI, clearly emphasizing critical anatomical structures and specifically highlighting the superior pontine notch

1. Identify the superior pontine notch (Fig. 9).
2. Place the first midline point in the superior pontine notch at the junction where the midbrain and pons meet (Fig. 9).

3. Use the corresponding axial slice to ensure the point is correctly positioned in the midline, where the two peduncles join to form the interpeduncular angle (Fig. 10).

#### Fastigium or apex of the roof of fourth ventricle

**Fig. 11:**
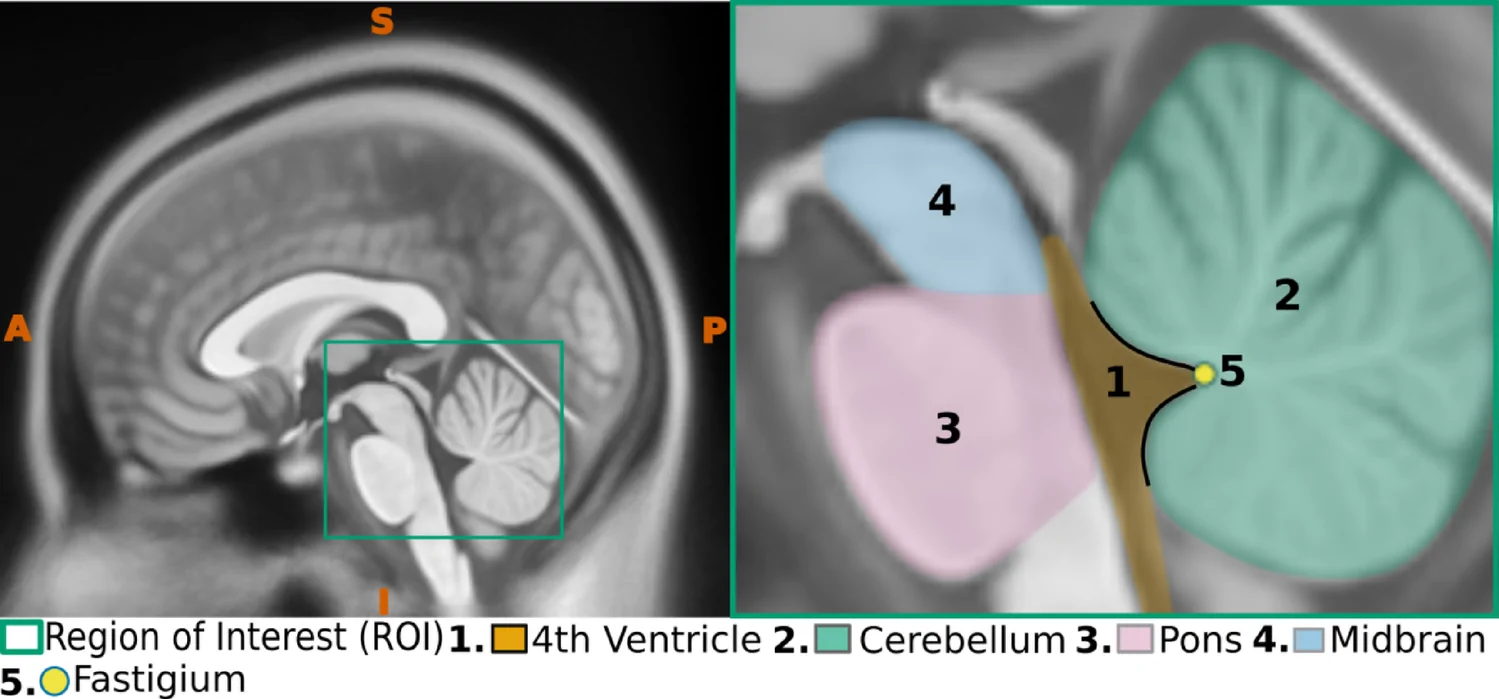
Sagittal view demonstrating the fastigium. the left panel depicts a midsagittal perspective highlighting the region of interest (ROI), while the right panel provides a magnified view of the ROI, clearly emphasizing critical anatomical structures and specifically highlighting the fastigium

**Fig. 12:**
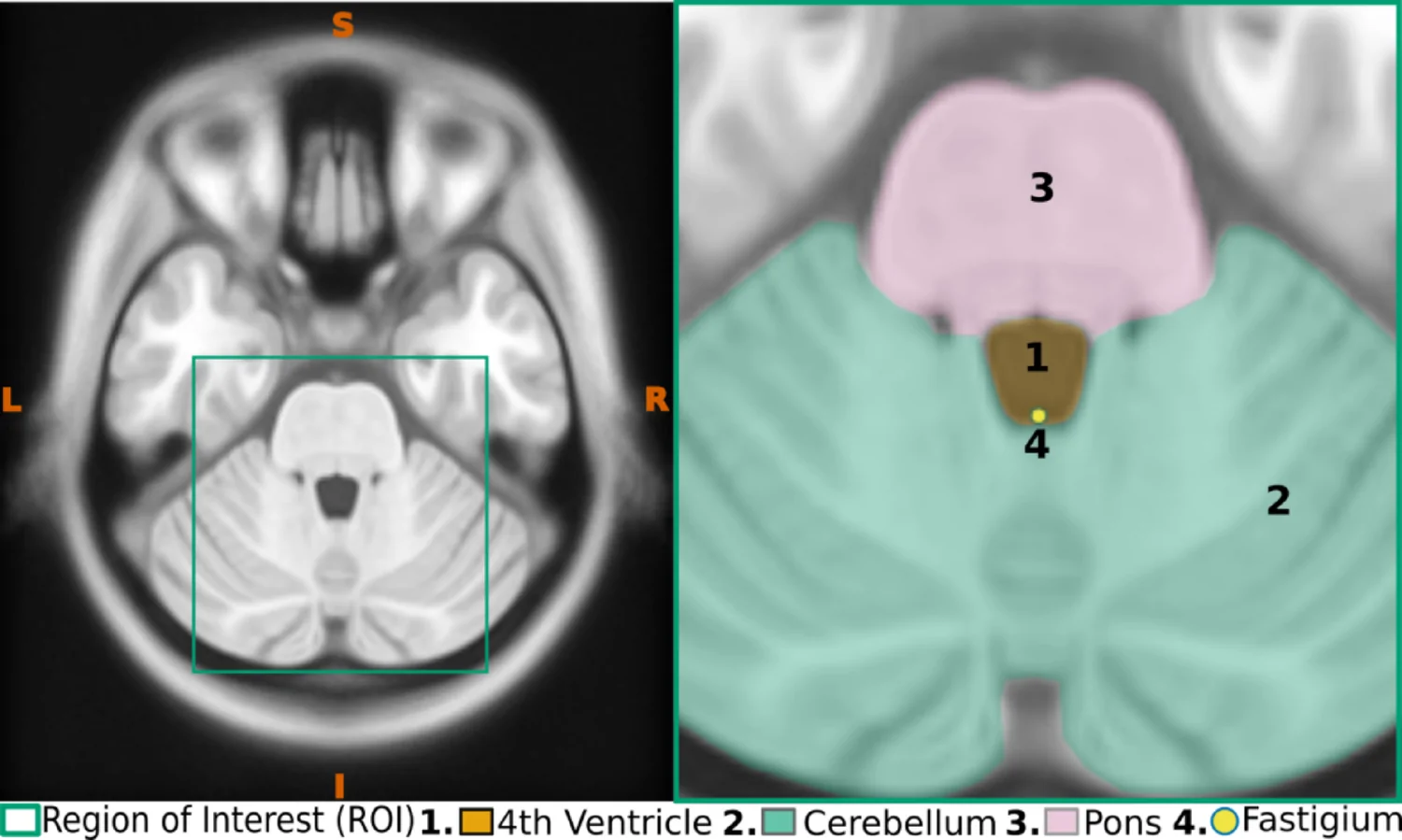
Axial view demonstrating the fastigium. the left panel depicts an axial perspective highlighting the region of interest (ROI), while the right panel provides a magnified view of the ROI, clearly emphasizing critical anatomical structures and specifically highlighting the fastigium

1. Identify the fastigium, also known as the apex of the roof of the fourth ventricle (Fig. 11).
2. Place the second midline point in the fastigium (Fig. 11).

3. Use the corresponding axial slice to ensure the point is accurately placed along the midline in the fastigium (Fig. 12).

#### Anterior and posterior points in the falx cerebri

**Fig. 13:**
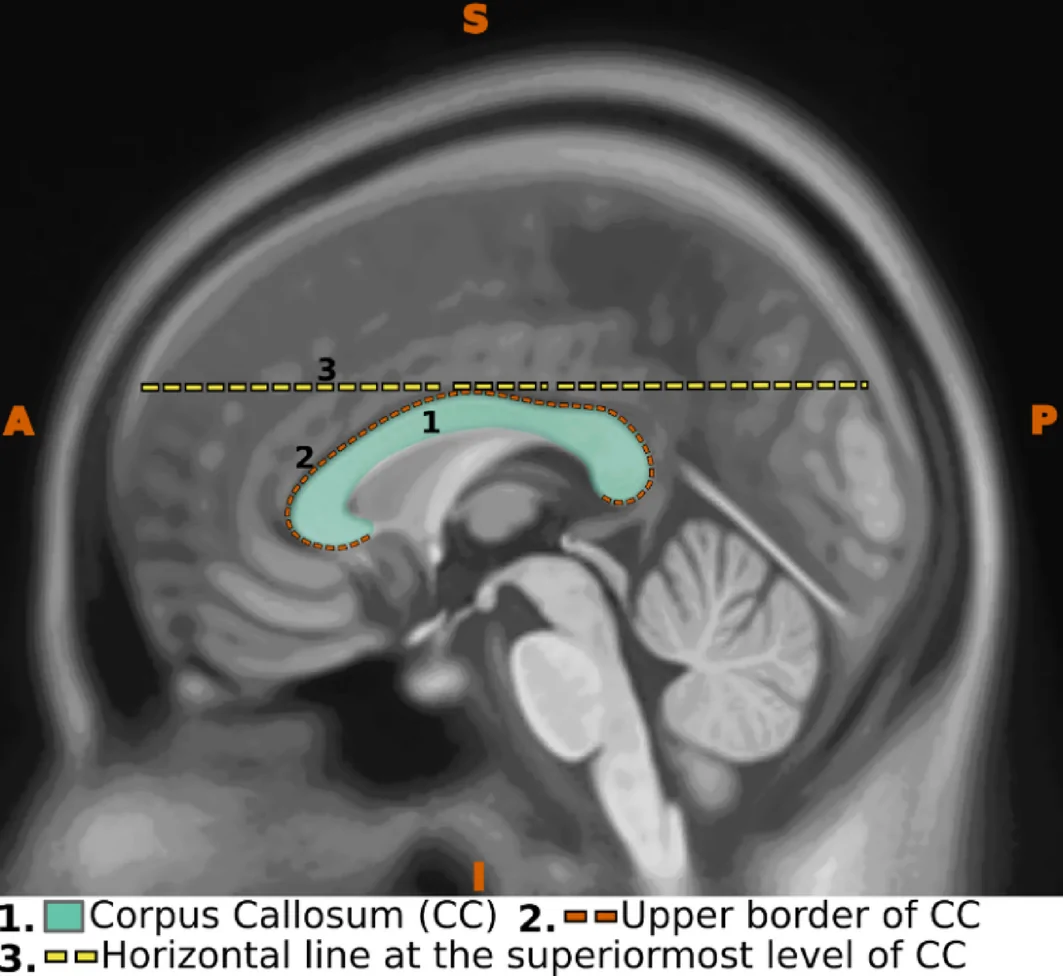
Sagittal view illustrating the corpus callosum with a horizontal line marking its superior-most boundary

**Fig. 14:**
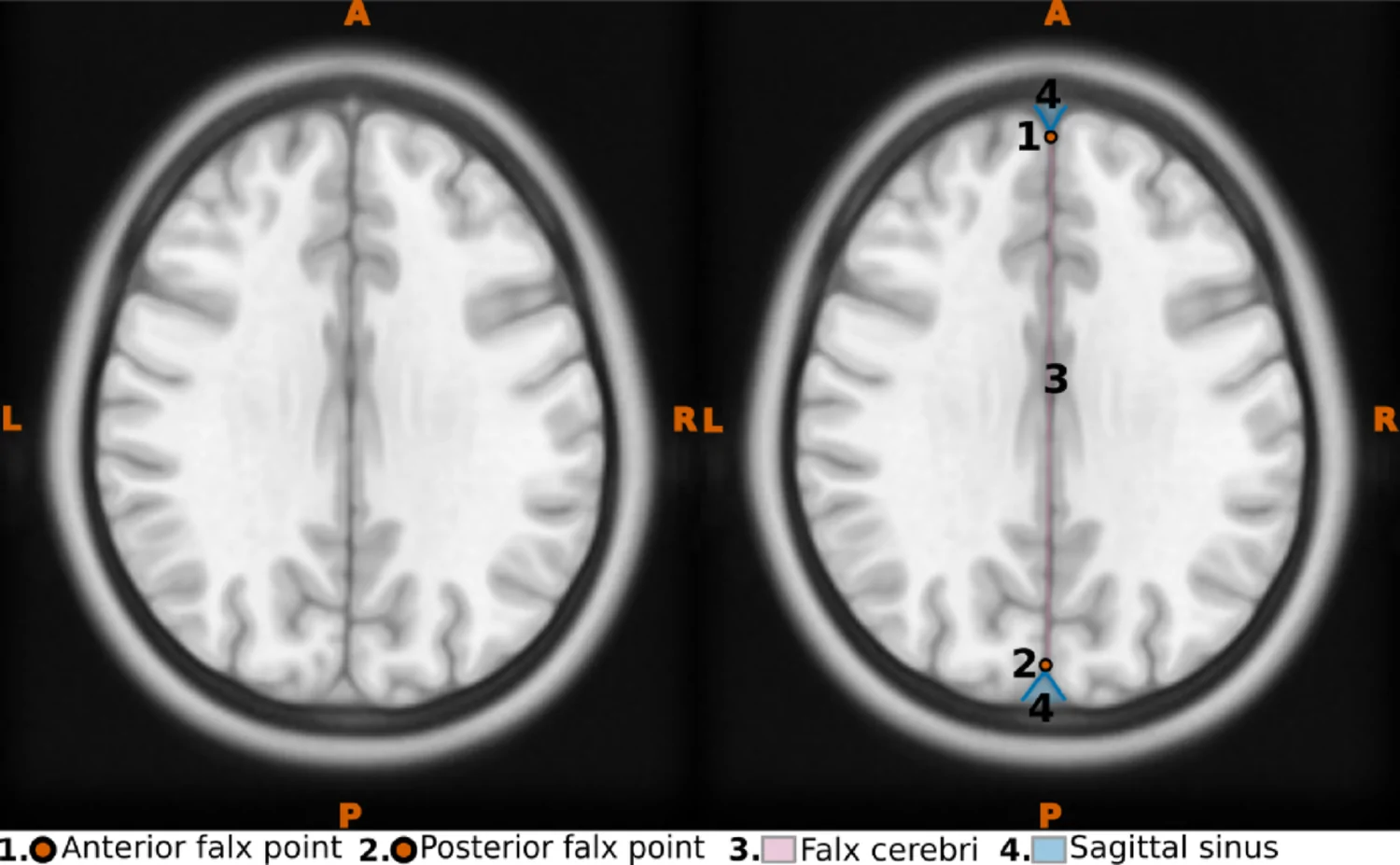
Axial view illustrating the falx cerebri at the midline. The left panel displays an axial perspective at the superior-most level of the corpus callosum, whereas the right panel highlights the falx cerebri and other key anatomical landmarks

1. Identify the corpus callosum (Fig. 13).
2. To mark its superior-most boundary, draw a line along the upper border of the corpus callosum, ensuring it is aligned with the highest point of this structure (Fig. 13). In conditions of minimal rotation, this should ideally correspond to the central body portion of the corpus callosum where it arches. However, the exact positioning may vary depending on the degree of rotation applied to the image. To maintain proper correspondence of the midpoint 3 and 4, it is essential to verify that this point corresponds to the superior-most section of the corpus callosum in a sagittal view.

3. Use the corresponding axial plane to place two midline points (the 3rd and 4th points; Fig. 14): one at the anterior end of the falx cerebri and another at the posterior end. Ensure to place the points at the junction between the falx cerebri and the superior sagittal sinus. This junction can be identified as the apex of the triangular appearance of the superior sagittal sinus in a transverse section (refer to Fig. 14).

### Verification of AC-PC alignment

After realigning the structural image to the AC-PC plane, it is essential to verify that the image is properly aligned. This involves checking for any rotations in the X, Y, and Z directions, commonly referred to as pitch, roll, and yaw.

#### Verification of pitch

**Fig. 15:**
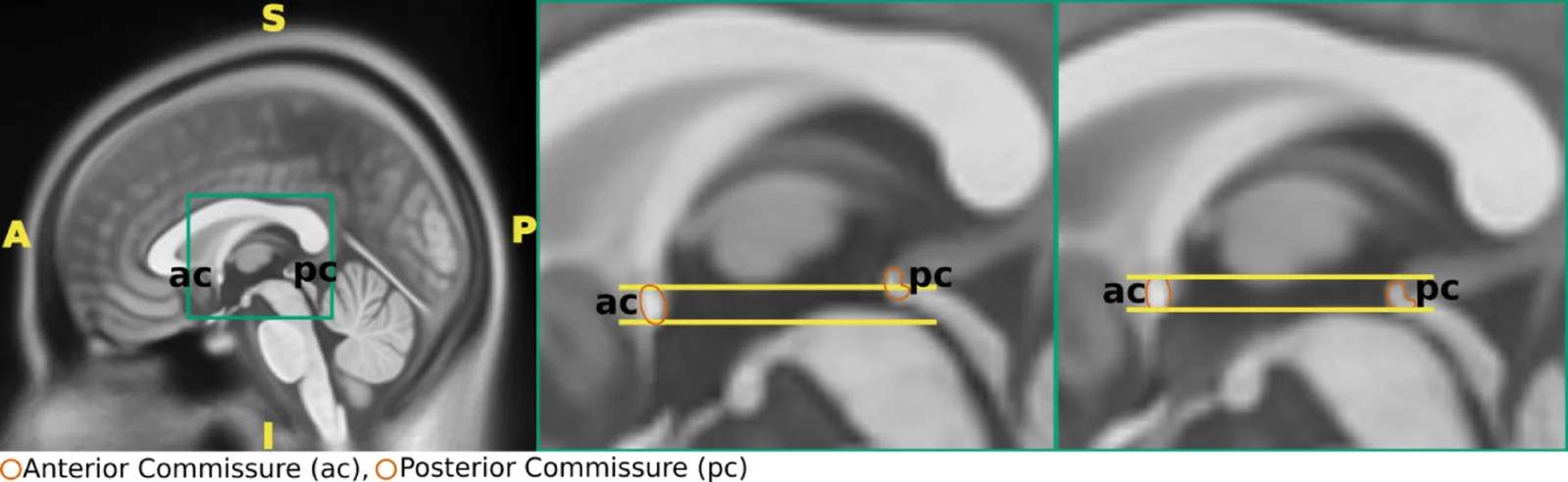
Verification of pitch. from left to right, the first panel shows a midsagittal plane highlighting the region of interest (ROI). The second and third panels provide enlarged views of the ROI: the second panel illustrates the positions of the AC–PC before realignment, whereas the third panel displays their positions after realignment

**Fig. 16:**
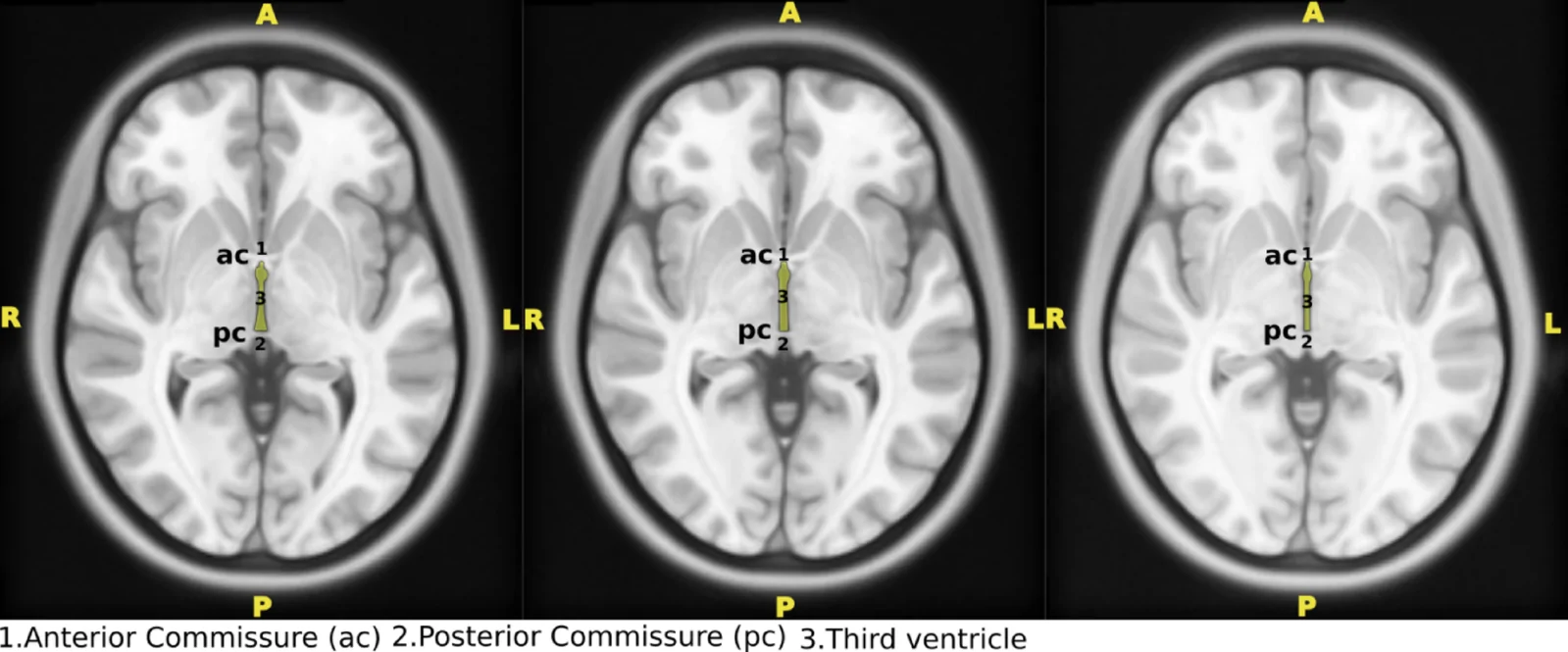
Verification of pitch. axial planes demonstrating criteria for verifying rotation around the Y-axis. Note the appearance of AC–PC in consecutive axial slices, as well as the characteristic keyhole shape of the third ventricle in the first two slices

1. After successful realignment, the AC and the PC should be aligned on the same horizontal level. To verify this, draw two parallel lines along the upper and lower borders of the AC in the midsagittal plane. The space between these lines should encompass both the AC – PC, confirming their alignment (Fig. 15).

2. By examining the corresponding axial plane, the AC–PC should be visible within the same slice. For high-resolution images (with 1 mm or smaller isotropic voxel size), both the AC–PC should appear across three consecutive slices. The presence of the AC and the PC in the same axial plane slice will create the characteristic keyhole appearance of the third ventricle (Fig. 16).

####  Verification of roll

**Fig. 17:**
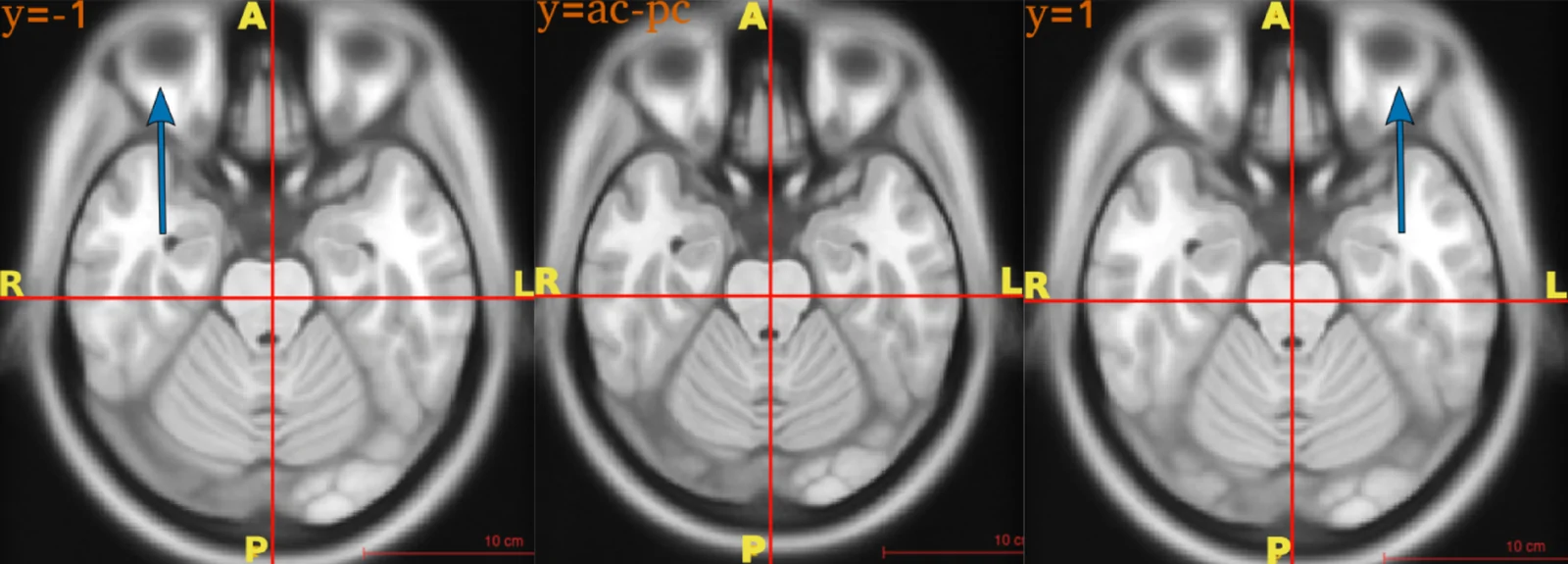
Verification of roll. axial planes illustrating the appearance of the eyeballs in three scenarios. From left to right: the first panel shows the right eyeball appearing larger than the left; the middle panel depicts both eyeballs equal in size; and the rightmost panel illustrates the left eyeball appearing larger. Note the subtlety of these differences

**Fig. 18:**
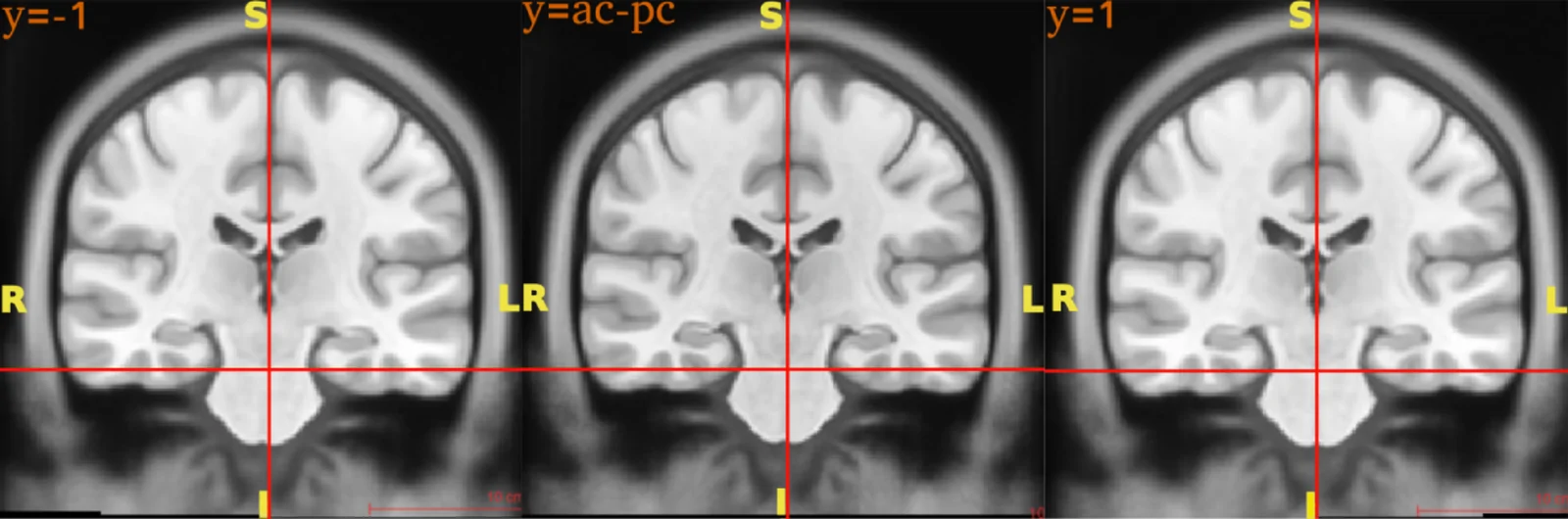
Verification of roll. coronal planes demonstrating midline deviations in three cases, marked by a vertical crosshair. From left to right: the first panel illustrates a deviation to the right, the middle panel shows proper midline alignment (no deviation), and the right panel reveals a deviation to the left. Note that these deviations are subtle, making them challenging to visually distinguish

1. To verify the roll alignment, examine the position of the eyeballs in the selected axial slice; they should appear at the same level (Fig. 17). In cases where minor deviations are difficult to discern visually, coronal planes can provide an additional perspective to confirm precise AC-PC realignment (Fig. 18).

#### Verification of yaw

**Fig. 19:**
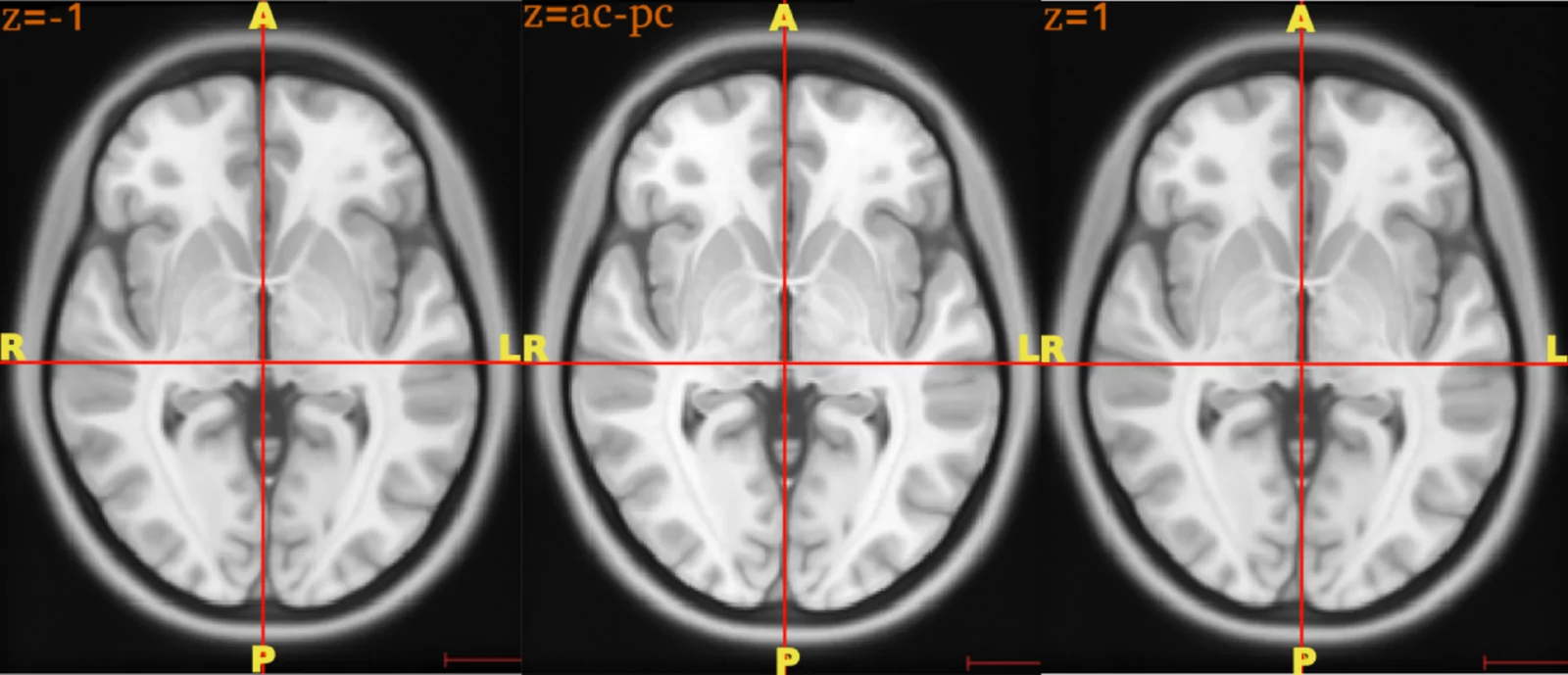
Verification of yaw. axial planes illustrating midline deviations in three cases, as indicated by the vertical crosshair. from left to right, the first panel illustrates a deviation to the left, the middle panel shows proper midline alignment (no deviation), and the right panel reveals a deviation to the right

1. The yaw could be verified by placing the vertical crosshair. The vertical line should align with the longitudinal fissure or midline of the brain in the axial plane (Fig. 19).

##  Justification

Achieving precise AC–PC alignment depends on two critical factors: (1) an accurately defined AC–PC line and (2) a clearly established midplane. These elements form the foundation of our approach in developing this guideline, with each design choice was based on careful consideration of anatomical landmarks that are consistently visible on structural MR images and can be identified using standardized visual criteria. In addition, these choices were refined through experimental procedures and feedback from the six manual observers who participated in the validation experiment.

### Design choices

####  Definition of the AC and PC line

We adopted the clinically used definition of the AC-PC line (Fig. 1 d), which is defined from the posterior border of the AC to the anterior border of the PC (Acar et al. 2007; Caire et al. 2009; Horn et al. 2017; Patel et al. 2008; Saint-Cyr et al. 2002; Schuurman et al. 1999). The Schaltenbrand atlas remains a cornerstone reference in modern stereotactic neurosurgery (Gildenberg et al. 2009; Pallavaram et al. 2009). However, there is notable variation between the AC–PC line definition used in clinical practice and that described in the original atlas. (Horn et al. 2017) (Fig. 1C-D). This discrepancy arises from the necessity to adapt a post-mortem atlas for in vivo imaging, a challenge that was particularly prominent before the advent of MRI technology (e.g. for cerebral ventriculography images). Despite advancements in imaging, this adaptation persists in current surgical practices (Lau 2022; Pallavaram et al. 2009).

Other definitions, such as the tangential inter-commissural line used in the Talairach atlas (Fig. 1B), are influenced by the size and shape of the AC–PC. These factors can substantially affect the angle of the AC–PC line (Choi et al. 2013; Nowinski 2001). Given that the AC–PC can vary in shape, ranging from round to elliptical, defining the AC-PC line based on the superior and inferior edges becomes unreliable and prone to errors (Park et al. 2010). This challenge is further compounded by poor image resolution, which can make accurate landmark identification more difficult (Choi et al. 2013).

Beyond its status as a surgical convention, defining the AC–PC line from the posterior border of the AC to the anterior border of the PC offers several practical advantages. First, identifying the borders of the AC–PC is more straightforward, as this can be accomplished by tracing the boundaries of the third ventricle, which is often easier than locating their centers or superior and inferior edges. Second, defining the line from the borders of the AC–PC remains consistent across different imaging modalities and various populations (e.g. healthy vs. atrophied). Moreover, the borders are less affected by variation in the size and shape of the AC–PC (Choi et al. 2013). Third, reliable identification of the borders of the AC–PC does not require high-resolution images or explicit sagittal slices (Otake et al. 2018). Unlike the centers and superior and inferior edges of the AC–PC, the borders can be identified in axial slices and low-resolution images (Choi et al. 2013; Sandor et al. 1994).

####  Selection of the midline-points

**Fig. 20:**
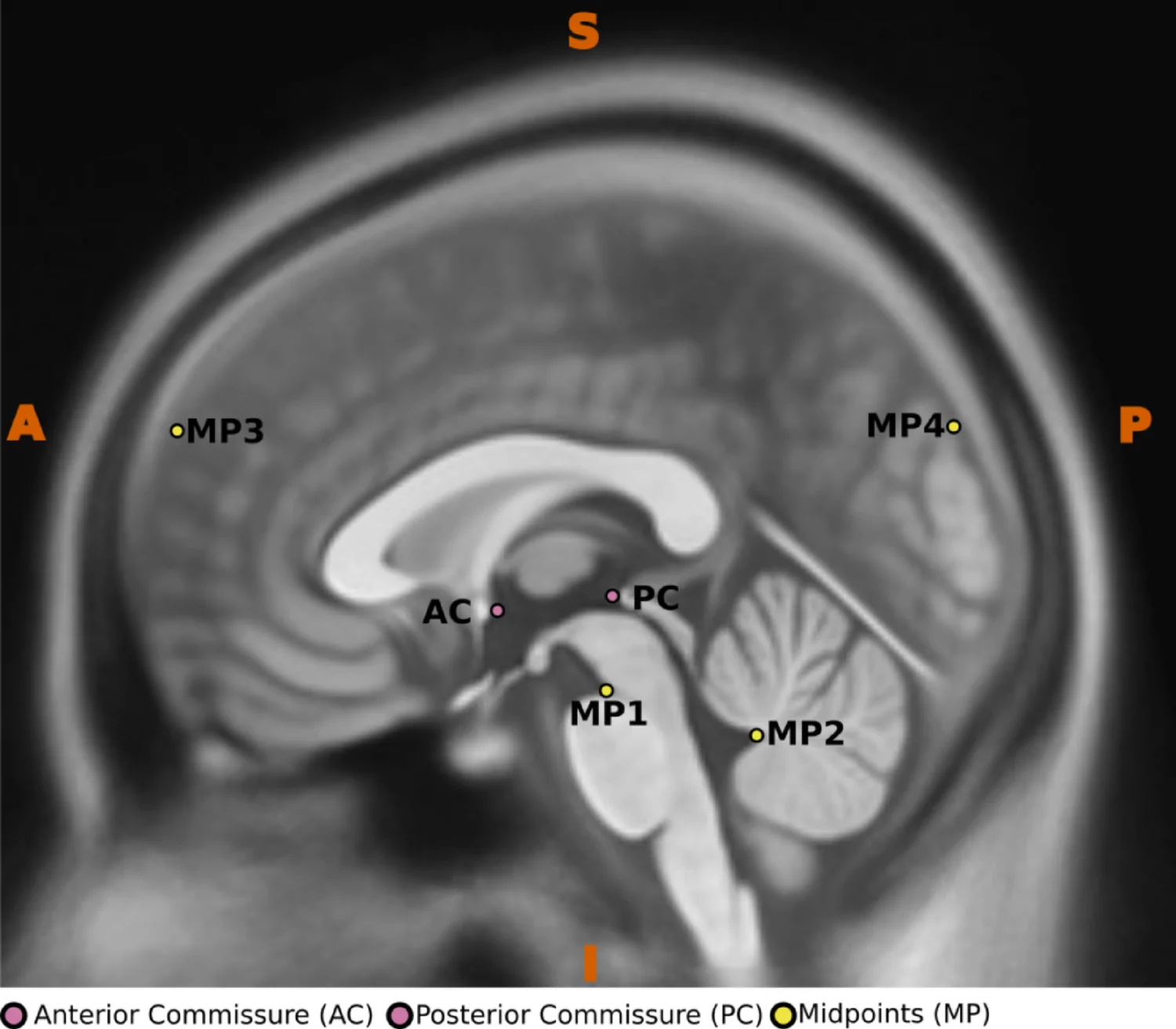
Sagittal view illustrating all landmark points, including the anterior commissure (AC), posterior commissure (PC), and the four midline reference points (MP)

To robustly define the midplane, at least three non-collinear points are required. In the proposed protocol, we have introduced four midline points to facilitate midplane definition (Fig. 20). The four proposed midline points are:

(1) the superior pontine notch

(2) the fastigium of the fourth ventricle

(3) the anterior point in the falx cerebri

(4) the posterior point in the falx cerebri

The selection of these four specific landmarks is based on their extensive use across both clinical and research contexts including ac-pc realignment, which establishes them as highly validated reference structures.(Ardekani and Bachman 2009; Chen et al. 2010; Horn et al. 2017; Liu and Dawant 2015; Niemann et al. 1999; Oba et al. 2005; Otake et al. 2018; Zrinzo et al. 2008). Their consistency, reliability, and relative ease of identification across imaging modalities, whether assessed manually or using automated computer vision techniques, further enhance their versatility and make them valuable landmarks for a broad range of applications (Ardekani & Alzheimer’s Disease Neuroimaging, 2022; Lau et al. 2019; Roelants et al. 2016; Shah et al. 2013).

These landmarks also provide broad anatomical coverage. Two are positioned at the supratentorial level, specifically along the falx cerebri, while two are located at the infratentorial level, namely the superior pontine notch and the fastigium of the fourth ventricle. This distribution helps ensure balanced representation of the brain’s anatomy and reduces the potential influence of localized anatomical or imaging anomalies (Liu et al. 2001; Shah et al. 2013; Yap et al. 2023).

In addition, we propose defining two distant points along the same anatomical line to minimize error during plane fitting and to span a longer plane (Sandor et al. 1994). This approach is particularly useful when using algorithms such as principal component analysis (PCA) to fit a plane. For example, the AC–PC transformation model in 3D Slicer uses a PCA-based approach for accurate plane fitting.

#### Selection of verification criteria

As the final step in our protocol, we propose three visualization criteria to verify successful AC-PC realignment. Clinically, AC-PC realignment requires rotating the image around the three cardinal planes (x, y, z) to align the AC-PC at the same level within the horizontal plane(Caire et al. 2011; Saint-Cyr et al. 2002). Consequently, the verification criteria for successful AC-PC realignment are specifically designed to ensure the accuracy of rotations around the X, Y, and Z axes.

Among these criteria, verifying the pitch or X-axis rotation is the most critical, as it confirms that the image is properly aligned with the clinically utilized AC-PC plane (Hamilton et al. 2017; Lancaster et al. 1995; Ramirez et al. 2014). Since both the AC and the PC can be clearly visualized in the mid-sagittal and axial planes, we utilized both planes to verify the level of AC–PC to ensure precise X-axis rotation. It is important to note that the criteria to verify pitch or X-axis rotation is specifically applicable to verify the Schaltenbrand definition of the AC-PC plane (Horn et al. 2017), encompassing both the clinical and original versions. To verify the roll or Y-axis rotation, we utilized both the axial and coronal planes. In the axial plane, we focused on aligning the level of the eyeballs, while in the coronal plane, we emphasized ensuring midline alignment (Hamilton et al. 2017; Lancaster et al. 1995; Ramirez et al. 2014). Finally, to verify the yaw or Z-axis rotation, we employed the axial plane to assess midline alignment (Hamilton et al. 2017; Lancaster et al. 1995; Ramirez et al. 2014). The criteria addressing roll (Y-axis rotation) and yaw (Z-axis rotation) are essential for maintaining proper alignment and symmetrical positioning of the brain. Consequently, these two criteria can be used to verify all definitions of AC-PC plane alignment, including those established by Talairach.

## Guideline validation methodology

###  Data preparation and pre-processing

For validation of the first and second stages, we used three structural T1-weighted images from two different datasets. Two images were randomly selected from the open-source ABRIM dataset (Jansen et al. 2024) one from a healthy young adult participant aged 18–35 years and one from a healthy older adult participant aged 60–75 years. In addition, one representative T1-weighted image from a patient with Parkinson’s disease was randomly selected from the POM dataset.

The three selected images were cropped to remove the large neck region using a robust field of view (FOV) method, and subsequently underwent bias correction (Zhang et al. 2001) utilizing the fsl_anat tool from the FSL software package (Jenkinson et al. 2012; Tian et al. 2016). Following this, the bias-corrected images were aligned to the AC-PC plane using the precursor guideline by the first author (AS). Once aligned, these images were rotated to three different angles. These rotation angles were determined from 226 ABRIM subjects, using the linear registration matrix associated with those subject’s transformation to the MNI 152 space. Among the 266 rotations, three key values minimum, mean, and maximum were selected for image rotation. These angles were then randomly sign-flipped, resulting in the minimum value shifting to the mean position and the mean value shifting to the minimum. The resulting rotations, Rmin, Ravg, and Rmax, were then used to generate rotated images with known rotation parameters (Tables 1 A and B). The rotated images were then subjected to intensity normalization using the percentile clipping method (i.e. removing the bottom and top 2%) see supplementary Figure.24 for the full data preparation pipeline.

For the inter- and intra-rater reliability assessment, 12 additional structural T1-weighted images were randomly selected from the open-source ABRIM dataset (Jansen et al. 2024). The selected participants ranged in age from 18 to 75 years. These images were cropped, bias-field corrected, and intensity-normalized using the same methods described above. Unlike the three images used for the rotation-based validation, these 12 images were not AC-PC aligned or subsequently rotated. Instead, they were used specifically to assess the influence of the proposed guideline on landmark identification.

### Experimental procedures

The evaluation process comprised three stages. In the first stage, manual AC–PC realignment was performed by two expert and four non-expert volunteers using the precursor version of the guideline. The expert volunteers were practicing neurosurgeons who routinely perform manual AC–PC realignment in clinical practice. Each expert completed the realignment task twice: first without the guideline and then with the guideline. The non-expert volunteers had no prior anatomical knowledge or experience with AC–PC realignment and performed the task using the guideline only. In the second stage, AC–PC realignment was performed using four existing automated methods. The results from the first two stages, together with structured observer feedback, were used to refine the precursor guideline. In the third and final stage, the refined version of the protocol was evaluated, with a primary focus on assessing the influence of the guideline on landmark identification. For this stage, two additional non-expert volunteers performed the AC-PC alignment across two separate sessions. All manual realignments were performed using 3D Slicer version 5.0.3 (Fedorov et al. 2012).

To establish the reference baseline for the first two stages of the evaluation, the first author (AS), who was not involved as an evaluated observer in the experiments, manually realigned the three rotated images generated during data preparation to the AC–PC plane using the precursor guideline. Importantly, this reference baseline was generated from the same rotated images used in the evaluation, rather than being based solely on the initial pre-rotation alignment. This approach was adopted because different rotation angles may affect landmark visibility and thereby influence AC–PC realignment accuracy. Establishing the baseline under identical rotation and imaging conditions therefore provided a task-matched expert reference for comparison.

The spatial locations of the AC–PC borders, together with four midline points identified by the volunteers and automated methods, were compared with the reference baseline using Euclidean distance. In addition, image rotation angles and the root mean squared deviation (RMSD), derived from the rotation matrix, were compared against the baseline. Angular and spatial errors were computed using custom Python functions implemented with open-source python libraries. while RMSD was calculated according to the method described by Jenkinson (1999). Because the final stage was designed to assess inter- and intra-rater reliability in landmark identification, no reference baseline was required for this comparison. Instead, the spatial locations of the AC–PC borders and the four midline points identified by the two non-expert raters across two separate sessions were compared within and between raters using Euclidean distance differences and intraclass correlation coefficient metrics.

### Expert performance with and without the guideline

Two experts were tasked with realigning three rotated images (*R*_min_, *R*_avg_, and *R*_max_) to the AC-PC Plane. First, the experts realigned the images without the aid of our guideline. They adhered only to the minimal requirements of the AC-PC transformation module in 3D Slicer software, which involves selecting the AC–PC points along with at least three midline points to create an AC-PC transformation. Next, the experts performed the AC-PC realignment using the proposed guideline. To ensure a balanced assessment, the order of the rotation conditions was reversed between the experts. We evaluated the experts’ performance by measuring the RMSD, rotation angles, and Euclidean distances between the spatial locations of the AC, PC, and midline points relative to the baseline.

#### AC and PC location errors

Experts consistently demonstrated Euclidean distance errors of less than 1 mm across all rotation conditions, regardless of the presence of the guideline. Specifically, for *R*_min_, the differences in AC–PC showed errors below 0.6 mm, which is smaller than the voxel size (0.8 mm). Similarly, the AC–PC distance error also remained below 0.6 mm. The average diameters of the commissures (3 mm for the AC and 2 mm for the PC in the sagittal plane), and the average AC-PC distance (24 mm) as reported by Schaltenbrand (Schaltenbrand et al. 1977).

For *R*_avg_, experts achieved slightly better accuracy for the AC when using the guideline, whereas better results for the PC were observed without it. In both cases, the AC-PC distance error remained below 0.3 mm. Under the *R*_max_ condition, performance was generally better without the guideline, with the exception of a single PC instance where the error reached 0.87 mm. Overall, experts demonstrated consistently high accuracy across all conditions, with no significant differences in performance between the guideline and no-guideline scenarios. These findings suggest that the proposed guideline closely aligns with the AC-PC selection strategies employed by expert neurosurgeons, highlighting strong similarities between established clinical conventions and the guideline’s recommendations (Table 2).

**Table 2:** Comparison of errors in AC-PC Location and AC-PC Distance. This table illustrates the errors (in mm) in AC-PC location and AC-PC distance, comparing results between experts and baseline. the comparison is made both with and without the use of guidelines

	Rmin			Ravg			Rmax		
	ac	pc	ac-pc distance	ac	pc	ac-pc distance	ac	pc	ac-pc distance
*Without guideline*									
Expert 1	0.42	0.55	0.57	0.39	0.46	0.13	0.11	0.87	0.48
Expert 2	0.56	0.20	0.26	0.75	0.71	0.14	0.21	0.48	0.49
Mean	0.49	0.38	0.42	0.57	0.58	0.14	0.16	0.68	0.48
*With guideline*									
Expert 1	0.32	0.58	0.31	0.50	0.76	0.34	0.44	0.84	0.98
Expert 2	0.45	0.24	0.26	0.32	0.94	0.09	0.26	0.83	0.44
Mean	0.38	0.41	0.29	0.41	0.85	0.22	0.35	0.84	0.71

#### Midpoints location errors

The analysis of midpoint location error (i.e., Euclidean distances) was conducted exclusively under the guideline condition. Without the guideline, experts tended to select random points along the midline, resulting in a lack of consistency and correspondence. The differences in identifying the spatial locations for midpoint 1 are predominantly within 1 voxel (0.8 mm) difference, with two exceptions where the errors reach up to 1 mm for *R*_min_ and 0.82 mm for *R*_max_. A similar trend is observed for midpoint 2, where most of the errors are less than 1 voxel difference, except for *R*_min_, which yielded an error of 0.93 mm. The errors increase for midpoints 3 and 4. For midpoint 3, the largest errors are observed for *R*_max_ (1.56 mm and 2.06 mm), while the minimal error occurs for *R*_avg_ (0.79 mm and 1.05 mm). For *R*_min_, the error range is between 1.05 mm and 1.55 mm. The differences for midpoint 4 are highest for *R*_max_ (1.75 mm and 2.33 mm) and lower in *R*_avg_ (0.31 mm and 0.64 mm). For *R*_min_, the errors are 0.39 mm and 1.20 mm.

While the experts demonstrated millimeter accuracy in identifying Midpoints 1 and 2, a slight deviation was observed in the identification of Midpoints 3 and 4. Thus, accurately identifying specific points within thin, translucent structures such as the falx cerebri proves challenging, even for experts (Table 3).

**Table 3:** Comparison of errors in midpoint locations. This table illustrates the errors (in mm) in Spatial locations of four midpoints. comparing results between experts and baseline

	Midpoint 1			Midpoint 2			Midpoint 3			Midpoint 4		
	Rmin	Ravg	Rmax	Rmin	Ravg	Rmax	Rmin	Ravg	Rmax	Rmin	Ravg	Rmax
Expert 1	0.34	0.53	0.82	0.93	0.26	0.51	1.05	0.79	2.06	0.39	0.31	2.33
Expert 2	1.00	0.67	0.35	0.60	0.35	0.74	1.55	1.05	1.56	1.20	0.64	1.75
Mean	0.67	0.60	0.58	0.76	0.30	0.62	1.30	0.92	1.81	0.80	0.48	2.04

#### Root-mean-square deviation (RMSD)

RMSD analysis revealed that experts performed better in the *R*_avg_ and *R*_max_ rotation conditions compared to *R*_min_, where the largest deviations were observed. Nevertheless, the overall mean deviation across all three rotation scenarios remained below 1.5 mm, regardless of guideline use. Although adherence to the guideline resulted in slightly improved AC–PC alignment, the difference was marginal and inconsequential (Table 4).

**Table 4:** RMSD values (in mm), computed from rotation matrices, quantifying the deviation of experts manual transformations from the baseline

	Rmin	Ravg	Rmax	Mean
* Without guideline*				
Expert 1	1.45	1.07	1.74	1.42
Expert 2	2.17	1.29	0.60	1.35
*With guideline*				
Expert1	2.04	1.22	0.85	1.37
Expert2	0.85	1.22	1.54	1.20

#### Rotational errors

The detailed analysis of these rotational errors (Table 5) shows that, in the absence of the guideline, prominent rotation errors were made around the X-axis, with maximum deviations of 1.52°, 1.12°, and 1.79°, for *R*_min_, *R*_avg_, and *R*_max_ respectively. While most rotation errors around the Y- and Z-axes remained below 1°, a few exceptions were noted specifically: deviations of 1.44° for *R*_min_ and 1.39° for *R*_avg_. The only notable Z-axis deviation without the guideline was 0.94°, observed for *R*_min_.

Similarly, when the guideline was applied, notable rotation errors were predominantly observed around the X-axis across all three rotation scenarios. The maximum recorded deviations were as follows: *R*_min_ = 2.13°, *R*_avg_ = 1.35°, and *R*_max_ = 1.48. In contrast to the no-guideline condition, rotation errors around the Y- and Z-axes consistently remained below 1° with the guideline in place. In summary, although the observed differences were relatively minor, noticeable deviations persisted in both conditions.

**Table 5:** Rotation angles (in degrees) for all three axes, comparing the baseline (shown in bold) with expert assessments under both guideline and non-guideline conditions. Additionally, it shows the absolute difference values (in brackets) between the experts’ measurements and the baseline

	Rmin			Ravg			Rmax		
	X	Y	Z	X	Y	Z	X	Y	Z
*Without guideline*									
**Baseline**	**−11.65**	**−0.35**	**0.09**	**8.54**	**3.48**	**2.26**	**27.27**	**−0.74**	**−4.58**
Expert 1	−10.15(1.50)	−0.59(0.24)	0.07(0.01)	9.66(1.12)	3.82(0.34)	2.45(0.18)	29.06(1.79)	−1.02(0.28)	−4.19(0.39)
Expert 2	−10.13(1.52)	1.09(1.44)	−0.85(0.94)	8.80(0.26)	4.87(1.39)	2.48(0.21)	27.22(0.05)	−0.25(0.49)	−4.16(0.42)
*With guideline*									
Expert 1	−9.52(2.13)	−0.39(0.04)	0.33(0.24)	9.88(1.35)	3.43(0.05)	2.44(0.18)	28.01(0.74)	−0.21(0.52)	−4.67(0.10)
Expert 2	−10.84(0.81)	0.00(0.36)	0.11(0.02)	7.23(1.31)	3.67(0.19)	2.48(0.21)	25.79(1.48)	−0.04(0.70)	−4.68(0.10)

### Non-expert performance with the guideline

Three rotated images (*R*_min_, *R*_avg_, *R*_max_) were rearranged in four unique orders using permutation, resulting in four subgroups, each containing three images. four non-experts were tasked with realigning these rotated images to the AC-PC plane using the precursor version of the guideline. The non-experts received both PDF and video versions of the guideline at least two hours before the experiment and were instructed to read the guideline and watch the video instructions. The experiment commenced once the non-experts were ready to perform the alignment. They were allowed to review the instructional video or the PDF guideline during the experiment. To evaluate the performance of the non-experts, we compared the spatial locations of the borders of the AC and the PC, And four midline points, as well as the rotation angles and RMSD, to the baseline.

####  AC and PC location errors

In the study of the spatial locations of the AC and the PC, non-experts demonstrated proficient performance under minimal rotation *R*_min_. The maximum errors recorded were 0.80 mm for AC, 0.50 mm for PC, and 0.75 mm for the AC-PC distance, all within a one voxel (0.8 mm) difference. However, under average rotation, *R*_avg_, the peak value increased to 1.09 mm for AC, 1.15 mm for PC, and 1.33 mm for the AC-PC distance. With further increased rotation as in *R*_max,_ the maximum errors observed were 2.16 mm for AC, 1.20 mm for PC, and 1.63 mm for the AC-PC distance. Despite a few minor deviations, the use of guidelines enabled non-experts to achieve accuracy nearly on par with that of the experts (Table 6).

**Table 6:** Comparison of errors in AC-PC location and AC-PC distance. This table illustrates the errors (in mm) in AC-PC location and AC-PC distance, comparing results between non-experts and baseline

	Rmin			Ravg			Rmax		
	ac	pc	ac-pc distance	ac	pc	ac-pc distance	ac	pc	ac-pc distance
Non-Expert 1	0.56	0.50	0.75	1.09	0.34	0.25	0.52	0.75	0.62
Non-Expert 2	0.80	0.28	0.15	0.76	0.67	0.44	2.16	0.28	1.63
Non-Expert 3	0.34	0.46	0.44	0.68	1.15	1.33	0.51	0.34	0.41
Non-Expert 4	0.33	0.29	0.07	0.46	0.64	0.01	0.15	1.20	0.61
Mean	0.51	0.38	0.35	0.75	0.70	0.51	0.84	0.64	0.82

#### Midpoint location errors

The non-experts demonstrated superior performance in identifying Midpoints 1 and 2 under rotation conditions of *R*_min_, *R*_avg_, with most errors being under 1 mm. However, two outliers were noted in *R*_avg_, where errors of 1.84 mm and 1.97 mm were recorded for Midpoints 1 and 2, respectively. In *R*_max_, the errors for Midpoint 1 ranged between 0.26 mm and 1.32 mm,

while for Midpoint 2, the errors varied from 0.71 mm to 1.32 mm. The error range for Midpoints 3 and 4 showed a notable increase, particularly under more challenging rotation conditions. For Midpoint 3, under the *R*_min_, condition, errors ranged from 0.37 mm to 2.87 mm. In the *R*_avg_, condition, errors spanned from 0.91 mm to 2.69 mm, excluding an outlier of 28.68 mm from the mean calculation. Under *R*_max_, the errors varied significantly, with one instance showing a deviation as large as 7.12 mm. Similarly, for Midpoint 4, under the *R*_min_, condition, errors ranged from 0.92 mm to 2.17 mm. In the *R*_avg_, condition, errors fell between 0.76 mm and 3.36 mm, again excluding an outlier of 23.63 mm from the mean. Under *R*_max_, errors ranged from 2.87 mm to 6.10 mm.

In summary, while non-experts demonstrated near expert-level accuracy in identifying Midpoints 1 and 2, they showed significant deviations for Midpoints 3 and 4, especially under increased rotation conditions such as *R*_avg_, *R*_max_. This highlights the challenges in accurately identifying specific points within the falx cerebri, even with the aid of guidelines (Table 7).

**Table 7:** Comparison of errors in midpoint locations (outliers excluded). This table illustrates the errors (in mm) in Spatial locations of four midpoints. comparing results between non-experts and baseline. mean values exclude identified outliers (shown in bold)

	Midpoint 1			Midpoint 2			Midpoint 3				Midpoint 4		
	Rmin	Ravg	Rmax	Rmin	Ravg	Rmax	Rmin	Ravg	Rmax	Rmin		Ravg	Rmax
Non-Expert 1	0.26	0.61	1.32	0.96	0.21	0.71	1.64	**28.68**	2.33	1.11		**23.63**	2.87
Non-Expert 2	0.56	0.68	0.62	0.99	0.46	1.10	2.87	2.69	7.12	2.17		3.36	6.10
Non-Expert 3	0.47	1.84	1.07	0.97	1.97	1.32	0.37	0.98	2.27	1.13		1.56	3.18
Non-Expert 4	0.46	0.67	0.26	0.96	0.25	0.78	2.01	0.91	0.97	0.92		0.76	3.95
Mean	0.44	0.95	0.82	0.97	0.72	0.98	1.72	1.53	3.17	1.33		1.89	4.02

#### Root-mean-square deviation (RMSD)

RMSD analysis showed that non-experts achieved lower errors in the *R*_min_ and *R*_avg_ rotation conditions than in *R*_max_, where the two largest deviations 3.38 mm and 2.82 mm were recorded (Table 8.). These outliers elevated the overall mean RMSD across the three rotation scenarios to 2.13 mm and 1.53 mm, respectively. Even so, the results indicate that, with clear guidelines, non-experts can perform as well as experts.

**Table 8:** This table presents RMSD values (in mm), computed from rotation matrices, quantifying the deviation of non-expert manual transformations from the baseline

	Rmin	Ravg	Rmax	Mean
Non-expert1	1.52	1.49	1.21	1.41
Non-expert2	1.45	1.57	3.38	2.13
Non-expert3	1.18	0.89	1.52	1.20
Non-expert4	1.18	0.60	2.82	1.53

#### Rotational errors

In the *R*_min,_ condition, the predominant rotational error was observed around the X-axis, ranging from 1.21° to 1.58°, while errors around the Y- and Z-axes remained consistently below 0.5°. For *R*_avg_, X-axis errors ranged from 0.51° to 1.70°, with most Y- and Z-axis deviations staying under 0.5°, except for a single instance where the error reached 0.96°. The non-expert group demonstrated the highest variability in *R*_max_ across all conditions, with X-axis errors fluctuating between 0.70° and 3.51°, and Y-axis errors ranging from 0.82° to 1.31°. Z-axis deviations were minimal, consistently remaining below 0.2°. These results complement the RMSD analysis, indicating that non-experts struggled to achieve proper AC-PC alignment in highly rotated images. However, with few exceptions, their accuracy across all three rotation conditions was generally comparable to that of the expert group (Table 9).

**Table 9:** Rotation angles (in degrees) for all three axes, comparing the baseline (shown in bold) with non-expert assessments. additionally, it shows the absolute difference values (in brackets) between the non-experts' measurements and the baseline

	Rmin			Ravg			Rmax		
	X	Y	Z	X	Y	Z	X	Y	Z
**Baseline**	**−11.65**	**−0.35**	**0.09**	**8.54**	**3.48**	**2.26**	**27.27**	**−0.74**	**−4.58**
Non-expert1	−10.07 (1.58)	−0.25(0.11)	0.28(0.19)	9.95 (1.41)	3.38(0.10)	3.22(0.96)	26.57 (0.70)	0.35(1.09)	−4.55(0.03)
Non-expert2	−13.16(1.51)	−0.34(0.01)	−0.10(0.19)	10.24(1.70)	3.79(0.31)	2.52(0.26)	23.76(3.51)	0.08(0.82)	−4.54(0.03)
Non-expert3	−10.44(1.21)	−0.40(0.04)	0.41(0.32)	7.62(0.92)	3.77(0.30)	2.41(0.15)	26.32(0.95)	0.57(1.31)	−4.67(0.09)
Non-expert4	−10.45(1.21)	−0.57(0.22)	0.25(0.16)	8.03(0.51)	3.88(0.41)	2.34(0.08)	24.42(2.85)	0.20(0.94)	−4.71(0.14)

### Comparisons against automated realignment methods

Three automated realignment approaches were compared against the proposed guideline (i.e. the reference baseline established in Experimental procedures section): AC-PC realignment using “acpcdetect” (Ardekani and Bachman 2009), template registration using FSL (Jenkinson et al. 2012), and realignment based on point-warping using ANTs (Avants et al. 2008) + Slicer 3D (Fedorov et al. 2012).

The fully automated AC-PC realignment (acpcdetect) tool integrated into the Automatic Registration Toolbox (ART) realigns the structural MR images to the Schaltenbrand version of the AC-PC plane (Ardekani and Bachman 2009). This tool uses a three-stage approach to align individual MR images to the AC–PC plane. In the first stage, the midsagittal plane is estimated, and the image is rotated to align with this plane (Ardekani et al. 1997) In the second stage, the AC–PC are automatically identified using a model-based algorithm, after which a transformation is computed to realign the image to the AC–PC plane (Ardekani and Bachman 2009). The third stage focuses on stabilizing this standard orientation by automatically identifying eight additional landmarks. A subsequent transformation is calculated to align these landmarks as closely as possible with predetermined target locations (Ardekani & Alzheimer’s Disease Neuroimaging, 2022). Throughout all three stages, a rigid body registration method is employed to achieve the alignment.

For the fully automated AC-PC alignment method, no specific data preparation was necessary other than ensuring that the input data was in a short or unsigned short NIfTI format (Ardekani and Bachman 2009). As for the manual realignments, we assessed the performance of this fully automated method by analyzing errors in rotation angles and calculating root mean square deviation against the baseline. Although the tool outputs the spatial locations of the AC and the PC, their convention (Schaltenbrand-center of ac to center of pc) differs from the one we adopted for the baseline (Clinical-posterior border of ac to anterior border of pc). Therefore, a direct quantitative comparison was not possible. However, we qualitatively assessed the spatial locations of the AC–PC (supplementary Figs. 25,26,27).

The linear template registration method, which is applied in the pre-processing pipeline of the WU-Minn Human Connectome Project (HCP) (Glasser et al. 2013), involves a rigid-body registration to a standard template image using 6 degrees of freedom (DOF).The rigid-body transformation matrix is derived from an initial 12 DOF affine registration to the template image. This approach preserves the shape and size of the individual subject (Glasser et al. 2013). However, an important limitation of this method is that its performance depends strongly oemen the accuracy of the underlying registration algorithm (Dadar et al. 2018) and the quality and orientation of the template image used.

Because linear template registration depends critically on template orientation, we evaluated this method in two stages. First, we followed the HCP preprocessing protocol, which involves rigidly aligning the native structural image to the MNI152 2007 T1 template (Glasser et al. 2013). Second, the MNI152 2007 T1 template was realigned to the AC–PC plane in accordance with our proposed guideline. The resulting AC–PC-aligned template was then used as the reference for rigid-body registration. This realignment was performed using FSL’s FLIRT (Greve and Fischl 2009; Jenkinson et al. 2002; Jenkinson and Smith 2001) and Apply-Warp (Andersson JLR, 2010; Glasser et al. 2013; Jenkinson et al. 2002; Jenkinson et al. 2012). The primary outputs from this approach were the transformed image and the transformation matrix. Because this method does not generate spatial landmark points, evaluation was limited to rotation-angle error and RMSD. These two metrics were therefore used as the primary indicators of the method’s accuracy and performance.

The non-linear point-warping method offers a sophisticated approach to aligning MRI images to AC-PC space (Horn et al. 2017; Pallavaram et al. 2015).This process also unfolds in two primary stages. Initially, the spatial coordinates of the AC, PC, and midline points are identified within a reference template image These landmark points are then subjected to a nonlinear transformation, which warps them onto the native structural image (Horn et al. 2017). Using these warped landmark points, the desired AC-PC line and midplane are established within the native structural image. Subsequently, the transformation matrix is computed to precisely realign the image to the AC-PC space (Horn and Kuhn 2015; Oxenford et al. 2022). This method offers the advantage of performing realignment on the native structural image based on subject-specific anatomy. Nonetheless, the efficacy of this realignment is significantly influenced by the nonlinear algorithm employed within the registration software (Klein et al. 2009; Liu and Dawant 2014), as well as the various algorithms utilized in the subsequent processing stages.

When applying the non-linear point-warping method, defining key landmark points within the chosen T1 template space, specifically MNI 152 2009b (Fonov et al. 2009),is essential. Here, these landmarks were identified using two distinct approaches: (1) as proposed by Horn et al. in LeadDBS (Horn and Kuhn 2015; Horn et al. 2017) and (2) according to our protocol proposed here. Once identified, the landmark points were non-linearly warped to the individual brain using ANTs SyN registration and point registration techniques (Avants et al. 2008). Following this, the landmarks were used to define the AC-PC line and the midplane. These definitions were then utilized to create the transformation using AC-PC transformation module in Slicer 3D software (Fedorov et al. 2012). The primary outputs of point warping methods include the spatial coordinates of the AC, PC, and midline points. Additionally, these methods produce both the transformed image aligned to the AC-PC axis and the corresponding transformation matrix. In the points registration method proposed by (Horn et al. 2017), the AC–PC landmarks also serve as midline points, alongside an additional landmark in the falx cerebri. Consequently, for this method, we could assess only the spatial locations of the AC–PC, the AC–PC distance, rotation angles, and RMSD. In contrast, the point-warping method based on the proposed guideline enabled a more comprehensive comparison, including evaluation of the spatial locations of the AC, PC, and midline points, as well as rotation-angle error and RMSD.

The automated realignment approaches were implemented in different software packages (FSL and ART), whereas the guideline-based procedure was performed in 3D Slicer. Because each tool uses its own coordinate convention and outputs transformation matrices in different formats, additional processing was required to make the results comparable. Specifically, all transformation matrices produced by acpcdetect and FSL were converted to ITK format using the C3D affine tool within ITK (Yoo et al. 2002). No conversion was needed for 3D Slicer outputs, as they are already provided in ITK format. This harmonization step standardized the transformation matrices across methods. We then derived rotation-angle errors and computed the root mean squared deviation (RMSD) from the rotation matrices for all approaches relative to the baseline.

When comparing rotation angles derived from the acpcdetect transformation matrix, we observed a sign inversion in the Y and Z rotation components. To investigate this discrepancy, we applied the original acpcdetect matrix to the rotated image to test whether it reproduced the ACPC-aligned NIfTI output generated by acpcdetect. This comparison revealed a mismatch between the reported matrix and the exported image: applying the original matrix did not produce an ACPC-aligned image, whereas the NIfTI output produced directly by acpcdetect was correctly ACPC-aligned. Notably, manually flipping the signs of the *Y* and *Z* components of the original matrix yielded an image that was properly ACPC-aligned and consistent with the acpcdetect NIfTI output. For transparency, we therefore report the angular error and RMSD values computed using both the original matrix and the Y/Z sign-corrected matrix in the supplementary material, specifically Supplementary Tables 2 and 3. In the main manuscript, we report the results corresponding to the matrix that reproducibly generated an AC–PC-aligned image consistent with the acpcdetect output, that is, the Y/Z-corrected matrix.

#### AC and PC location errors

The “acpcdetect” and linear template registration approaches did not allow for direct comparisons of the AC–PC location. The Non-linear point-warping methods adopted from (Horn and Kuhn 2015; Horn et al. 2017) and the one based on the proposed guideline exhibited comparable errors (with respect to the reference baseline defined in Experimental procedures section), although the latter demonstrated slightly better performance. Specifically, the mean errors for the AC, PC, and AC-PC distances using the Horn et al. method (Horn et al. 2017) were 1.17, 0.69 and 1.38 mm, respectively, while for guideline-based point-warping, these errors were 1.06, 0.56, and 0.82 mm. Despite these minor improvements, the differences are small and negligible.

This outcome underscores the strong alignment between the conventions for defining AC-PC locations (posterior border of the AC to the anterior border of the PC) in the proposed guidelines and those employed in the method outlined by Horn et al. (Horn and Kuhn 2015; Horn et al. 2017).The similarities indicate consistency in approach, validating the reliability of the proposed guideline (Table 10).

**Table 10:** Comparison of errors in AC-PC location and AC-PC distance. this table illustrates the errors (mm) in AC-PC location and AC-PC distance, comparing results between non-linear points warping method and baseline. the comparison is made both with Horn et al. method and our proposed guidelines method

	Non-linear Points warping method: Horn et al.			Non-linear Points warping method: proposed guideline		
	ac	pc	ac-pc distance	ac	pc	ac-pc distance
Rmin	1.52	1.06	1.86	1.22	0.74	1.25
Ravg	0.91	0.22	0.78	0.91	0.70	0.21
Rmax	1.09	0.79	1.50	1.04	0.25	0.99
Mean	1.17	0.69	1.38	1.06	0.56	0.82

#### Midpoints location errors

In the Non-linear points-warping method proposed by Horn et al., the authors selected the AC, PC, and an arbitrary point in the falx cerebri as midline markers to define the midplane. This approach differs from the landmarks we have chosen, making a direct comparison of midplane locations between the two methods not feasible. Therefore, we limited our comparison to the midpoint locations obtained from the guideline-based point-warping method and the baseline.

This approach demonstrated excellent correspondence for midpoints 1 and 2, with mean errors of less than 1 mm. However, midpoints 3 and 4 showed less accurate correspondence, with mean values of 16.64 mm and 16.97 mm, respectively. Although this deviation results in a larger error compared to the baseline, midpoints 3 and 4 are still accurately warped within the falx cerebri, ensuring precise selection of the midplane. Consequently, there were no adverse effects on the final realignment. In conclusion, while the point-warping method accurately warps points within the falx cerebri, it fails to preserve correspondence consistently (Table 11).

**Table 11:** Comparison of errors in midpoint locations. This table illustrates the errors (mm) in four midpoint locations, comparing results between nonlinear points warping method and baseline

	Midpoint1	Midpoint2	Midpoint3	Midpoint4
Rmin	0.99	1.06	15.34	26.17
Ravg	0.90	1.00	5.48	2.04
Rmax	0.28	0.57	29.11	22.69
Mean	0.72	0.88	16.64	16.97

#### Root-mean-square deviation (RMSD)

RMSD analysis revealed a clear performance hierarchy among the automated methods (Table 12). Linear template registration that relied on the generic MNI template performed worst, with a mean RMSD of 5.89 mm across the three rotation conditions and errors that grew as the rotational offset increased. Substituting an AC–PC-pre-aligned template substantially improved accuracy, reducing the mean RMSD to 2.15 mm.

The Auto-acpcdetect algorithm achieved intermediate results (mean = 3.31 mm), but its error peaked at 4.42 mm in the *R*_min_ condition. Among the fully automated techniques, the Non-linear point-warping method delivered the most precise AC–PC realignment overall. Even so, both the Horn et al. algorithm and the guideline-based procedure performed nearly identically, with only minor differences. Notably, the guideline-based approach emerged as the top performer of all automated methods, attaining the lowest mean RMSD of 1.38 mm.

**Table 12:** RMSD values (in mm) computed from rotation matrices, quantifying the deviation of automated method’s transformation from the baseline

	Rmin	Ravg	Rmax	Mean
Auto-acpcdetect	4.42	2.46	3.04	3.31
Linear Template registration: MNI	3.61	5.15	8.91	5.89
Linear Template registration: AC-PC MNI	1.35	1.23	3.87	2.15
Non-linear Points warping: Horn et al	3.73	1.05	0.55	1.78
Non-linear Points warping: guideline based	3.47	0.32	0.34	1.38

#### Rotational errors

For the automatic “acpcdetect” method, substantial rotational errors were observed around the X-axis, with deviations of 4.31°, 2.50°, and 3.01° for *R*_min_, *R*_avg_, and *R*_max_ respectively (Table 13). Errors were also notable around the Y-axis, with deviations of 1.66° and 1.23° for the *R*_min_ and *R*_max_ conditions, respectively, and around the Z-axis, with a deviation of 1.24° for *R*_min_ indicating broader alignment inaccuracies across all axes.

In comparison, the linear template registration method using the MNI 2007 template initially resulted in the highest rotational errors among the automated approaches. Specifically, the X-axis rotation errors were 3.76°, 5.61°, and 9.41° for *R*_min_, *R*_avg_, and *R*_max_ respectively. While most Y- and Z-axis errors remained below 1°, an exception was noted in the Rmax condition where the Z-axis error reached 1.62°. However, when the MNI 2007 template was first realigned to the AC–PC plane, these rotational discrepancies were significantly reduced. Post-realignment, X-axis errors dropped to 0.50°, 1.26°, and 3.70° for *R*_min_, *R*_avg_, and *R*_max_ respectively. Y-axis deviations remained consistently under 1° across all conditions, while minor Z-axis deviations of 1.17° and 2.04° were observed in the *R*_min_ and *R*_max_ conditions, respectively.

The Non-linear point warping methods demonstrated the lowest rotational errors overall. The method based on Horn et al. yielded an X-axis error of 3.72° and a Z-axis error of 1.16° in the *R*_min_ condition. In the *R*_avg_ condition, the only notable deviation above 1° occurred around the Y-axis (1.06°), while all *R*_max_ errors remained below 1°. The guideline-based point warping method achieved the highest accuracy among the automated AC–PC realignment techniques. The only rotational error exceeding 1° occurred around the X-axis in the *R*_min_ condition where the errors reached to 3.64°. These rotational errors reflecting the results of RMSD values, demonstrating that the guideline-based point warping method outperforms all other automated technique (Table 13).

**Table 13:** Rotation angles (in degrees) for all three axes, comparing the reference baseline (shown in bold) with automated methods. additionally, it shows the absolute difference values (in brackets) between the automated measurements and the reference baseline

	Rmin			Ravg			Rmax		
	X	Y	Z	X	Y	Z	X	Y	Z
**Baseline**	**−11.65**	**−0.35**	**0.09**	**8.54**	**3.48**	**2.26**	**27.27**	**−0.74**	**−4.58**
Auto acpcdetect	−15.96(4.31)	−2.02(1.66)	0.65(0.56)	6.04(2.50)	3.57(0.10)	1.02(1.24)	24.26(3.01)	−1.97(1.23)	−4.14(0.44)
Linear Template registration: MNI	−15.41(3.76)	−0.21(0.14)	0.53(0.44)	2.93(5.61)	4.41(0.93)	2.29(0.03)	17.86(9.41)	−0.13(0.60)	−2.95(1.62)
Linear Template registration: AC-PC MNI	−11.15(0.50)	−1.11(0.76)	1.25(1.17)	7.28(1.26)	3.53(0.05)	2.72(0.45)	23.57(3.70)	−1.14(0.40)	−2.53(2.04)
Non-linear Points warping: Horn et al.	−7.93(3.72)	0.14(0.49)	−1.08(1.16)	8.87(0.34)	4.54(1.06)	1.93(0.34)	27.41(0.14)	−1.30(0.57)	−4.66(0.08)
Non-linear Points warping: guideline based	−8.01(3.64)	−0.41(0.06)	0.01(0.08)	8.67(0.14)	3.80(0.32)	2.28(0.02)	27.11(0.16)	−0.85(0.12)	−4.24(0.33)

### Inter - and intra - rater reliability of landmark identification

Twelve preprocessed structural T1-weighted images were rearranged into four unique image orders using permutation, resulting in four subgroups, each containing the same 12 images in a different order. These four subgroups were divided across two sessions for two raters. Two non-expert raters were asked to realign the images to the AC-PC plane using the definitive version of the guideline. At least two hours before the experiment, the raters received both PDF and video versions of the guideline and were instructed to read the document and watch the instructional video. The experiment began once the raters indicated that they were ready to perform the alignment. During the experiment, they were permitted to consult the PDF guideline and/or the instructional video as needed. One week later, the non-expert raters repeated the experiment. To evaluate their performance, the spatial locations of the AC–PC borders, as well as four midline points, were compared between raters and between sessions using Euclidean distance errors computed with a custom Python script. Inter- and intra-rater reliability were assessed using the intraclass correlation coefficient (ICC) calculated with the Pingouin statistical package.(Vallat 2018).

####  Inter-rater reliability

##### Session1

*AC–PC landmark localization:*

**Fig. 21:**
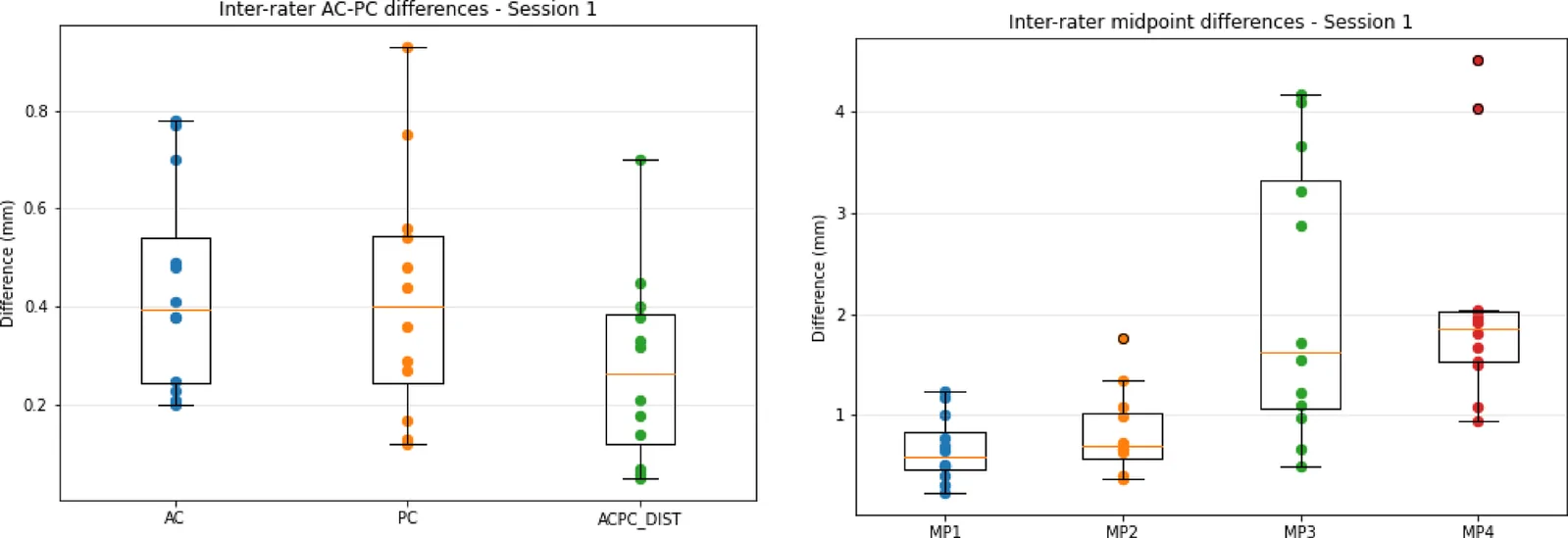
Inter-rater differences in landmark identification during Session 1. Box plots show Euclidean distance differences between Rater 1 and Rater 2 for AC–PC landmark locations and AC–PC distance on the left, and for the four midline landmarks on the right

In Session 1, inter-rater variability in AC–PC landmark localization was low. For both the AC–PC, the differences between raters were generally below 0.8 mm, corresponding to less than one voxel. Only one PC measurement reached 0.9 mm. Similarly, the differences in AC–PC distance remained below 0.8 mm, Overall, the median differences are low about 0.4 mm for AC–PC and lower for AC–PC distance indicating excellent consistency between raters in Session 1 (Fig. 21).

*Midpoint landmark localization:*

In Session 1, inter-rater variability differed across the four midpoint landmarks. MP1 and MP2 showed relatively low differences between raters, with median errors of 0.6 mm and 0.7 mm, respectively. The maximum errors for MP1 and MP2 reached 1.2 mm and 1.7 mm, respectively. In contrast, MP3 and MP4 showed substantially higher variability, with median errors of 1.6 mm and 1.8 mm, respectively. With the maximum errors reaching 4.1 mm for MP3 and 4.5 mm for MP4 with an outlier. Overall, these findings indicate good inter-rater agreement for MP1 and MP2, but poorer agreement and higher variability for MP3 and MP4.

##### Session 2

*AC–PC landmark localization:*

**Fig. 22:**
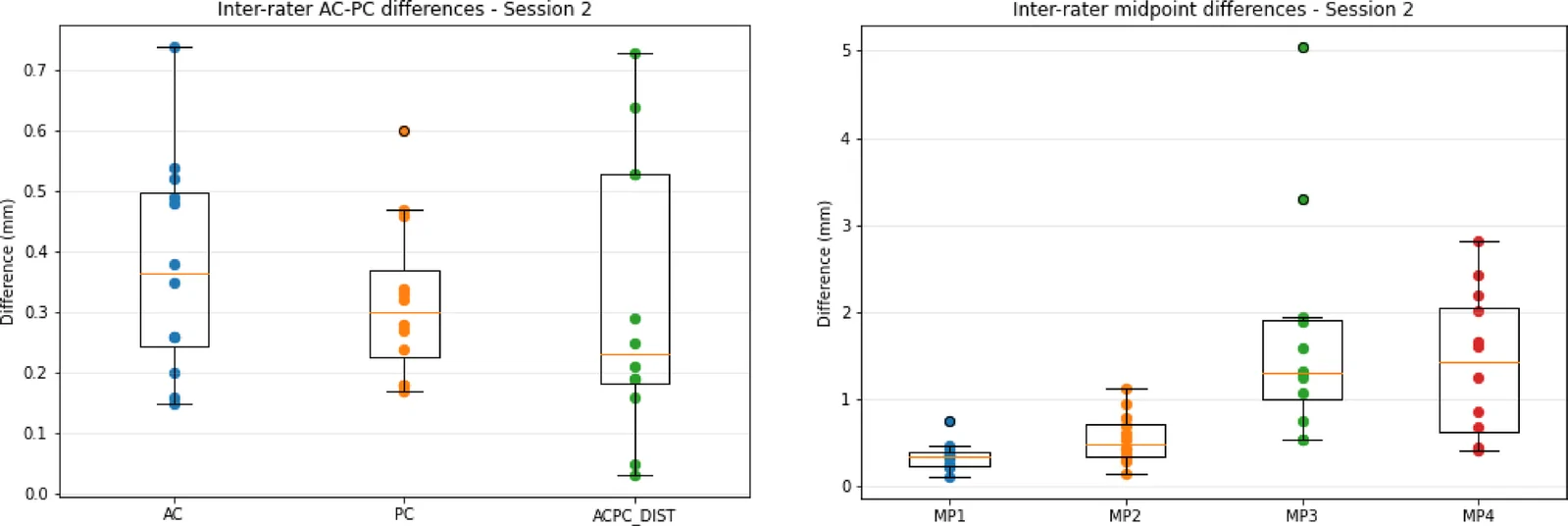
Inter-rater differences in landmark identification during Session 2. Box plots show Euclidean distance differences between Rater 1 and Rater 2 for AC–PC landmark locations and AC–PC distance on the left, and for the four midline landmarks on the right

In Session 2, AC–PC landmark localization showed lower inter-rater variability. AC differences remained below one voxel (0.8 mm), with a median of 0.35 mm. PC differences were predominantly below 0.5 mm, with one value near 0.6 mm, and a median of 0.3 mm. Similarly, AC–PC distance differences remained below 0.8 mm, with a lower median of 0.22 mm. Overall, these results indicate excellent consistency between raters in Session 2. Compared with Session 1, Session 2 shows slightly lower median errors, particularly for PC and AC–PC distance, suggesting improved inter-rater agreement (Fig. 22).

*Midpoint landmark localization:*

In Session 2, variability between raters differed across the four midpoint landmarks. Similar to the AC–PC localization results, the midpoint errors were generally reduced compared with Session 1, particularly for MP1, MP2. MP1 and MP2 showed relatively low differences between raters, with median errors below 0.5 mm and maximum errors of 0.75 mm and 1.1 mm, respectively. In contrast, MP3 and MP4 showed greater variability. MP3 had a median error of 1.3 mm, with two high outliers around 3.3 mm and 5.0 mm. MP4 had a median error of 1.4 mm, with a maximum error of 2.8 mm. Overall, the results suggest good inter-rater agreement for MP1 and MP2, but poorer agreement and greater variability for MP3 and MP4. 

*ICC analysis for inter-rater reliability:*

The ICC results show excellent inter-rater reliability across almost all landmarks and coordinate directions in both sessions. AC–PC demonstrated the strongest agreement, with ICC values consistently between 0.99 and 1.00 across X, Y, and Z coordinates in both Session 1 and Session 2. Indicating the excellent consistency between raters for AC–PC localization. For the midpoint landmarks, MP1 and MP2 also showed excellent reliability, with ICC values mostly ranging from 0.98 to 1.00. with the confidence interval ranging between 94 and 1 showing excellent agreement.

MP3 and MP4 showed generally high inter-rater reliability, although agreement was slightly reduced in the Y direction compared with the X and Z directions. For MP3, the Y-coordinate ICC was 0.95 in Session 1 and 0.96 in Session 2, while MP4 showed Y-coordinate ICC values of 0.96 in Session 1 and 0.97 in Session 2. These values still indicate good-to-excellent agreement, but they suggest relatively greater variability in Y-coordinate. For MP3, the 95% confidence intervals for the Y coordinate ranged from 0.87 to 0.99 in Session 1 and from 0.88 to 0.99 in Session 2, indicating good agreement with minor variability. In contrast, MP4 showed a much wider 95% confidence interval for the Y coordinate in Session 1, ranging from 0.09 to 0.99, suggesting greater uncertainty in landmark identification. This uncertainty improved substantially in Session 2, where the 95% confidence interval narrowed to 0.92–0.99.

Overall, MP3 and MP4 maintained good-to-excellent reliability, but the Y-coordinate results, particularly for MP4 in Session 1, indicate uncertainty and variability compared with the other landmarks and coordinate directions (Table 14).

**Table 14:** Inter-rater reliability of landmark coordinate identification between Rater 1 and Rater 2 across Session 1 and Session 2. Intraclass correlation coefficients are reported separately for the X, Y, and Z coordinates of each landmark, with 95% confidence intervals shown in parentheses

Landmarks	Inter class correlation (95% confidence interval) Session 1			Inter class correlation (95% confidence interval) Session 2		
	X	Y	Z	X	Y	Z
AC	0.99 (0.98, 1.0)	0.99 (1.0, 1.0)	1.0 (1.0, 1.0)	0.99 (1.0,1.0)	0.99 (1.0, 1.0)	1.0 (1.0, 1.0)
PC	0.99 (1.0, 1.0)	0.99 (1.0, 1.0)	1.0 (1.0, 1.0)	0.99 (0.99, 1.0)	0.99 (0.97, 1.0)	1.0 (1.0, 1.0)
MP1	0.99 (0.99, 1.0)	0.99 (0.99, 1.0)	0.99 (0.97, 1.0)	0.99 (1.0, 1.0)	0.99 (0.99, 1.0)	1.0 (1.0, 1.0)
MP2	0.98 (0.94, 0.99)	0.99 (0.99, 1.0)	1.0 (1.0, 1.0)	0.99 (0.97, 1.0)	0.99 (0.99, 1.0)	1.0 (1.0, 1.0)
MP3	0.99 (0.97, 1.0)	0.95 **(0.87**,** 0.99)**	0.99 (0.99, 1.0)	0.99 (0.99, 1.0)	0.96 **(0.88**,** 0.99)**	0.99 (0.99, 1.0)
MP4	0.98 (0.96, 1.0)	0.96 **(0.09**,** 0.99)**	0.99 (0.99, 1.0)	0.99 (0.99, 1.0)	0.97 (0.92, 0.99)	0.99 (1.0, 1.0)

The lowest confidence-interval values are shown in bold

#### Intra-rater reliability

##### Rater 1

*AC–PC landmark localization:*

**Fig. 23:**
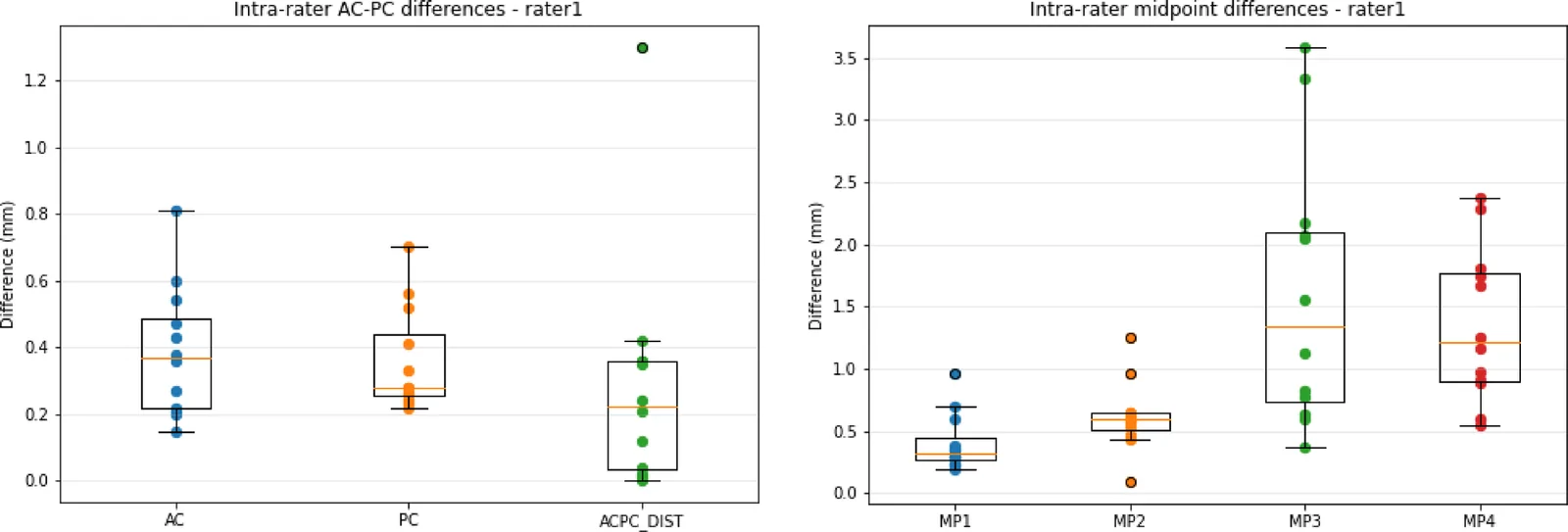
Intra-rater differences in landmark identification for Rater 1 across sessions. the left box plot shows euclidean distance differences between session 1 and session 2 for AC–PC landmark locations and AC–PC distance. The right box plot shows euclidean distance differences between sessions for the four midline landmarks

For Rater 1, intra-rater variability in AC–PC landmark localization between Session 1 and Session 2 was low. The spatial differences for both AC–PC remained below 0.8 mm, with median differences of around 0.37 mm for AC and 0.28 mm for PC. The AC–PC distance difference was also low for most cases, with a median of 0.22 mm and most values below 0.5 mm, although one outlier reached to 1.3 mm. Overall, these results indicate excellent intra-rater consistency for AC–PC localization across sessions (Fig. 23).

*Midpoint landmark localization:*

For the midpoint landmarks, intra-rater variability differed across the four locations. Rater1 showed relatively low differences between sessions for landmarks of MP1 and MP2, with median errors of 0.32 mm and 0.6 mm, respectively, indicating good intra-rater agreement. In contrast, MP3 and MP4 showed higher variability. MP3 had a median error of 1.35 mm, with maximum differences reaching to 3.6 mm, while MP4 had a median error of 1.2 mm and maximum differences reaching 2.4 mm. These findings suggest greater uncertainty in the repeated identification of MP3 and MP4 by Rater 1.

##### Rater 2

*AC–PC landmark localization:*

**Fig. 24:**
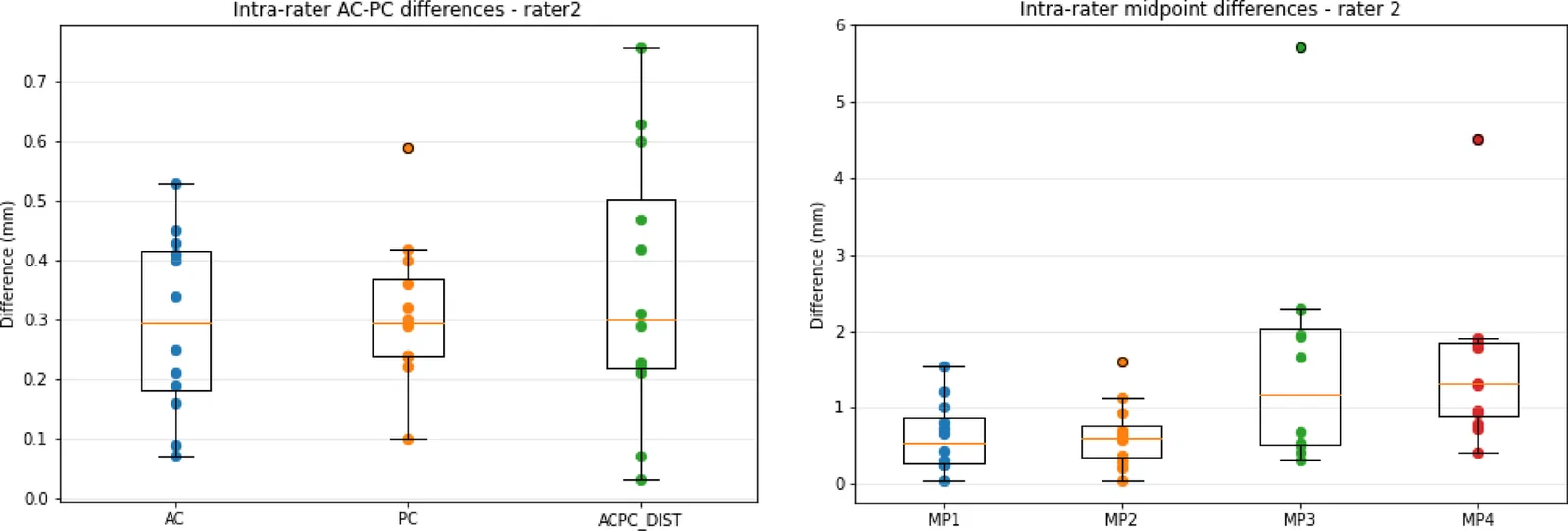
Intra-rater differences in landmark identification for Rater 2 across sessions. The left box plot shows euclidean distance differences between session 1 and session 2 for AC–PC landmark locations and AC–PC distance. The right box plot shows euclidean distance differences between sessions for the four midline landmarks

For Rater 2, AC–PC landmark localization showed low intra-rater variability between Session 1 and Session 2. The spatial differences for AC remained below 0.5 mm, with a median difference of 0.3 mm. Similarly, PC differences remained below 0.6 mm, with a median of around 0.3 mm. The AC–PC distance difference was also low overall, with a median of 0.3 mm and a maximum difference of 0.75 mm. overall these findings indicate excellent intra-rater consistency for AC– PC localization across sessions (Fig. 24).

*Midpoint landmark localization:*

For the midpoint landmarks, the degree of intra-rater variability differed across the four locations.MP1 and MP2 showed relatively low differences between sessions, with median errors of 0.5 mm and 0.6 mm, respectively. The maximum differences reached 1.5 mm for MP1 and 1.6 mm for MP2, indicating good intra-rater agreement. In contrast, MP3 and MP4 showed higher variability. MP3 had a median error of 1.2 mm, with a high outlier reaching around 5.7 mm, while MP4 had a median error of 1.3 mm, with a high outlier reaching around 4.5 mm. These findings suggest uncertainty in the repeated identification of MP3 and MP4 by Rater 2.

*ICC Analysis for intra-rater reliability:*

The intra-rater ICC analysis demonstrated excellent repeatability for both raters across most landmarks and coordinate directions. For AC–PC, both raters showed high ICC values, ranging from 0.99 to 1.00 across X, Y, and Z coordinates. This indicates near-perfect intra-rater consistency for repeated AC–PC landmark localization between sessions. For MP1 and MP2, intra-rater reliability was also excellent for both raters. ICC values were mostly between 0.98 and 1.00, with narrow 95% confidence intervals ranging between 0.96 and 1.00. MP3 and MP4 also showed high ICC values overall, but with slightly reduced agreement in the Y direction, particularly for Rater 2.

For MP3, the Y-coordinate ICC was 0.97 for Rater 1 and 0.96 for Rater 2, with 95% confidence intervals of 0.91–0.99 and 0.89–0.99, respectively. For MP4, the Y-coordinate ICC was 0.98 for Rater 1 and 0.97 for Rater 2, with Rater 2 showing a wider confidence interval of 0.88–0.99. These results suggest good-to-excellent repeatability overall, but relatively higher uncertainty in identifying MP3 and MP4 along the Y direction (Table 15).

In summary, both raters demonstrated excellent intra-rater reliability across sessions for the identification of the AC, PC, MP1, and MP2 landmarks. However, this level of reliability was not achieved for MP3 and MP4.

**Table 15:** Intra-rater reliability of landmark coordinate identification across sessions. Intraclass correlation coefficients between Session 1 and Session 2 are reported separately for Rater 1 and Rater 2, and for the X, Y, and Z coordinates of each landmark. Values in parentheses indicate 95% confidence intervals

Landmarks	Inter class correlation (95% confidence interval) Rater 1			Inter class correlation (95% confidence interval) Rater 2		
	X	Y	Z	X	Y	Z
AC	0.99 (0.99, 1.0)	0.99 (0.99, 1.0)	1.0 (1.0, 1.0)	0.99 (1.0, 1.0)	0.99 (0.99, 1.0)	1.0 (1.0, 1.0)
PC	0.99 (0.99, 1.0)	0.99 (1.0, 1.0)	1.0 (1.0, 1.0)	0.99 (1.0, 1.0)	0.99 (0.98, 1.0)	1.0 (1.0, 1.0)
MP1	0.99 (1.0, 1.0)	0.99 (1.0, 1.0)	1.0 (1.0, 1.0)	0.99 (0.99, 1.0)	0.99 (0.96, 1.0)	0.99 (0.99, 1.0)
MP2	0.98 (0.96, 1.0)	0.99 (1.0, 1.0)	1.0 (1.0, 1.0)	0.99 (0.97, 1.0)	0.99 (0.99, 1.0)	1.0 (1.0, 1.0)
MP3	0.99 (1.0, 1.0)	0.97 (0.91, 0.99)	0.99 (0.99, 1.0)	0.99 (0.97, 1.0)	0.96 **(0.89**,** 0.99)**	0.99 (0.99, 1.0)
MP4	0.98 (0.96, 1.0)	0.98 (0.96, 1.0)	0.99 (0.99, 1.0)	0.99 (0.98, 1.0)	0.97 **(0.88**,** 0.99)**	0.99 (0.99, 1.0)

The lowest confidence-interval values are shown in bold

## Discussion

The present study proposes a comprehensive guideline for realigning structural MR images to the clinically accepted definition of the AC–PC plane. The guideline provides a step-by-step procedure tailored to T1-weighted structural images, with the aim of supporting accurate and reproducible alignment to the AC–PC plane. In addition, it includes criteria for assessing the quality of the final AC–PC alignment. Although these criteria are incorporated as the final step of the protocol, they may also be used independently to evaluate existing AC–PC realignments. Detailed descriptions of the anatomical and imaging characteristics of the structures used in the guideline, including the AC, PC, and four midline landmarks—the superior pontine notch, the fastigium of the fourth ventricle, and two anterior and posterior points within the falx cerebri—are provided in the Supplementary Material.

The proposed guideline offers a structured and reproducible approach for defining the AC–PC line, establishing the midsagittal plane, and verifying the final AC–PC alignment. Although the protocol is implemented in 3D Slicer (Fedorov et al. 2012). the anatomical principles underlying the guideline are software independent. This supports its broader applicability across different manual, semi-automated, and automated neuroimaging workflows. Specifically, the guideline describes how to define the clinically accepted AC–PC line, establish the midplane using four midline landmarks, and verify the resulting AC–PC alignment.

The final guideline represents a refined version of the precursor protocol used during the first and second stages of evaluation. Based on observations and feedback from these stages, several refinements were incorporated to improve the clarity of landmark identification, AC–PC realignment, and verification procedures. A detailed account of these modifications is provided in Supplementary Material, Sect. 7, “Guideline Amendments.” The refined guideline was subsequently evaluated in the third-stage experiment, which focused specifically on the influence of the guideline on landmark identification. Overall, the current validated version provides clearer instructions for precise landmark identification, AC–PC realignment, and verification of the final alignment.

The first-stage experiment showed that manual observers were generally accurate in AC–PC realignment across the three rotated image conditions, although minor rotational deviations were observed. The largest deviations occurred around the X-axis, particularly for non-expert 2 and non-expert 4, with errors of approximately 3.51° and 2.85°, respectively. Within the expert group, Expert 1 showed the largest deviations, with errors of approximately 2.13° and 1.79°. These discrepancies likely reflect subtle differences in how observers interpreted the AC–PC line relative to the reference baseline. Because the AC–PC are separated by only approximately 23 mm on average (Schaltenbrand et al. 1977) even small differences in landmark placement can produce measurable rotational deviations (Ardekani & Alzheimer’s Disease Neuroimaging, 2022; Evans et al. 1992). as illustrated in Supplementary Figs. 28–31. These findings highlight the importance of accurate and consistent AC–PC identification. Overall, experts performed comparably with and without the guideline, suggesting consistency between routine clinical practice and the proposed protocol. Importantly, non-experts generally achieved AC–PC realignment accuracy similar to that of experts across different rotational conditions, with only a few exceptions. These findings support the utility of the guideline for enabling reliable AC–PC realignment by observers with limited prior anatomical expertise and suggest that it may help make high-precision alignment more accessible to a broader range of users.

The guideline may also serve as a standardized protocol for experts by reducing inter-observer variability, which remains a known challenge in AC–PC localization particularly when image quality is suboptimal (Pallavaram et al. 2008). This issue was reflected during validation of the precursor guideline: when experts were required to select at least three midline points, there was no correspondence among their selections, as the selected points varied randomly along the midline. By providing a systematic approach to landmark selection, the guideline addresses this variability and promotes greater consistency in the identification process.

The third-stage experiment further supports the reliability of the revised guideline at the level of landmark identification. In this stage, two additional non-expert raters identified the AC, PC, and four midline points across two separate sessions. The low inter- and intra-rater differences for the AC–PC landmarks, with median errors of approximately 0.4 mm or less, indicate that novice observers can consistently identify the primary landmarks required for AC–PC alignment when using the guideline. This finding highlights the potential value of the protocol as a training and standardization tool for users with limited prior experience in neuroanatomy or neuroimaging. The reliability analysis also showed good correspondence for MP1 and MP2, with median differences of approximately 0.7 mm or less. Greater variability was observed for MP3 and MP4, where median differences increased to approximately 1.8 mm. This pattern is consistent with the first-stage experiment, in which both expert and non-expert observers showed excellent correspondence in AC, PC, MP1 and MP2 and greater variability for MP3 and MP4.

Although this variability did not substantially affect overall AC–PC alignment accuracy, MP3 and MP4 remained the most challenging landmarks to identify consistently. This difficulty may be explained by the anatomical nature of the falx cerebri, which is a thin, translucent midline structure that can be less clearly defined on structural MRI than the AC, PC, fastigium (MP1), or superior pontine notch (MP2). As a result, the anterior and posterior falx points (MP3 and MP4) may be more susceptible to small differences in observer interpretation and may be harder to select with precise correspondence across raters or sessions. Therefore, these two landmarks may require additional attention, targeted training, or quality-control checks in future applications of the protocol.

A comparison of the automated realignment methods against the task-matched expert reference baseline showed that the nonlinear point-warping approach produced the most accurate AC–PC alignment. In this method, anatomical landmarks defined in template space were nonlinearly warped to the subject-specific T1-weighted image using ANTs SyN registration (Avants et al. 2008). The warped AC, PC, and midline landmarks were then used to calculate the transformation required for AC–PC realignment in the subject’s native T1 space. This approach outperformed both the fully automated acpcdetect method and the linear template-registration method.

The superior performance of the nonlinear point-warping approach is likely related to its ability to transfer subject-specific landmark information more accurately than linear template registration. In particular, the method identified the AC–PC with high precision, consistent with previous work demonstrating the value of nonlinear registration for AC–PC landmark localization (Liu and Dawant 2014). Our results further showed that this approach accurately localized MP1 and MP2. Although MP3 and MP4 showed greater spatial deviation, these points were still mapped within the falx cerebri and did not substantially affect midplane definition or overall AC–PC alignment accuracy. These deviations likely reflect local deformation effects inherent to nonlinear registration. The guideline-based point-warping method performed slightly better than the method adapted from Horn et al. (Horn et al. 2017). although the differences were small and negligible.

In contrast, the linear template-registration method showed suboptimal performance when the standard MNI152 template was used as the reference. The largest deviations for this method were observed around the X-axis, with errors of 3.76°, 5.61°, and 9.41° for *R*_min_, *R*_avg_, and *R*_max_ respectively. These large errors are likely attributable to differences between the orientation of the MNI152 template and the clinically used Schaltenbrand style AC–PC plane. Notably, these errors were substantially reduced when the MNI template was first realigned to the clinical AC–PC plane, with corresponding X-axis errors decreasing to 0.50°, 1.26°, and 3.70° for *R*_min_, *R*_avg_, and *R*_max_, respectively. These findings demonstrate that registering an image to the standard MNI152 template is not sufficient when the objective is to align the image to the clinical AC–PC plane or Schaltenbrand or Talairach version of AC-PC planes (Horn et al. 2017). They also emphasize the importance of careful template selection and template preparation according to the intended application.

The fully automated acpcdetect method also showed notable deviations from the expert reference baseline, with rotational errors of 4.31°, 2.50°, and 3.01° for *R*_min_, *R*_avg_, and *R*_max_, respectively. To the best of our knowledge, acpcdetect is the only model based open-source tools that provides fully automated transformation of structural MR images to the Schaltenbrand AC–PC plane. This makes it a valuable tool for researchers working with DBS datasets or large-scale image realignment tasks. However, our validation suggests that its performance may be limited in some cases. Closer inspection indicated that the model-based approach consistently failed to localize the PC accurately across the three test cases, whereas the AC was identified relatively closer to its expected location. Because accurate definition of the AC–PC line depends on both landmarks, PC localization error propagated into the final image realignment and reduced overall alignment accuracy, as illustrated in Supplementary Figs. 25–27.

Overall, the automated-method comparison indicates that nonlinear point warping of anatomical landmarks into subject space, followed by subject-specific realignment, was the most accurate automated strategy tested in this study. The results also highlight two important considerations. First, template-based registration is highly dependent on whether the template is defined according to the target AC–PC convention. Second, fully automated model-based approaches may fail when AC or PC localization is inaccurate. These findings support the value of the proposed guideline beyond manual AC–PC realignment, particularly as a standardized anatomical reference for interpreting, comparing, and validating different automated AC–PC alignment strategies.

Although automated methods are often preferred for large datasets, they can vary substantially in how they achieve AC–PC realignment and how they define AC–PC space. It is therefore important to recognize differences in AC–PC line definitions across atlas conventions and automated tools (Horn et al. 2017; Nowinski 2001). As demonstrated in our precursor guideline validation, even small deviations in AC–PC line definition can lead to substantial alignment errors and potentially misleading interpretations. Automated AC–PC alignment methods also have algorithm-specific limitations, including registration failure and inaccurate landmark identification in both registration-based and model-based approaches (Aisyah et al. 2020; Liu and Dawant 2014, 2015). In this context, the proposed manual guideline may be integrated into automated neuroimaging workflows as a standardized quality-control and correction procedure. For example, it can be used to inspect automated outputs, manually realign images when automated methods fail, prepare clinically appropriate templates, or provide consistent landmark definitions in template space for subsequent warping to individual subject space.

Another important contribution of the proposed guideline is its potential role in the development and validation of new automated AC–PC landmark-detection methods. With the rapid advancement of machine-learning, deep-learning and computer vision techniques, there has been increasing interest in automating AC–PC landmark identification (Aisyah et al. 2020; Ardekani and Bachman 2009; Edwards et al. 2021; Gohel et al. 2023; Liu and Dawant 2014, 2015). In this context, the proposed guideline may be useful not only for standardizing the images used in model development, but also for enabling the creation of consistent expert-curated ground-truth annotations. For example, Edwards et al. (Edwards et al. 2021). developed a deep-learning pipeline for automated AC–PC landmark localization using a large dataset of 1,128 manually annotated T1-weighted MR images. Their annotations were generated by raters with varying levels of expertise, ranging from novice annotators to an experienced neurosurgeon, and approximately 10% of the labelled points required manual readjustment by an expert. This example highlights the importance of a standardized annotation protocol when generating ground-truth labels for automated methods.

The present results suggest that the proposed guideline can support this process by improving consistency in AC–PC landmark identification, even among non-expert observers, as demonstrated by the inter- and intra-rater reliability analysis. Thus, the guideline may reduce variability in manual annotations and support the generation of more consistent training and validation datasets for automated AC–PC detection methods. This approach could also be extended to other anatomical landmarks, such as the fastigium or superior pontine notch, thereby facilitating the development and validation of automated tools for both clinical and research applications (Lau et al. 2019; Oba et al. 2005; Roelants et al. 2016).

To the best of our knowledge, no published work currently provides a comprehensive protocol that combines a step-by-step procedure for aligning structural MR images to the clinically used AC–PC plane with detailed guidance on the anatomical landmarks required for this process. Although many software packages that use AC–PC alignment provide brief instructions or visual aids for identifying the AC, PC, and, in some cases, midline points, these resources often provide limited anatomical and neuroimaging detail. For example, they typically do not include detailed descriptions of landmark morphology, imaging characteristics, or differences in AC–PC line definitions across clinical and atlas-based conventions. In the present work, these anatomical and imaging characteristics are provided in the Supplementary Material to support standardized landmark identification and improve the reproducibility of AC–PC realignment.

Previous efforts related to AC–PC realignment protocols are nevertheless noteworthy. Ramirez et al. (Ramirez et al. 2014) introduced a standardized, video-guided protocol for semi-automatic brain region extraction that includes a step for aligning images to the AC–PC plane. Although AC–PC realignment was not the primary objective of their study, the accompanying video and text instructions can be used for manual realignment of structural images to this plane. Their work also outlines a set of validation criteria that we initially adopted in our precursor guideline. Hamilton et al. (Hamilton et al. 2017) also provided brief instructions for manual AC–PC realignment, which they used to standardize individual brain images for subsequent processing and analysis. In addition, Lau et al. (Lau et al. 2019) developed a method for evaluating image correspondence using 32 anatomical fiducials, including the AC, PC, and several midline structures. Although AC–PC realignment was not the primary focus of that work, the method can also be used to support realignment to the AC–PC plane.

While these efforts provide useful instructions for manual AC–PC realignment, they generally assume prior knowledge of the anatomical and imaging characteristics of the AC, PC, and related midline structures, as well as familiarity with the different definitions and purposes of AC–PC realignment. This can present a barrier for novice observers, especially because AC–PC realignment is often treated as a minor step within a broader workflow rather than as the central focus of the method. The present guideline addresses this gap by providing a standardized, detailed, and practical approach to identifying the anatomical structures required for AC–PC transformation in structural MRI, thereby improving clarity, consistency, and reliability in the realignment process.

## Limitations

Several limitations should be acknowledged. First, although the reference baseline used in the first two evaluation stages was established by an expert who was not involved as an evaluated observer and was generated under the same rotation and imaging conditions as the evaluation images, it was based on a single expert realignment. Therefore, this baseline should be interpreted as a task-matched expert reference rather than an absolute anatomical ground truth. Future studies using multiple independent expert annotations or consensus-based references would further strengthen anatomical validation.

Second, although AC, PC, MP1, and MP2 were identified with high consistency, MP3 and MP4 showed greater variability across raters and sessions. This likely reflects the difficulty of identifying points on the falx cerebri, a thin midline structure that can be less clearly delineated on structural MRI. Although this variability did not substantially affect overall AC–PC alignment accuracy in the present study, MP3 and MP4 may not provide submillimeter-level consistency if the guideline is used specifically for separate landmark identification of anterior and posterior points within the falx cerebri. Therefore, these landmarks may require additional attention and quality-control checks in future applications of the protocol.

Third, although the study included an initial degree of anatomical and population variability by using images from a healthy young adult, a healthy older adult, and a patient with Parkinson’s disease, and by rotating these images to known degrees, the rotation-based validation included only one image from each group. Therefore, the findings should be interpreted as an initial validation rather than a comprehensive assessment across all anatomical presentations. The protocol was not tested in pediatric populations or in cases with focal lesions, major anatomical abnormalities, or pathology-related distortion of midline anatomy. Lesions or abnormalities involving the AC, PC, or adjacent commissural structures may limit the applicability of the guideline and require expert anatomical judgment or alternative reference strategies.

Fourth, although the validation dataset included 15 images in total, of which 12 were used for the third-stage inter- and intra-rater reliability assessment, only 3 images were included in the first two stages of the rotation-based validation. Although this was sufficient to demonstrate the effect of the guideline on ACPC alignment, the modest sample size in the rotation-based assessment limits the comprehensiveness of this validation and may reduce the generalizability of the findings.

Finally, the present study focused on AC–PC landmark identification, manual realignment, and comparison with existing automated AC–PC alignment methods. It did not evaluate the effect of AC–PC alignment on downstream tasks such as registration accuracy or segmentation quality. Such analyses would require task-specific reference standards, including registration benchmarks or expert segmentation ground truth, as well as systematic testing across different algorithms, transformation models, and datasets. Future work should investigate whether standardized AC–PC alignment improves downstream clinical and resarch neuroimaging workflows.

## Conclusion

In conclusion, the proposed guideline provides a standardized and practical framework for AC–PC realignment of structural MR images according to the clinically used AC–PC plane. The validation results indicate that the guideline supports reliable landmark identification and accurate realignment, including among observers with limited prior anatomical expertise. In addition to its use for manual realignment, the guideline may serve as a quality-control tool for automated methods and as a reference framework for developing and validating future AC–PC landmark-detection approaches.

## Supplementary Information

Below is the link to the electronic supplementary material.

Supplementary Material 1

## Data Availability

Due to data-sharing restrictions associated with the POM study, the original and derived NIfTI files from POM cannot be made publicly available. However, all non-restricted derivative outputs used in this work—including extracted rotation matrices/parameters, source images used for figure generation, and the analysis scripts (Python)—are available in the repository. Access to the restricted POM NIfTI files may be considered upon reasonable request to the corresponding author and subject to the POM data-access terms.The materials supporting the findings of this study have been deposited in the Radboud Repository. https://doi.org/10.34973/e19m-gp76.

## References

[CR1] Acar F, Miller JP, Berk MC, Anderson G, Burchiel KJ (2007) Safety of anterior commissure-posterior commissure-based target calculation of the subthalamic nucleus in functional stereotactic procedures. Stereotact Funct Neurosurg 85(6):287–291. 10.1159/000107361 17709981

[CR2] Aisyah KN, Fatichah C, Sarno R (2020) Multilevel thresholding and Morphological Relationship Approach for Automatic Detection of Anterior and Posterior Commissure in Mid-sagittal Brain MRI. Int J Intell Eng Syst 13:368–378

[CR3] Alaminos Bouza A (2014) *Imaging, Stereotactic Space and Targeting*

[CR4] Alkan C, Cocjin JB (2017) Magnetic Resonance Contrast Prediction Using Deep Learning

[CR5] Amorosino G, Peruzzo D, Redaelli D, Olivetti E, Arrigoni F, Avesani P (2022) DBB - A Distorted Brain Benchmark for Automatic Tissue Segmentation in Paediatric Patients. NeuroImage 260:119486. 10.1016/j.neuroimage.2022.119486 35843515

[CR6] Andersson JLR, Smith JM (2010) S *Non-linear registration, aka spatial normalisation. FMRIB technical report TR07JA2*

[CR7] Ardekani BA, Bachman AH (2009) Model-based automatic detection of the anterior and posterior commissures on MRI scans. NeuroImage 46(3):677–682. 10.1016/j.neuroimage.2009.02.030 19264138 PMC2674131

[CR8] Ardekani BA, Alzheimer’s Disease Neuroimaging, I. (2022) A new approach to symmetric registration of longitudinal structural MRI of the human brain. J Neurosci Methods 373:109563. 10.1016/j.jneumeth.2022.109563 35288224 PMC9008769

[CR9] Ardekani BA, Kershaw J, Braun M, Kanno I (1997) Automatic detection of the mid-sagittal plane in 3-D brain images. IEEE Trans Med Imaging 16(6):947–952. 10.1109/42.650892 9533596

[CR10] Avants BB, Epstein CL, Grossman M, Gee JC (2008) Symmetric diffeomorphic image registration with cross-correlation: evaluating automated labeling of elderly and neurodegenerative brain. Med Image Anal 12(1):26–41. 10.1016/j.media.2007.06.004 17659998 PMC2276735

[CR11] Bejjani BP, Dormont D, Pidoux B, Yelnik J, Damier P, Arnulf I, Bonnet AM, Marsault C, Agid Y, Philippon J, Cornu P (2000) Bilateral subthalamic stimulation for Parkinson’s disease by using three-dimensional stereotactic magnetic resonance imaging and electrophysiological guidance. J Neurosurg 92(4):615–625. 10.3171/jns.2000.92.4.0615 10761650

[CR12] Bhanu Prakash KN, Hu Q, Aziz A, Nowinski WL (2006) Rapid and automatic localization of the anterior and posterior commissure point landmarks in MR volumetric neuroimages. Acad Radiol 13(1):36–54. 10.1016/j.acra.2005.08.023 16399031

[CR13] Bot M, Schuurman PR, Odekerken VJJ, Verhagen R, Contarino FM, De Bie RMA, van den Munckhof P (2018) Deep brain stimulation for Parkinson’s disease: defining the optimal location within the subthalamic nucleus. J Neurol Neurosurg Psychiatry 89(5):493–498. 10.1136/jnnp-2017-316907 29353236

[CR14] Brett M, Johnsrude IS, Owen AM (2002) The problem of functional localization in the human brain. Nat Rev Neurosci 3(3):243–249. 10.1038/nrn756 11994756

[CR15] Caire F, Ouchchane L, Coste J, Gabrillargues J, Derost P, Ulla M, Durif F, Lemaire JJ (2009) Subthalamic nucleus location: relationships between stereotactic AC-PC-based diagrams and MRI anatomy-based contours. Stereotact Funct Neurosurg 87(6):337–347. 10.1159/000236367 19752592

[CR16] Caire F, Maubon A, Moreau JJ, Cuny E (2011) The mamillothalamic tract is a good landmark for the anterior border of the subthalamic nucleus on axial MR images. Stereotact Funct Neurosurg 89(5):286–290. 10.1159/000329356 21849812

[CR17] Caire F, Ranoux D, Guehl D, Burbaud P, Cuny E (2013) A systematic review of studies on anatomical position of electrode contacts used for chronic subthalamic stimulation in Parkinson’s disease. Acta Neurochir (Wien) 155(9):1647–1654. 10.1007/s00701-013-1782-1. discussion 1654 23775325

[CR18] Chakravarty MM, Bertrand G, Hodge CP, Sadikot AF, Collins DL (2006) The creation of a brain atlas for image guided neurosurgery using serial histological data. NeuroImage 30(2):359–376. 10.1016/j.neuroimage.2005.09.041 16406816

[CR19] Chau W, McIntosh AR (2005) The Talairach coordinate of a point in the MNI space: how to interpret it. Neuroimage 25(2):408–416. 10.1016/j.neuroimage.2004.12.007 15784419

[CR20] Chen X, Hou X, Gao W, Zhu M, Wang Y, Wang H, Wang X, Lin Z (2010) Morphology of the adult midsagittal brainstem in relation to the reference systems MRI-based variability study. Acad Radiol 17(6):708–717. 10.1016/j.acra.2010.01.013 20350825

[CR21] Choi SH, Chi JG, Kim YB, Cho ZH (2013) Anterior commissure–posterior commissure revisited. Korean J Radiol 14(4):653–661. 10.3348/kjr.2013.14.4.653 23901324 PMC3725361

[CR22] Coenen VA, Prescher A, Schmidt T, Picozzi P, Gielen FL (2008) What is dorso-lateral in the subthalamic Nucleus (STN)?>--a topographic and anatomical consideration on the ambiguous description of today’s primary target for deep brain stimulation (DBS) surgery. Acta Neurochir (Wien) 150(11):1163–1165 discussion 1165. 10.1007/s00701-008-0136-x 18958389

[CR23] Collins DL, Neelin P, Peters TM, Evans AC (1994) Automatic 3D intersubject registration of MR volumetric data in standardized Talairach space. J Comput Assist Tomogr 18(2):192–205 8126267

[CR24] Cox RW (1996) AFNI: software for analysis and visualization of functional magnetic resonance neuroimages. Comput Biomed Res 29(3):162–173. 10.1006/cbmr.1996.0014 8812068

[CR25] Dadar M, Fonov VS, Collins DL, Alzheimer’s Disease Neuroimaging, I. (2018) A comparison of publicly available linear MRI stereotaxic registration techniques. Neuroimage 174:191–200. 10.1016/j.neuroimage.2018.03.025 29548850

[CR26] Dautkulova A, Aider OA, Teuliere C, Coste J, Chaix R, Ouachik O, Pereira B, Lemaire JJ (2025) Automated segmentation of deep brain structures from inversion-recovery MRI. Comput Med Imaging Graph 120:102488. 10.1016/j.compmedimag.2024.102488 39787737

[CR27] Davis TS, Caston RM, Philip B, Charlebois CM, Anderson DN, Weaver KE, Smith EH, Rolston JD (2021) LeGUI: a fast and accurate graphical user interface for automated detection and anatomical localization of intracranial electrodes. Front Neurosci 15:769872. 10.3389/fnins.2021.769872 34955721 PMC8695687

[CR28] Duchin Y, Shamir RR, Patriat R, Kim J, Vitek JL, Sapiro G, Harel N (2018) Patient-specific anatomical model for deep brain stimulation based on 7 Tesla MRI. PLoS One 13(8):e0201469. 10.1371/journal.pone.0201469 30133472 PMC6104927

[CR29] Edwards CA, Goyal A, Rusheen AE, Kouzani AZ, Lee KH (2021) DeepNavNet: automated landmark localization for neuronavigation. Front Neurosci 15:670287. 10.3389/fnins.2021.670287 34220429 PMC8245762

[CR30] Evans AC, Marrett S, Neelin P, Collins L, Worsley K, Dai W, Milot S, Meyer E, Bub D (1992) Anatomical mapping of functional activation in stereotactic coordinate space. NeuroImage 1(1):43–53. 10.1016/1053-8119(92)90006-9 9343556

[CR31] Evans AC, Collins DL, Mills SR, Brown ED, Kelly RL, Peters TM (1993) 31 Oct.–6 Nov. 1993). 3D statistical neuroanatomical models from 305 MRI volumes. 1993 IEEE Conference Record Nuclear Science Symposium and Medical Imaging Conference

[CR32] Evans AC, Janke AL, Collins DL, Baillet S (2012) Brain templates and atlases. NeuroImage 62(2):911–922. 10.1016/j.neuroimage.2012.01.024 22248580

[CR33] Fedorov A, Beichel R, Kalpathy-Cramer J, Finet J, Fillion-Robin JC, Pujol S, Bauer C, Jennings D, Fennessy F, Sonka M, Buatti J, Aylward S, Miller JV, Pieper S, Kikinis R (2012) 3D Slicer as an image computing platform for the Quantitative Imaging Network. Magn Reson Imaging 30(9):1323–1341. 10.1016/j.mri.2012.05.001 22770690 PMC3466397

[CR34] Fonov VS, Evans AC, McKinstry RC, Almli CR, Collins DL (2009) Unbiased nonlinear average age-appropriate brain templates from birth to adulthood. NeuroImage 47:S102. 10.1016/S1053-8119(09)70884-5

[CR35] Fox PT, Kall B (1987) B. Stereotaxy as a Means of Anatomical Localization in Physiological Brain Images: Proposals for Further Validation. J Cereb Blood Flow Metabolism 7(1suppl):S18–S20. 10.1038/jcbfm.1987.31 6389580

[CR36] Fox PT, Perlmutter JS, Raichle ME (1984) Stereotactic method for determining anatomical localization in physiological brain images. J Cereb Blood Flow Metab 4(4):634. 10.1038/jcbfm.1984.91 6389580

[CR37] Gildenberg PL, Tasker RR, Lozano AM (2009) Textbook of stereotactic and functional neurosurgery, 2nd ed. Springer-Verlag. 10.1007/978-3-540-69960-6

[CR38] Glasser MF, Sotiropoulos SN, Wilson JA, Coalson TS, Fischl B, Andersson JL, Xu J, Jbabdi S, Webster M, Polimeni JR, Van Essen DC, Jenkinson M, Consortium WU-MH (2013) The minimal preprocessing pipelines for the Human Connectome Project. NeuroImage 80:105–124. 10.1016/j.neuroimage.2013.04.127 23668970 PMC3720813

[CR39] Gohel B, Kumar L, Shah D (2023) Deep learning-based Automated Localisation of Anterior Commissure and Posterior Commissure Landmarks in 3D space from three-plane 2D MRI localiser slices of the brain. Procedia Comput Sci 218:1027–1032. 10.1016/j.procs.2023.01.082

[CR40] Greve DN, Fischl B (2009) Accurate and robust brain image alignment using boundary-based registration. NeuroImage 48(1):63–72. 10.1016/j.neuroimage.2009.06.060 19573611 PMC2733527

[CR41] Hamilton LS, Chang DL, Lee MB, Chang EF (2017) Semi-automated Anatomical Labeling and Inter-subject Warping of High-Density Intracranial Recording Electrodes in Electrocorticography. Front Neuroinform 11:62. 10.3389/fninf.2017.00062 29163118 PMC5671481

[CR42] Hebb AO, Miller KJ (2010) Semi-automatic stereotactic coordinate identification algorithm for routine localization of Deep Brain Stimulation electrodes. J Neurosci Methods 187(1):114–119. 10.1016/j.jneumeth.2009.12.016 20036691

[CR43] Horn A, Kuhn AA (2015) Lead-DBS: a toolbox for deep brain stimulation electrode localizations and visualizations. NeuroImage 107:127–135. 10.1016/j.neuroimage.2014.12.002 25498389

[CR44] Horn A, Kuhn AA, Merkl A, Shih L, Alterman R, Fox M (2017) Probabilistic conversion of neurosurgical DBS electrode coordinates into MNI space. Neuroimage 150:395–404. 10.1016/j.neuroimage.2017.02.004 28163141 PMC6050588

[CR45] Hyam JA, Akram H, Foltynie T, Limousin P, Hariz M, Zrinzo L (2015) What You See Is What You Get: Lead Location Within Deep Brain Structures Is Accurately Depicted by Stereotactic Magnetic Resonance Imaging. Neurosurgery 11 Suppl 3:412–419. 10.1227/NEU.0000000000000848. discussion 419 26087006

[CR46] Jansen MG, Zwiers MP, Marques JP, Chan KS, Amelink JS, Altgassen M, Oosterman JM, Norris DG (2024) The Advanced BRain Imaging on ageing and Memory (ABRIM) data collection: study design, data processing, and rationale. PLoS One 19(6):e0306006. 10.1371/journal.pone.0306006 38905233 PMC11192316

[CR47] Jenkinson M, Smith S (2001) A global optimisation method for robust affine registration of brain images. Med Image Anal 5(2):143–156. 10.1016/s1361-8415(01)00036-6 11516708

[CR48] Jenkinson M, Bannister P, Brady M, Smith S (2002) Improved optimization for the robust and accurate linear registration and motion correction of brain images. NeuroImage 17(2):825–841. 10.1016/s1053-8119(02)91132-8 12377157

[CR49] Jenkinson M, Beckmann CF, Behrens TEJ, Woolrich MW, Smith SM (2012) FSL. Neuroimage 62(2):782–790. 10.1016/j.neuroimage.2011.09.015 21979382

[CR50] Kandelʹ EI, Walker AE (1989) Functional and stereotactic neurosurgery. Plenum Medical Book Co.

[CR51] Klein A, Andersson J, Ardekani BA, Ashburner J, Avants B, Chiang MC, Christensen GE, Collins DL, Gee J, Hellier P, Song JH, Jenkinson M, Lepage C, Rueckert D, Thompson P, Vercauteren T, Woods RP, Mann JJ, Parsey RV (2009) Evaluation of 14 nonlinear deformation algorithms applied to human brain MRI registration. Neuroimage 46(3):786–802. 10.1016/j.neuroimage.2008.12.037 19195496 PMC2747506

[CR52] Kok CY, Lock C, Ang TY, Keong NC (2022) Modeling the properties of white matter tracts using diffusion tensor imaging to characterize patterns of injury in aging and neurodegenerative disease. Front Aging Neurosci 14:787516. 10.3389/fnagi.2022.787516 35572145 PMC9093601

[CR53] Lancaster JL, Glass TG, Lankipalli BR, Downs H, Mayberg H, Fox PT (1995) A modality-independent approach to spatial normalization of tomographic images of the human brain. Hum Brain Mapp 3(3):209–223. 10.1002/hbm.460030305

[CR54] Lau J (2022) Stereotactic spaces. In (pp. 169–184). 10.1016/B978-0-12-821861-7.00023-3

[CR55] Lau JC, Parrent AG, Demarco J, Gupta G, Kai J, Stanley OW, Kuehn T, Park PJ, Ferko K, Khan AR, Peters TM (2019) A framework for evaluating correspondence between brain images using anatomical fiducials. Hum Brain Mapp 40(14):4163–4179. 10.1002/hbm.24693 31175816 PMC6865720

[CR56] Liu Y, Dawant BM (2014) Automatic detection of the anterior and posterior commissures on MRI scans using regression forests. Annu Int Conf IEEE Eng Med Biol Soc 2014:1505–1508. 10.1109/EMBC.2014.6943887 25570255 PMC4422502

[CR57] Liu Y, Dawant BM (2015) Automatic localization of the anterior commissure, posterior commissure, and midsagittal plane in MRI scans using regression forests. IEEE J Biomed Health Inf 19(4):1362–1374. 10.1109/JBHI.2015.2428672 PMC4519399 25955855

[CR58] Liu Y, Collins RT, Rothfus WE (2001) Robust midsagittal plane extraction from normal and pathological 3-D neuroradiology images. IEEE Trans Med Imaging 20(3):175–192. 10.1109/42.918469 11341708

[CR59] Mandal PK, Jindal K, Roy S, Arora Y, Sharma S, Joon S, Goel A, Ahasan Z, Maroon JC, Singh K, Sandal K, Tripathi M, Sharma P, Samkaria A, Gaur S, Shandilya S (2023) SWADESH: a multimodal multi-disease brain imaging and neuropsychological database and data analytics platform. Front Neurol 14:1258116. 10.3389/fneur.2023.1258116 37859652 PMC10582723

[CR60] Martensson G, Ferreira D, Cavallin L, Muehlboeck JS, Wahlund LO, Wang C, Westman E, Alzheimer’s Disease Neuroimaging, I. (2019) AVRA: automatic visual ratings of atrophy from MRI images using recurrent convolutional neural networks. Neuroimage Clin 23:101872. 10.1016/j.nicl.2019.101872 31154242 PMC6545397

[CR61] Mazziotta JC, Toga AW, Evans A, Fox P, Lancaster J (1995) A probabilistic atlas of the human brain: theory and rationale for its development. The International Consortium for Brain Mapping (ICBM). Neuroimage 2(2):89–101 9343592 10.1006/nimg.1995.1012

[CR62] Minoshima S, Koeppe RA, Mintun MA, Berger KL, Taylor SF, Frey KA, Kuhl DE (1993) Automated detection of the intercommissural line for stereotactic localization of functional brain images. J Nucl Med 34(2):322–329 8429356

[CR63] Minoshima S, Koeppe RA, Frey KA, Kuhl DE (1994) Anatomic standardization: linear scaling and nonlinear warping of functional brain images. J Nucl Med 35(9):1528–1537 8071705

[CR64] Mottonen T, Katisko J, Haapasalo J, Tahtinen T, Kiekara T, Kahara V, Peltola J, Ohman J, Lehtimaki K (2015) Defining the anterior nucleus of the thalamus (ANT) as a deep brain stimulation target in refractory epilepsy: delineation using 3 T MRI and intraoperative microelectrode recording. Neuroimage Clin 7:823–829. 10.1016/j.nicl.2015.03.001 26082891 PMC4459042

[CR65] Niemann K, van den Boom R, Haeselbarth K, Afshar F (1999) A brainstem stereotactic atlas in a three-dimensional magnetic resonance imaging navigation system: first experiences with atlas-to-patient registration. J Neurosurg 90(5):891–901. 10.3171/jns.1999.90.5.0891 10223456

[CR66] Nowinski WL (2001) Modified Talairach landmarks. Acta Neurochir (Wien) 143(10):1045–1057. 10.1007/s007010170011 11685613

[CR67] Oba H, Yagishita A, Terada H, Barkovich AJ, Kutomi K, Yamauchi T, Furui S, Shimizu T, Uchigata M, Matsumura K, Sonoo M, Sakai M, Takada K, Harasawa A, Takeshita K, Kohtake H, Tanaka H, Suzuki S (2005) New and reliable MRI diagnosis for progressive supranuclear palsy. Neurology 64(12):2050–2055. 10.1212/01.WNL.0000165960.04422.D0 15985570

[CR68] Otake S, Taoka T, Maeda M, Yuh WT (2018) A guide to identification and selection of axial planes in magnetic resonance imaging of the brain. Neuroradiol J 31(4):336–344. 10.1177/1971400918769911 29671688 PMC6111434

[CR69] Ouachikh O, Chaix R, Sontheimer A, Coste J, Aider OA, Dautkulova A, Abdelouahab K, Hafidi A, Salah MB, Pereira B, Lemaire JJ (2024) Brain color-coded diffusion imaging: utility of ACPC reorientation of gradients in healthy subjects and patients. Comput Methods Programs Biomed 257:108449. 10.1016/j.cmpb.2024.108449 39378632

[CR70] Oxenford S, Roediger J, Neudorfer C, Milosevic L, Güttler C, Spindler P, Vajkoczy P, Neumann W-J, Kühn A, Horn A (2022) Lead-OR: a multimodal platform for deep brain stimulation surgery. eLife 11:e72929. 10.7554/eLife.72929 35594135 PMC9177150

[CR71] Pai PP, Mandal PK, Punjabi K, Shukla D, Goel A, Joon S, Roy S, Sandal K, Mishra R, Lahoti R (2020) BRAHMA: Population specific T1, T2, and FLAIR weighted brain templates and their impact in structural and functional imaging studies. Magn Reson Imaging 70:5–21. 10.1016/j.mri.2019.12.009 31917995

[CR72] Pallavaram S, Yu H, Spooner J, D’Haese PF, Bodenheimer B, Konrad PE, Dawant BM (2008) Intersurgeon variability in the selection of anterior and posterior commissures and its potential effects on target localization. Stereotact Funct Neurosurg 86(2):113–119. 10.1159/000116215 18270482 PMC2719970

[CR73] Pallavaram S, Dawant BM, Koyama T, Yu H, Neimat J, Konrad PE, D’Haese PF (2009) Validation of a fully automatic method for the routine selection of the anterior and posterior commissures in magnetic resonance images. Stereotact Funct Neurosurg 87(3):148–154. 10.1159/000209295 19321967 PMC2835380

[CR74] Pallavaram S, D’Haese PF, Lake W, Konrad PE, Dawant BM, Neimat JS (2015) Fully automated targeting using nonrigid image registration matches accuracy and exceeds precision of best manual approaches to subthalamic deep brain stimulation targeting in Parkinson disease. Neurosurgery 76(6):756–765. 10.1227/NEU.0000000000000714 25988929 PMC4438781

[CR75] Park JS, Chung MS, Park HS, Shin DS, Har DH, Cho ZH, Kim YB, Han JY, Chi JG (2010) A proposal of new reference system for the standard axial, sagittal, coronal planes of brain based on the serially-sectioned images. J Korean Med Sci 25(1):135–141. 10.3346/jkms.2010.25.1.135 20052359 PMC2800020

[CR76] Patel NK, Khan S, Gill SS (2008) Comparison of atlas- and magnetic-resonance-imaging-based stereotactic targeting of the subthalamic nucleus in the surgical treatment of Parkinson’s disease. Stereotact Funct Neurosurg 86(3):153–161. 10.1159/000120427 18334857

[CR77] Abbas SQ, Chi L, Chen Y-P (2023) Transformed domain convolutional neural network for Alzheimer’s disease diagnosis using structural MRI. Pattern Recognit 133:109031. 10.1016/j.patcog.2022.109031

[CR78] Ramirez J, Scott CJ, McNeely AA, Berezuk C, Gao F, Szilagyi GM, Black SE (2014) Lesion Explorer: a video-guided, standardized protocol for accurate and reliable MRI-derived volumetrics in Alzheimer’s disease and normal elderly. J Vis Exp 86 10.3791/50887 24797507 PMC4168739

[CR79] Roelants JA, Koning IV, Raets MM, Willemsen SP, Lequin MH, Steegers-Theunissen RP, Reiss IK, Vermeulen MJ, Govaert P, Dudink J (2016) A New Ultrasound Marker for Bedside Monitoring of Preterm Brain Growth. AJNR Am J Neuroradiol 37(8):1516–1522. 10.3174/ajnr.A4731 26988817 PMC7960273

[CR80] Ryan R, Sarno R, Suroto NS, Supriyanto MIA, Sihaj G, Susilo RI (2025) Effect of anterior commisure - posterior commisure reorientation for 3d aneurysm segmentation in the brain. icocseti 2025 - international conference on computer sciences, engineering, and technology innovation, proceeding

[CR81] Saint-Cyr JA, Hoque T, Pereira LC, Dostrovsky JO, Hutchison WD, Mikulis DJ, Abosch A, Sime E, Lang AE, Lozano AM (2002) Localization of clinically effective stimulating electrodes in the human subthalamic nucleus on magnetic resonance imaging. J Neurosurg 97(5):1152–1166. 10.3171/jns.2002.97.5.1152 12450038

[CR82] Sandor T, Tieman J, Ong HT, Moss MB, Jolesz F, Albert M (1994) Comparison of the precision of two standardized co-ordinate systems for the quantitation of brain anatomy: preliminary results. Neuroradiology 36(7):499–503. 10.1007/BF00593507 7845570

[CR83] Schaltenbrand G, Wahren W, Hassler RG (1977) Atlas for stereotaxy of the human brain, 2d, rev.enl. ed. edn edn. Thieme

[CR84] Schuurman PR, de Bie RM, Majoie CB, Speelman JD, Bosch DA (1999) A prospective comparison between three-dimensional magnetic resonance imaging and ventriculography for target-coordinate determination in frame-based functional stereotactic neurosurgery. J Neurosurg 91(6):911–914. 10.3171/jns.1999.91.6.0911 10584834

[CR85] Shah LM, McLean LA, Heilbrun ME, Salzman KL (2013) Intracranial hypotension: improved MRI detection with diagnostic intracranial angles. AJR Am J Roentgenol 200(2):400–407. 10.2214/AJR.12.8611 23345364

[CR86] Shah NJ, Abbas Z, Ridder D, Zimmermann M, Oros-Peusquens AM (2022) A novel MRI-based quantitative water content atlas of the human brain. Neuroimage 252:119014. 10.1016/j.neuroimage.2022.119014 35202813

[CR87] Starr PA, Vitek JL, DeLong M, Bakay RA (1999) Magnetic resonance imaging-based stereotactic localization of the globus pallidus and subthalamic nucleus. *Neurosurgery*. 10.1097/00006123-199902000-00031 9932883

[CR88] Talairach J, Tournoux P (1988) Co-planar stereotaxic atlas of the human brain: 3-dimensional proportional system : an approach to cerebral imaging. G. Thieme; Thieme Medical Publishers

[CR89] Thompson DK, Inder TE, Faggian N, Johnston L, Warfield SK, Anderson PJ, Doyle LW, Egan GF (2011) Characterization of the corpus callosum in very preterm and full-term infants utilizing MRI. NeuroImage 55(2):479–490. 10.1016/j.neuroimage.2010.12.025 21168519 PMC3035727

[CR90] Thung KH, Yap PT, Shen D (2017) Multi-stage Diagnosis of Alzheimer’s Disease with Incomplete Multimodal Data via Multi-task Deep Learning. Deep Learn Med Image Anal Multimodal Learn Clin Decis Support (2017) 10553:160–168. 10.1007/978-3-319-67558-9_19 29104963 PMC5666687

[CR91] Tian W, Zhang J, Chen J, Liu Y, Chen X, Wang N (2016) A quantitative study of intracranial hypotensive syndrome by magnetic resonance. Clin Neurol Neurosurg 141:71–76. 10.1016/j.clineuro.2015.12.014 26745515

[CR92] Vallat R (2018) Pingouin: statistics in Python. J Open Source Softw 3:1026. 10.21105/joss.01026

[CR93] Weiss KL, Pan H, Storrs J, Strub W, Weiss JL, Jia L, Eldevik OP (2003) Clinical brain MR imaging prescriptions in Talairach space: technologist- and computer-driven methods. AJNR Am J Neuroradiol 24(5):922–929 12748095 PMC7975799

[CR94] Yap CW, Yong C, Soon BKH (2023) The different shapes of the fourth ventricle. Clin Radiol 78(12):875–884. 10.1016/j.crad.2023.07.012 37604738

[CR95] Yoo TS, Ackerman MJ, Lorensen WE, Schroeder W, Chalana V, Aylward S, Metaxas D, Whitaker R (2002) Engineering and algorithm design for an image processing Api: a technical report on ITK–the Insight Toolkit. Stud Health Technol Inform 85:589–592 15458157

[CR96] Zhang Y, Brady M, Smith S (2001) Segmentation of brain MR images through a hidden Markov random field model and the expectation-maximization algorithm. IEEE Trans Med Imaging 20(1):45–57. 10.1109/42.906424 11293691

[CR97] Zhou T, Thung KH, Zhu X, Shen D (2019) Effective feature learning and fusion of multimodality data using stage-wise deep neural network for dementia diagnosis. Hum Brain Mapp 40(3):1001–1016 30381863 10.1002/hbm.24428 PMC6865441

[CR98] Zrinzo L, Zrinzo LV, Tisch S, Limousin PD, Yousry TA, Afshar F, Hariz MI (2008) Stereotactic localization of the human pedunculopontine nucleus: atlas-based coordinates and validation of a magnetic resonance imaging protocol for direct localization. Brain 131(6):1588–1598 18467343 10.1093/brain/awn075

