# Supplementary material for: ACPC-MAP: a protocol for manually aligning structural T1-weighted magnetic resonance images to the anterior commissure – posterior commissure plane according to clinical standards
Source: Brain Struct Funct. 2026 Aug 29;231(7):130. doi: 10.1007/s00429-026-03171-z (PMC13526096; doi:10.1007/s00429-026-03171-z)
Supplement: Supplementary file 1 [file 429_2026_3171_MOESM1_ESM.docx]

Supplementary Material

# Contents

# 1. Anatomical landmarks

# 2. Precursor Guideline

# 3. Verification Criteria for the Precursor Guideline

# 4. Data preparation and Preprocessing

# 5. Assessment of acpcdetect Transformation Matrix

# 6. Visual Assessment of Landmark Localization Error

# 7. Amendments

# 8. Examples of Minor Rotational Deviations

# 9. References

# 1.Anatomical Landmarks

In this section, we provide a brief overview of the anatomical landmarks used in the guideline, along with instructions on identifying these structures across all three cardinal planes.

## 1.1 Anterior Commissure (AC):

The anterior commissure (AC) is a compact bundle of myelinated fibers that connects various regions across both hemispheres of the brain (Datta, 2013; Standring & Gray, 2008). Due to its consistent and well-defined location, the AC serves as a major landmark for reference in both neurosurgical and radiological practices (Schaltenbrand et al., 1977; Seeger & Zentner, 2002; Talairach & Tournoux, 1988)

In the mid-sagittal plane, the AC can be identified as a small protrusion along the anterior wall of the third ventricle (Naidich et al., 1986; Standring & Gray, 2008). It often appears as an extension of the fornix in MRI images (Fig. 2A). In the axial plane, it is seen in the midline as a thick band of white matter, appearing as a hyperintense structure on T1-weighted images and connecting both hemispheres of the brain to form the classic “handlebar” appearance (Naidich et al., 1986) (Fig. 2B). In the coronal plane, AC can be identified as a thick white matter structure extends between the two hemispheres of the brain (Fig. 2C), positioned between the lateral ventricles above and the third ventricle below (Naidich et al., 1986).

**Figure 2. Anterior commissure:** The left panels display the appearance of the AC in the three cardinal planes (A) sagittal, (B) axial, and (C) coronal highlighting the region of interest (ROI), while the right panels provide enlarged views of the corresponding ROIs in each plane, emphasizing the key anatomical structures.

## 1.2. Posterior Commissure (PC):

The posterior commissure (PC) is a complex bundle of fibers that connects various structures in both cerebral hemispheres (Datta, 2013; Standring & Gray, 2008). It is one of the commonly used midline structures in neurosurgical references, owing to its consistent and well-defined location in the brain (Schaltenbrand et al., 1977; Seeger & Zentner, 2002; Talairach & Tournoux, 1988).

In the mid-sagittal plane, the posterior commissure (PC) can be identified as a small, "C"-shaped structure located on the posterior wall of the third ventricle, just above the midbrain and at the beginning of the cerebral aqueduct (Fig. 3A). In the axial plane a short band of hyperintense structure can be identified in the midline, connecting both cerebral hemispheres and contributing to the posterior boundary of the third ventricle (Fig. 3B). In the coronal plane, the PC appears as a short band in the midline, situated between the third ventricle above and the cerebral aqueduct below (Fig. 3C).

**Figure 3. Posterior commissure:** The left panels display the appearance of the PC in the three cardinal planes (A) sagittal, (B) axial, and (C) coronal highlighting the region of interest (ROI), while the right panels provide enlarged views of the corresponding ROIs in each plane, emphasizing the key anatomical structures

## 1.3.Superior Pontine notch:

The superior pontine notch is a subtle indentation located along the superior margin of the pons, visible in the sagittal plane. It serves as a key anatomical landmark, often used to demarcate the junction between the pons and the midbrain, also known as the pontomesencephalic junction (Ardekani & Bachman, 2009; Lau et al., 2019), frequently serving as a key imaging landmark in radiological assessments (Oba et al., 2005)

The superior pontine notch is easily identifiable in the mid-sagittal plane, situated at the junction between the midbrain above and the pons below, within the interpeduncular fossa (Fig. 4A). In the corresponding axial slice, the superior pontine notch can be found at the midline, located at the junction where the right and left cerebral peduncles meet (Fig. 4B). In the corresponding coronal slice, the superior pontine notch appears as a small midline opening at the pontomesencephalic junction, marking the transition zone between the midbrain above and the pons below (Fig. 4C).

**Figure 4. Superior Pontine Notch:** The left panels display the appearance of the superior pontine notch in the three cardinal planes (A) sagittal, (B) axial, and (C) coronal highlighting the region of interest (ROI), while the right panels provide enlarged views of the corresponding ROIs in each plane, emphasizing the key anatomical structures

## 1.4 Fastigium or Apex of the roof of the Fourth Ventricle:

The fastigium or fastigium cerebelli is the apex of the tent-shaped roof of the fourth ventricle in the brain (Matsushima et al., 1982; Salma et al., 2013). This apex marks the junction between the superior and inferior parts of the ventricular roof (Morris et al., 2022; Stratchko et al., 2016), serving as a key structural landmark for both the cerebellum and the brainstem.

The fastigium is best visualized in the midsagittal plane, where the two parts of the roof of the fourth ventricle converge (Fig.5A). At this junction, it appears as a protrusion into the cerebellum, marking the point where the superior and inferior portions of the ventricular roof meet. In the corresponding axial slice, the fastigium appears as a flat surface, forming one of the key boundaries of the fourth ventricle (Fig.5B). In the corresponding coronal slice, the fastigium appears at the center of the cerebellum, presenting as a curved apex of the roof of the fourth ventricle (Fig. 5C).

**Figure 5. Fastigium:** The left panels display the appearance of the Fastigium in the three cardinal planes (A) sagittal, (B) axial, and (C) coronal highlighting the region of interest (ROI), while the right panels provide enlarged views of the corresponding ROIs in each plane, emphasizing the key anatomical structures

## 1.5. Corpus callosum (CC):

The corpus callosum is the largest fibre pathway in the brain, linking the majority of the cerebral cortices of both hemispheres (Datta, 2013; Standring & Gray, 2008). It forms a significant portion of the roof of the lateral ventricles. Structurally, the corpus callosum is divided into five distinct parts, with the central and largest portion referred to as the trunk (Musiek, 1986). This trunk can be readily identified across all three imaging planes. usually located in the middle of the brain.

The entire length of the corpus callosum is best visualized in the mid-sagittal plane. On the flat medial surface of each hemisphere, a clearly visible, large, arched band with an upward convexity can be recognized, appearing hyperintense on T1-weighted images (Fig. 6A). In the axial plane, the middle two-thirds of the corpus callosum appear as a large X-shaped band of white matter in the midline, effectively connecting both hemispheres (Fig.6B). In the coronal plane, the trunk of the corpus callosum appears as a hammock-shaped bridge, extending into both hemispheres just above the lateral ventricles (Fig. 6C).

**Figure 6. Corpus Callosum:** The left panels display the appearance of the Corpus callosum in the three cardinal planes (A) sagittal, (B) axial, and (C) coronal highlighting the region of interest (ROI), while the right panels provide enlarged views of the corresponding ROIs in each plane, emphasizing the key anatomical structures

## 1.6. Falx Cerebri:

The falx cerebri is the largest infolding of the dura mater, the protective outer layer of the brain (Adeeb et al., 2012; Moore et al., 2014). This thin, crescent-shaped structure extends downward from the inner surface of the skull, running along the midline within the longitudinal fissure, which separates the brain into two cerebral hemispheres (Datta, 2013; Moore et al., 2014; Standring & Gray, 2008). In the sagittal view, the falx cerebri appears as a crescent-shaped structure (Standring & Gray, 2008). Along its upper convex margin runs the superior sagittal sinus, one of the largest venous channels in the brain (Datta, 2013; Moore et al., 2014; Standring & Gray, 2008). This close anatomical relationship between the falx cerebri and the superior sagittal sinus can be a useful landmark when tracing the falx cerebri in medical images.

Identifying the falx cerebri in the sagittal plane requires considerable effort. In the midsagittal plane, it presents as a thin, translucent structure with a glassy appearance. This subtlety can make it challenging to trace the falx accurately. However, careful observation allows for identification by following the inferior border of the superior sagittal sinus, which presents as a distinct hyperintense arch on T1-weighted images (Fig. 7A). In the axial plane, the falx cerebri can be traced within the longitudinal fissure, extending from the borders of the superior sagittal sinus (Fig. 7B). This sinus is characterized by a triangular hyperintense mass located at both the anterior and posterior ends of the brain in the midline (Adeeb et al., 2012; Standring & Gray, 2008), serving as a key landmark for identifying the falx cerebri. In the coronal view, the falx cerebri can be recognized as a slender sheet extending from the triangular mass of the superior sagittal sinus in the midline, effectively separating the two hemispheres of the brain (Fig. 7C).

**Figure 7. Falx cerebri and Superior Sagittal sinus:** The left panels display the appearance of the Falx and superior sagittal sinus in the three cardinal planes (A) sagittal, (B) axial, and (C) coronal, while the right panels highlighting both structures

# 2.Precursor Protocol

## 2.1. Anterior Commissure(AC):

**1:** Identify the Anterior Commissure (AC)

**Figure 8** Sagittal view of the Anterior commissure. The left panel presents a midsagittal view with the region of interest (ROI) highlighted, whereas the right panel offers an enlarged view of the ROI, emphasizing critical anatomical structures

**2:** Select a point at the posterior border of the Anterior Commissure (AC)

**Figure 9** Sagittal view of the Anterior commissure. The left panel presents a midsagittal view with the region of interest (ROI) highlighted, whereas the right panel offers an enlarged view of the ROI, emphasizing critical anatomical structures

3. Use the corresponding axial plane to ensure the annotated point is accurately positioned along the midline.

**Figure 10** Axial view of the Anterior commissure. The left panel presents a axial view with the region of interest (ROI) highlighted, whereas the right panel offers an enlarged view of the ROI, emphasizing critical anatomical structures

## 2.2. Posterior Commissure(PC):

1.Identify the Posterior commissure (PC)

**Figure 11** Sagittal view of the Posterior commissure. The left panel presents a midsagittal view with the region of interest (ROI) highlighted, whereas the right panel offers an enlarged view of the ROI, emphasizing critical anatomical structures

2. Select a point at the Anterior border of the Posterior Commissure (PC)

**Figure 12** Sagittal view of the Posterior commissure. The left panel presents a midsagittal view with the region of interest (ROI) highlighted, whereas the right panel offers an enlarged view of the ROI, emphasizing critical anatomical structures

3. Use the corresponding axial plane to ensure the annotated point is accurately positioned along the midline

**Figure 13** Axial view of the Posterior commissure. The left panel presents a axial view with the region of interest (ROI) highlighted, whereas the right panel offers an enlarged view of the ROI, emphasizing critical anatomical structures

## 2.3. Superior Pontine notch:

1. Identify the superior pontine notch

2. Place the first midline point in the superior pontine notch at the junction where the midbrain and pons meet

**Figure 14** Sagittal view demonstrating the superior pontine notch. The left panel depicts a midsagittal perspective highlighting the region of interest (ROI), while the right panel provides a magnified view of the ROI, clearly emphasizing critical anatomical structures and specifically highlighting the superior pontine notch

3. Use the corresponding axial slice to ensure the point is correctly positioned in the midline, where the two (left and right) peduncles join to form the interpeduncular angle

**Figure 15** Axial view demonstrating the superior pontine notch. The left panel depicts a axial perspective highlighting the region of interest (ROI), while the right panel provides a magnified view of the ROI, clearly emphasizing critical anatomical structures and specifically highlighting the superior pontine notch

## 2.4. Fastigium or Apex of the Roof of fourth ventricle:

1. Identify the fastigium, also known as the apex of the roof of the fourth ventricle

2. Place the second midline point in the fastigium

**Figure 16** Sagittal view demonstrating the fastigium. The left panel depicts a midsagittal perspective highlighting the region of interest (ROI), while the right panel provides a magnified view of the ROI, clearly emphasizing critical anatomical structures and specifically highlighting the fastigium

3. Use the corresponding axial slice to ensure the point is accurately placed along the midline in the fastigium

**Figure 17** Axial view demonstrating the fastigium. The left panel depicts a axial perspective highlighting the region of interest (ROI), while the right panel provides a magnified view of the ROI, clearly emphasizing critical anatomical structures and specifically highlighting the fastigium

## 2.5. Anterior and Posterior Points in the Falx Cerebri

1. Identify the corpus callosum.

2. Draw a line at the highest point of the upper border of the corpus callosum.

**Figure 18** Sagittal view illustrating the corpus callosum with a horizontal line marking its superior-most boundary

3. Use the corresponding axial plane to place two midline points (the 3rd and 4th points): one at the anterior end of the falx cerebri (refer to Figure.19) and another at the posterior end, where the falx cerebri splits to enclose the superior sagittal sinus

**Figure 19** Axial view demonstrating the anterior and posterior falx cerebri at the superior-most level of the corpus callosum.

# 3.Verification of AC_PC alignment

After realigning the structural image to the AC-PC plane, it is essential to verify that the image is properly aligned. This involves checking for any rotations in the X, Y, and Z Axes, commonly referred to as pitch, roll, and yaw.

## 3.1. Verifying pitch:

1.After the successful realignment, the Anterior Commissure (AC) and the Posterior Commissure (PC) should be at the same level. Refer to Figure. 20 For the differences before and after AC-PC alignment

**Figure 20** Verification of Pitch. From left to right, the first panel displays the midsagittal plane with the positions of the AC and the PC following improper realignment, highlighting noticeable misalignment. The right panel shows the same plane after successful realignment, with the AC and PC properly aligned at the expected anatomical level.

2.The presence of the Anterior Commissure (AC) and the Posterior Commissure (PC) in the same axial slice will create the characteristic keyhole appearance of the third ventricle. Refer to Figure.32 for the difference before and after the AC-PC alignment, and note the keyhole appearance after the AC-PC alignment (Figure 21 right panel)

**Figure 21** Verification of Pitch. Axial planes illustrating the appearance of the AC and PC before (left panel) and after (right panel) successful AC-PC realignment. In the right panel, the simultaneous presence of both AC and PC within the same slice confirms proper realignment. Additionally, the characteristic keyhole shape of the third ventricle further supports accurate anatomical alignment.

## 3.2. Verifying roll:

1. Roll verification can be performed by examining the appearance of the eyeballs in axial images. In a correctly realigned scan, the eyeballs should appear symmetrical and evenly shaped within the selected axial slice. In Figure 22, note the uneven appearance of the eyeballs in the left panel, indicating misalignment, compared to the right panel, where their even, symmetrical appearance confirms successful AC-PC realignment.

**Figure 22** Verification of Roll. Axial planes illustrating the appearance of the eyeballs before (left panel) and after (right panel) successful AC-PC realignment.

## 3.3. Verifying yaw:

1. We can verify the yaw by placing the vertical crosshair. The vertical line should align with the longitudinal fissure or midline of the brain in the axial plane. Refer to Figure. 23 left Panel where the longitudinal fissure and vertical line (yellow line) are not properly aligned before the AC-PC alignment. In contrast, Right Panel demonstrates proper alignment of the longitudinal fissure with the vertical line (yellow line), indicating successful midline alignment

**Figure 23** Verification of Yaw. Axial planes illustrating midline deviations before (left panel) and after (right panel) successful AC-PC realignment. Note how the midline shift in the left panel is corrected in the right panel, confirming proper alignment

# 4. Data preparation and Preprocessing

Table 1: Summary of rotation angle values extracted from the Abrim dataset. The extreme values Table1A (minimum, mean, and maximum) were randomly sign-flipped and renamed as Rmin, Ravg, Rmax, according to new (minimum, mean, and maximum) values Table 1B .

**Table 1 A**

|  | x | y | z |
| --- | --- | --- | --- |
| count | 226 | 226 | 226 |
| **mean** | **10.0** | **-0.0** | **0.0** |
| std | 4.0 | 1.0 | 1.0 |
| **min** | **-8.0** | **-4.0** | **-2.0** |
| 25% | 8.0 | -1.0 | -0.0 |
| 50% | 10.0 | -0.0 | 0.0 |
| 75% | 12.0 | 0.0 | 1.0 |
| **max** | **25.0** | **4.0** | **3.0** |

**Table 1 B**

|  | x | y | z |
| --- | --- | --- | --- |
| Rmin | -10.0 | 0.0 | 0.0 |
| Ravg | 8.0 | -4.0 | 2.0 |
| Rmax | 25.0 | 4.0 | -3.0 |

**Figure 24** Preprocessing Stages for Data Preparation. This figure illustrates the key preprocessing steps involved in preparing the data for the evaluation process.

# 5.Assessment of acpcdetect Transformation Matrix

Table 2. RMSD values (mm) derived from two acpcdetect rotation matrices (the original output matrix and the Y–Z sign-flipped variant), quantifying the deviation of the acpcdetect transformation from the baseline.

|  | *Rmin* | *Ravg* | *Rmax* | *Mean* |
| --- | --- | --- | --- | --- |
| acpcdetect -original | 4.72 | 7.40 | 8.68 | 6.93 |
| acpcdetect- YZ flipped | 4.42 | 2.46 | 3.04 | 3.31 |

Table 3. Rotation angles (degrees) for all three axes extracted from two acpcetect matrix variants (the original output matrix and the Y–Z sign-flipped variant) and compared with the baseline. Absolute differences relative to the baseline are reported in brackets.

|  | *Rmin* | | | *Ravg* | | | *Rmax* | | |
| --- | --- | --- | --- | --- | --- | --- | --- | --- | --- |
|  | ***X*** | ***Y*** | ***Z*** | ***X*** | ***Y*** | ***Z*** | ***X*** | ***Y*** | ***Z*** |
| ***Baseline*** | ***-11.65*** | ***-0.35*** | ***0.09*** | ***8.54*** | ***3.48*** | ***2.26*** | ***27.27*** | ***-0.74*** | ***-4.58*** |
| acpcdetect -original | -15.96(4.31) | 2.02(2.37) | -0.65(0.74) | 6.04(2.50) | -3.57(7.05) | -1.02(3.29) | 24.26(3.01) | 1.97(2.71) | 4.14(8.72) |
| acpcdetect-YZ flipped | -15.96(4.31) | -2.02(1.66) | 0.65(0.56) | 6.04(2.50) | 3.57(0.10) | 1.02(1.24) | 24.26(3.01) | -1.97(1.23) | -4.14(0.44) |

# 6. Visual Assessment of Landmark Localization Error

**Figure 25** Visual Comparison of the Automated Algorithm Performance Versus the Baseline Developed Using the Proposed Manual Guideline Under the Maximum Rotation Condition (Rmax). The left panels display sagittal sections highlighting the region of interest (ROI), while the right panels present enlarged views of the ROI for detailed comparison. As illustrated in panels A to C: A. Center of the anterior commissure (AC) identified by the automated AC-PC detection algorithm (blue dot) versus the actual center of AC (yellow) in the same slice. B. Center of the posterior commissure (PC) identified by the automated algorithm (blue dot) versus the actual center of PC (yellow). C. Schaltenbrand-version AC-PC line derived from the automatically detected AC and PC (blue) compared to the AC-PC line (reddish orange) derived from true centers of AC and PC (yellow)

**Figure 26** Visual Comparison of the Automated Algorithm Performance Versus the Baseline Developed Using the Proposed Manual Guideline Under the Minumum Rotation Condition (Rmin). The left panels display sagittal sections highlighting the region of interest (ROI), while the right panels present enlarged views of the ROI for detailed comparison. As illustrated in panels A to C: A. Center of the anterior commissure (AC) identified by the automated AC-PC detection algorithm (blue dot) versus the actual center of AC (yellow) in the same slice. B. Center of the posterior commissure (PC) identified by the automated algorithm (blue dot) versus the actual center of PC (yellow). C. Schaltenbrand-version AC-PC line derived from the automatically detected AC and PC (blue) compared to the AC-PC line (reddish orange) derived from true centers of AC and PC (yellow)

**Figure 27** Visual Comparison of the Automated Algorithm Performance Versus the Baseline Developed Using the Proposed Manual Guideline Under the Average Rotation Condition (Ravg). The left panels display sagittal sections highlighting the region of interest (ROI), while the right panels present enlarged views of the ROI for detailed comparison. As illustrated in panels A to C: A. Center of the anterior commissure (AC) identified by the automated AC-PC detection algorithm (blue dot) versus the actual center of AC (yellow) in the same slice. B. Center of the posterior commissure (PC) identified by the automated algorithm (blue dot) versus the actual center of PC (yellow). C. Schaltenbrand-version AC-PC line derived from the automatically detected AC and PC (blue), only visible in panels A and B. compared to the AC-PC line (reddish orange) derived from true centers of AC and PC (yellow)

# 7. Guideline Amendments

Based on a detailed analysis of the experimental results and feedback from both expert and non-expert observers, three amendments were made to the precursor guideline. Below are the justifications for each change and the corresponding modifications implemented in the guidelines:

### Amendment 1 – Final guideline step 2.2.3: AC-PC line

While the results from manual observers were generally accurate, small deviations did occur. Due to the close proximity of the AC and PC structures, even minor discrepancies can lead to significant rotational errors in the final realignment. [Figure 39 - 41]. Therefore, accurately defining the AC-PC line is critical. To address this issue, we have introduced an additional step into the final guideline specifically, Step 2.3.3. This step emphasizes the importance of precise AC-PC line identification, supported by visual aids to enhance accuracy and consistency.

**Figure 28** Comparison of AC-PC Line Definitions. The left panel presents a midsagittal section demonstrating the variation between the two AC-PC lines within the region of interest. The right panel provides a magnified view highlighting the differences between the baseline AC-PC line (reddish-orange) and the AC-PC line determined by Nonexpert-2 (yellow). Notice the discrepancy in identifying the AC, which consequently leads to a larger angular difference.

**Figure 29** Comparison of AC-PC Line Definitions. The left panel presents a midsagittal section demonstrating the variation between the two AC-PC lines within the region of interest. The right panel provides a magnified view highlighting the differences between the baseline AC-PC line (reddish-orange) and the AC-PC line determined by Nonexpert-4 (yellow). Notice the discrepancy in identifying the PC, which consequently leads to a larger angular difference.

**Figure 30** Comparison of AC-PC Line Definitions. The left panel presents a midsagittal section demonstrating the variation between the two AC-PC lines within the region of interest. The right panel provides a magnified view highlighting the differences between the baseline AC-PC line (reddish-orange) and the AC-PC line determined by expert-1 (yellow)

**Figure 31** Comparison of AC-PC Line Definitions. The left panel presents a midsagittal section demonstrating the variation between the two AC-PC lines within the region of interest. The right panel provides a magnified view highlighting the differences between the baseline AC-PC line (reddish-orange) and the AC-PC line determined by expert-1 (yellow)

### Amendment 2 -final guideline Step 2.6: Identifying midpoints 3 and 4

Although all manual observers correctly identified midpoint locations 3 and 4, including non-expert 1, who caused notable deviations of 28.68 mm and 23.63 mm by skipping a step in the precursor guideline (Specifically protocol step 2.5 (2)), they all encountered difficulties in maintaining correspondence. For instance, they struggled to place a landmark point precisely at the junction between the falx cerebri and the superior sagittal sinus in the axial plane at the highest level of the corpus callosum. Although this minor degree of inaccuracy is expected due to the inherent challenges of tracing delicate, sheet-like structures in the brain using MR images (Liu & Dawant, 2015), we aim to provide additional clarification to improve the identification of midpoints 3 and 4 in the precursor guideline. To address this, we revised the guideline to include clearer instructions for identifying these two midpoints within the falx cerebri. By utilizing visual aids, we have broken down the instructions into three sub-steps (2.2.6 in final guideline), enhancing clarity and improving the accuracy of landmark identification.

### Amendment 3 - final guideline 2.3: all three verification criteria

Another significant refinement was made to the verification criteria of the precursor guideline. In the precursor guideline, we initially adopted verification criteria from Ramirez et al. (Ramirez et al., 2014) to validate AC-PC realignment. While these criteria were effective at identifying large deviations, our results and feedback from manual observers indicated that they may lack sensitivity to smaller misalignments, particularly around the X-axis, which is crucial for accurate AC-PC alignment.

To improve sensitivity to smaller errors, we revised the three verification criteria for more precise AC-PC alignment detection. In the precursor guideline, verification of pitch or rotation around the X-axis involved visually confirming that the AC and PC were positioned at the same level on both sagittal and axial planes. In the sagittal plane, this was confirmed by drawing a straight line connecting the centres of AC to the PC, (Figure 20) while in the axial plane, alignment was verified by ensuring the AC and PC appeared on the same slice, creating the characteristic "keyhole" appearance of the third ventricle(Figure 21) (Ramirez et al., 2014). However, given that the AC is larger than the PC approximately 3 mm versus 2 mm in sagittal diameter each structure can span more than one slice in images with a 1 mm isotropic voxel resolution, as a result, evaluating a single slice may overlook subtle misalignments. Therefore, we now recommend examining three consecutive slices in the axial plane to confirm precise AC-PC realignment and assessing the full diameter of both the AC and PC in the sagittal plane. (Manuscript figures 15,16) This optimized approach improves detection of finer misalignments and supports more accurate realignment verification.

In the precursor guideline, roll verification depended on visually assessing eyeball levels (Ramirez et al., 2014), where visual aids illustrated balanced eyeballs as a cue for accurate AC-PC realignment and unbalanced eyeballs as an indicator of misalignment. While this visual cue is effective for detecting large errors, it may miss smaller, subtler misalignments that are more difficult to detect. To address this, we optimized the visual aids to illustrate changes associated with smaller errors, making the verification process more sensitive to minor discrepancies. Additionally, we incorporated the coronal plane in the evaluation (Hamilton et al., 2017), providing a more comprehensive perspective to detect and correct subtle alignment errors. This enhanced approach allows for finer accuracy in verifying roll alignment.(Manuscript figures 17,18) We adopted a similar approach for verifying yaw in the precursor guideline, the axial view example showing a large Z-axis rotational deviation has been replaced with an example image highlighting smaller deviations, enabling the detection of more subtle misalignments (Manuscript figure 19) (Hamilton et al., 2017; Ramirez et al., 2014)

# 8.Examples of Minor rotational deviations

The verification criteria outlined in our initial guideline assist in visually validating the results of manual AC-PC realignment. However, the examples we provided are particularly sensitive to larger errors, potentially limiting their effectiveness in detecting subtle misalignments. To visually illustrate examples of smaller deviations around the X, Y, and Z axes, we first realigned the MNI152 T1-weighted image, with a 1mm isotropic voxel resolution, to the AC-PC plane following the precursor guideline. Using this AC-PC alignment as the baseline, we then applied incremental rotations to the image from -3 to +3 degrees (i.e., -3, -2, -1, 1, 2, 3) along each of the three axes.

**Figure 32** Axial plane images demonstrating rotational deviations around the X-axis relative to the correctly aligned AC-PC reference image. The left panels illustrates images with negative rotational deviations (-1°, -2°, -3°), the middle panels shows the precisely realigned AC-PC plane, and the right panels presents images with positive rotational deviations (+1°, +2°, +3°). Notably, despite these deviations, the AC-PC landmarks consistently appear in the same axial plane across all images. This consistency suggests that relying solely on the visual confirmation of AC and PC appearing in a single axial slice may not effectively detect subtle misalignments.

**Figure 33** Axial planes illustrating rotational deviations around the Y-axis relative to the AC-PC realigned image. The left panels demonstrates negative rotational deviations (-1°, -2°, -3°), highlighting the visual effect where the right eyeball appears larger than the left. The middle panels represents the correctly realigned AC-PC plane, characterized by symmetry and equal appearance of both eyeballs. Conversely, the right panels illustrates positive rotational deviations (+1°, +2°, +3°), resulting in the left eyeball appearing larger than the right.

**Figure 34** Coronal planes illustrating rotational deviations around the Y-axis relative to the AC-PC realigned reference image. The left panel demonstrates negative rotational deviations (-1°, -2°, -3°), with the vertical crosshair shifted to the right of midline. The central panel depicts the accurately realigned AC-PC plane, with the vertical crosshair precisely positioned at the midline. The right panel illustrates positive rotational deviations (+1°, +2°, +3°), where the vertical crosshair shifts to the left of midline.

**Figure 35** Axial planes illustrating rotational deviations around the Z-axis relative to the accurately aligned AC-PC plane. The left panels presents images demonstrating negative rotational deviations (-1°, -2°, -3°), characterized by the vertical crosshair shifting progressively to the left. The middle panels depicts the precisely realigned AC-PC plane, where the vertical crosshair is centered accurately along the midline. The right panels exhibits images illustrating positive rotational deviations (+1°, +2°, +3°), with the vertical crosshair progressively shifting to the right.

# 9.References

Adeeb, N., Mortazavi, M. M., Tubbs, R. S., & Cohen-Gadol, A. A. (2012). The cranial dura mater: a review of its history, embryology, and anatomy. *Childs Nerv Syst*, *28*(6), 827–837. <https://doi.org/10.1007/s00381-012-1744-6>

Ardekani, B. A., & Bachman, A. H. (2009). Model-based automatic detection of the anterior and posterior commissures on MRI scans. *Neuroimage*, *46*(3), 677–682. <https://doi.org/10.1016/j.neuroimage.2009.02.030>

Datta, A. (2013). *Essential of the Human Anatomy,Neuroanatomy*. Current Books International.

Hamilton, L. S., Chang, D. L., Lee, M. B., & Chang, E. F. (2017). Semi-automated Anatomical Labeling and Inter-subject Warping of High-Density Intracranial Recording Electrodes in Electrocorticography. *Front Neuroinform*, *11*, 62. <https://doi.org/10.3389/fninf.2017.00062>

Lau, J. C., Parrent, A. G., Demarco, J., Gupta, G., Kai, J., Stanley, O. W., Kuehn, T., Park, P. J., Ferko, K., Khan, A. R., & Peters, T. M. (2019). A framework for evaluating correspondence between brain images using anatomical fiducials. *Hum Brain Mapp*, *40*(14), 4163–4179. <https://doi.org/10.1002/hbm.24693>

Matsushima, T., Rhoton, A. L., Jr., & Lenkey, C. (1982). Microsurgery of the fourth ventricle: Part 1. Microsurgical anatomy. *Neurosurgery*, *11*(5), 631–667. <https://doi.org/10.1227/00006123-198211000-00008>

Moore, K. L., Dalley, A. F., & Agur, A. M. R. (2014). *Clinically oriented anatomy* (Seventh edition ed.). Wolters Kluwer Health/Lippincott Williams & Wilkins.

Morris, J. A., Gilbert, B. C., Parker, W. T., & Forseen, S. E. (2022). Anatomy of the Ventricles, Subarachnoid Spaces, and Meninges. *Neuroimaging Clinics of North America*, *32*(3), 577–601. <https://doi.org/https://doi.org/10.1016/j.nic.2022.04.005>

Musiek, F. E. (1986). Neuroanatomy, neurophysiology, and central auditory assessment. Part III: Corpus callosum and efferent pathways. *Ear Hear*, *7*(6), 349–358. <https://doi.org/10.1097/00003446-198612000-00001>

Naidich, T. P., Daniels, D. L., Pech, P., Haughton, V. M., Williams, A., & Pojunas, K. (1986). Anterior commissure: anatomic-MR correlation and use as a landmark in three orthogonal planes. *Radiology*, *158*(2), 421–429. <https://doi.org/10.1148/radiology.158.2.3941867>

Oba, H., Yagishita, A., Terada, H., Barkovich, A. J., Kutomi, K., Yamauchi, T., Furui, S., Shimizu, T., Uchigata, M., Matsumura, K., Sonoo, M., Sakai, M., Takada, K., Harasawa, A., Takeshita, K., Kohtake, H., Tanaka, H., & Suzuki, S. (2005). New and reliable MRI diagnosis for progressive supranuclear palsy. *Neurology*, *64*(12), 2050–2055. <https://doi.org/10.1212/01.WNL.0000165960.04422.D0>

Ramirez, J., Scott, C. J., McNeely, A. A., Berezuk, C., Gao, F., Szilagyi, G. M., & Black, S. E. (2014). Lesion Explorer: a video-guided, standardized protocol for accurate and reliable MRI-derived volumetrics in Alzheimer's disease and normal elderly. *J Vis Exp*(86). <https://doi.org/10.3791/50887>

Salma, A., Yeremeyeva, E., Baidya, N. B., Sayers, M. P., & Ammirati, M. (2013). An endoscopic, cadaveric analysis of the roof of the fourth ventricle. *Journal of Clinical Neuroscience*, *20*(5), 710–714. <https://doi.org/https://doi.org/10.1016/j.jocn.2012.05.051>

Schaltenbrand, G., Wahren, W., & Hassler, R. G. (1977). *Atlas for stereotaxy of the human brain* (2d, rev. and enl. ed.). Thieme.

Seeger, W., & Zentner, J. (2002). *Neuronavigation and Neuroanatomy* (1st ed.). Springer Vienna : Imprint: Springer,. <https://doi.org/10.1007/978-3-7091-6101-2>

Standring, S., & Gray, H. (2008). *Gray's anatomy : the anatomical basis of clinical practice* (40th , anniversary ed.). Churchill Livingstone/Elsevier.

Stratchko, L., Filatova, I., Agarwal, A., & Kanekar, S. (2016). The Ventricular System of the Brain: Anatomy and Normal Variations. *Semin Ultrasound CT MR*, *37*(2), 72–83. <https://doi.org/10.1053/j.sult.2016.01.004>

Talairach, J., & Tournoux, P. (1988). *Co-planar stereotaxic atlas of the human brain : 3-dimensional proportional system : an approach to cerebral imaging*. G. Thieme ; Thieme Medical Publishers.
